# Resurgence of Chern–Simons Theory at the Trivial Flat Connection

**DOI:** 10.1007/s00220-024-05149-6

**Published:** 2024-12-18

**Authors:** Stavros Garoufalidis, Jie Gu, Marcos Mariño, Campbell Wheeler

**Affiliations:** 1 Department of Mathematics, International Center for Mathematics, Southern University of Science and Technology, Shenzhen, China; 2 Département de Physique Théorique, Université de Genève, Université de Genève, 1211 Genève 4, Switzerland; 3 Shing-Tung Yau Center and School of Physics, Southeast University, Nanjing, 210096 China; 4 Section de Mathématiques et Département de Physique Théorique, Université de Genève, 1211 Genève 4, Switzerland; 5 Institut des Hautes Études Scientifiques, Le Bois-Marie 35 rte de Chartres, 91440 Bures-sur-Yvette, France

## Abstract

Some years ago, it was conjectured by the first author that the Chern–Simons perturbation theory of a 3-manifold at the trivial flat connection is a resurgent power series. We describe completely the resurgent structure of the above series (including the location of the singularities and their Stokes constants) in the case of a hyperbolic knot complement in terms of an extended square matrix (*x*, *q*)-series whose rows are indexed by the boundary parabolic $\textrm{SL}_2(\mathbb {C})$-flat connections, including the trivial one. We use our extended matrix to describe the Stokes constants of the above series, to define explicitly their Borel transform and to identify it with state–integrals. Along the way, we use our matrix to give an analytic extension of the Kashaev invariant and of the colored Jones polynomial and to complete the matrix valued holomorphic quantum modular forms as well as to give an exact version of the refined quantum modularity conjecture of Zagier and the first author. Finally, our matrix provides an extension of the 3D-index in a sector of the trivial flat connection. We illustrate our definitions, theorems, numerical calculations and conjectures with the two simplest hyperbolic knots.

## Introduction

### Resurgence of Chern–Simons perturbation theory

Quantum Topology originated by Jones’s discovery of the famous polynomial invariant of a knot [Jon87], followed by Witten’s 3-dimensional interpretation of the Jones polynomial by means of a gauge theory with a topological (i.e., metric independent) Chern–Simons action [Wit89]. The connection between this topological quantum field theory and the Jones polynomial appears both on the level of the exact partition function and its perturbative expansion which both determine, and are determined by, the (colored) Jones polynomial. Indeed, the exact partition function on the complement of a knot colored by the defining representation of the gauge group $\textrm{SU}(2)$ at level *k* coincides with the value of the Jones polynomial at the complex root of unity $e^{2 \pi i/(k+2)}$. On the other hand, the perturbative expansion along the trivial flat connection $\sigma _0$ is a formal power series $\Phi ^{(\sigma _0)}(h) \in \mathbb {Q}[[h]]$ whose coefficients are Vassiliev knot invariants which are determined by the colored Jones polynomial of a knot expanded as a power series in *h* where $q=e^h$ [BN95]. More generally, the loop expansion of the colored Jones polynomial is a formal power series $\Phi ^{(\sigma _0)}(x,h) \in \mathbb {Q}(x)[[h]]$ introduced by Rozansky [Roz98] and further studied by Kricker [Kri, GK04], where $x=q^n$ plays the role of the monodromy of the meridian. An important feature of the power series $\Phi ^{(\sigma _0)}(x,h)$ is that it is determined by (but also uniquely determines) the colored Jones polynomial. Likewise, the power series $\Phi ^{(\sigma _0)}(h)$ is determined by (and determines) the Kashaev invariant of a knot [Kas95], interpreted as an element of the Habiro ring [Hab08].

In [Gar08a] the first author conjectured that the factorially divergent formal power series $\Phi ^{(\sigma _0)}(h)$ is resurgent, whose Borel transform has singularities arranged in a peacock pattern, and can be re-expanded in terms of the perturbative series $\Phi ^{(\sigma )}(h)$ corresponding to the remaining non-trivial flat connections of the Chern-Simons action. Although this is a well-defined statement, resurgence was a bit of the surprise and a mystery. We should point out that the above series are well-defined (for $\sigma \ne \sigma _0$ via formal Gaussian integration using as input an ideal triangulation of a 3-manifold [DG13], and for $\sigma =\sigma _0$ using the Kashaev invariant itself) and their coefficients are (up to multiplication by a power of $2 \pi i$) algebraic numbers. However a numerical computation of their coefficients is difficult (about 280 coefficients can be obtained for the simplest hyperbolic knot), hence it is difficult to numerically study them beyond the nearest to the origin singularity of their Borel transform.

The resurgence question has attracted a lot of attention in mathematics and mathematical physics and some aspects of it were discussed by Jones [Jon09], Witten [Wit11], Gukov, Putrov and the third author [GMnP], Costin and the first author [CG11] and Sauzin [Sau15]. Further aspects of resurgence in Chern–Simons theory were studied in [Mn14, GMnP, GH18, GZ23, GZ24].

When $\sigma \ne \sigma _0$, the resurgence structure of the series $\Phi ^{(\sigma )}(h)$ was given explicitly in [GGMn21], where it was found that the location of the singularities was arranged in a peacock pattern, and the Stokes constants were integers. The latter were fully described by an $r \times r$ matrix ${\textbf{J}}^\text {red}(q)$. The passage from a vector $(\Phi ^{(\sigma )}(h))_\sigma$ of power series to a matrix ${\textbf{J}}^\text {red}(q)$ is inevitable, and points out to the possibility that the non-perturbative partition function of a theory yet-to-be defined and its corresponding perturbative expansion is matrix-valued and *not* vector-valued, as was discussed in detail in [GZ24, GZ23]. Let us summarise some key properties of the matrix ${\textbf{J}}^\text {red}(q)$.

**Linear** *q*-**difference equation.** The entries of ${\textbf{J}}^\text {red}(q)$ are *q*-series with integer coefficients defined for $|q| \ne 1$. The matrix ${\textbf{J}}^\text {red}(q)$ is a fundamental solution of a linear *q*-difference equation of order *r*, and its rows are labeled by the set of nontrivial $\sigma$.

**Asymptotics in sectors:** *q*-**Stokes phenomenon.** The function ${\textbf{J}}^\text {red}(e^{2\pi {\textrm{i}}\tau })$ as $\tau$ approaches zero in a fixed cone, has a full asymptotic expansion as a sum of power series in $\tau$, times power series in $e^{-2\pi {\textrm{i}}/\tau }$. However, passing from one cone to an adjacent one changes the $e^{-2\pi {\textrm{i}}/\tau }$-series. The dependence of the asymptotics on the cone is the *q*-Stokes phenomenon, analogous to the well-studied Stokes phenomenon in the theory of linear differential equations with polynomial coefficients (see, e.g., [Sib90]). In our case, the *q*-Stokes phenomenon is a consequence of the fact that ${\textbf{J}}^\text {red}(q)$ is a fundamental matrix solution to a linear *q*-difference equation.

**Analyticity.** The product $W(\tau )$ of ${\textbf{J}}^\text {red}({\tilde{q}})$ with a diagonal automorphy factor and with ${\textbf{J}}^\text {red}(q)$, when $q=e^{2\pi i \tau }$ and ${\tilde{q}}=e^{-2\pi i/\tau }$, although defined for $\tau \in \mathbb {C}{\setminus }\mathbb {R}$, equals to a matrix of state-integrals and hence it analytically extends to $\tau$ in the cut plane $\mathbb {C}'=\mathbb {C}{\setminus } (-\infty , 0]$. A distinguished $(\sigma _1,\sigma _1)$ entry of $W(\tau )$, where $\sigma _1$ is the geometric representation of a hyperbolic 3-manifold, is the Andersen–Kashaev state-integral [AK14]. The latter is often identified with the unknown partition function of complex Chern–Simons theory. Thus, analyticity of *W* is interpreted as a factorisation property of state-integrals, or as a matrix-valued holomorphic quantum modular form [GZ24, Zagb].

**Borel resummation.** The matrix $W(\tau )$ coincides (in a suitable ray) to the Borel resummation of the matrix of perturbative series. In particular, the Borel resummation of the perturbative series is *not* a *q*-series as has been claimed repeatedly in some physics literature, but rather a bilinear combination of *q*-series and ${\tilde{q}}$-series.^1^

**Relation with the 3D-index.** The 3D-index of Dimofte–Gaiotto–Gukov can be expressed bilinearly in terms of ${\textbf{J}}^\text {red}(q)$ and ${\textbf{J}}^\text {red}(q^{-1})$. A detailed conjecture is given in see [GGMn23, Conj.4].

*x*-**extension.** There is an extension of the above invariants by a nonzero complex number *x*, which measures the monodromy of the meridian in the case of a knot complement, and extends the *q*-series to functions of (*x*, *q*), where *x* behaves like a Jacobi variable. This results in a matrix ${\textbf{J}}^\text {red}(x,q)$ whose properties extend those of the matrix ${\textbf{J}}^\text {red}(q)$ and were studied in detail in [GGMn23].

### A summary of our results

Our goal is to describe the Stokes constants and the resurgent structure of the missing asymptotic series $\Phi ^{(\sigma _0)}(h)$ in terms of completing the matrix ${\textbf{J}}^\text {red}(x,q)$ to a square matrix with one extra row (namely $(1,0,\dots ,0)^T$) and column, whose distinguished $(\sigma _0,\sigma _1)$ entry is conjecturally the Gukov–Manolescu series [GM21] (evaluated at $x=1$), and the remaining series in the top row are the descendants of the Gukov-Manolescu series.

Along the way of solving the resurgence problem for the $\Phi ^{(\sigma _0)}(h)$ series, we solve several related problems, which we now discuss.

- **A** *q*-**series that sees** $\Phi ^{(\sigma _0)}(h)$. This is a problem raised by Gukov and his collaborators (see e.g. [GPPV20, GM21]). More precisely, our Resurgence Conjecture 5 implies that the asymptotics as $q=e^{2\pi {\textrm{i}}\tau }$ and $\tau \rightarrow 0$ in a sector of each of the *q*-series of the top row of the matrix ${\textbf{J}}(q)$ is a linear combination of the $\Phi ^{(\sigma )}(h)$ series which includes the $\Phi ^{(\sigma _0)}(h)$ series.
- **A matrix-valued holomorphic quantum modular form.** In [GZ23] the first author and Don Zagier studied a matrix ${\textbf{J}}^\text {red}(q)$ of *q*-series with rows indexed by nontrivial flat connections, and conjectured that the corresponding value of the cocycle ${\textbf{J}}({\tilde{q}})^{-1} \Delta (\tau ) {\textbf{J}}(q)$^2^ at , which a priori is an analytic function on $\mathbb {C}\setminus \mathbb {R}$, actually extends to the cut plane $\mathbb {C}'$. A problem posed was to find an extension of the matrix ${\textbf{J}}^\text {red}(q)$ which includes the trivial flat connection. We do so in Sects. 2.2, 3.2 and 4.1 for the $\textbf{4}_1$ and $\textbf{5}_2$ knots.
- **An exact form of the Refined Quantum Modularity Conjecture.** In [GZ24] a Refined Quantum Modularity Conjecture was formulated. The conjecture was numerically motivated by a smoothed optimal summation of the divergent series $\Phi ^{\sigma )}(\tau )$, and the final result was a matrix-valued periodic function defined at the rational numbers. We conjecture that if we replace the smoothed optimal truncation by the median Borel resummation, all asymptotic statements in [GZ24] become exact equalities, valid for finite (and not necessarily large) range of the parameters.
- **An analytic extension of the Kashaev invariant and of the colored Jones polynomial.** A consequence of the above conjecture is an exact formula for the Kashaev invariant at rational points as a linear combinations of three smooth functions, multiplied by the top row of ${\textbf{J}}$.

#### Conjecture 1

For every knot *K* and every natural number *N* we have:

1 $$\begin{aligned} \langle K \rangle _N = \sum _{\sigma } c^K_\sigma N^{\delta _\sigma } s_{{\textrm{med}}}( \Phi ^{(K,\sigma )})(\tfrac{1}{N}) \end{aligned}$$

where $\delta _\sigma =3/2$ for $\sigma \ne \sigma _0$ and $\delta _{\sigma _0}=0$ (as in [GZ24, Eq. (3.7)]) and $(c^K_\sigma )$ is a vector of elements of the Habiro ring (tensor $\mathbb {Q}$) evaluated at $q=1$, with $c^K_{\sigma _1}=c^K_{\sigma _0}=1$.

The vector $(c_\sigma )$ for the $\textbf{4}_1$ knot appears in Sect. 4.2 of [GZ24] and also as the top row of the matrix of Eq. (92), and for the $\textbf{5}_2$ knot it appears in Section 4.3 as well as the top row of the matrix of Eq. (104) of ibid.

For the $\textbf{4}_1$ and the $\textbf{5}_2$ knots, we find numerically that $c^{\textbf{4}_1}_{\sigma _2}=0$, $c^{\textbf{5}_2}_{\sigma _2}=0$ and $c^{\textbf{5}_2}_{\sigma _3}=-2$ in complete agreement with the results of [GZ24]. A corollary of (1) is the Volume Conjecture $\langle K \rangle _N \sim N^{3/2} \Phi ^{(K,\sigma )}(\tfrac{1}{N})$ to all orders in 1/*N* as $N \rightarrow \infty$.

#### Conjecture 2

For every knot *K*, there is a neighborhood $U^K$ of 0 in the complex plane, such that for every natural number *N* and for $u \in U^K$, we have

2 $$\begin{aligned} J^K_N(e^{\frac{2\pi {\textrm{i}}}{N}+\frac{u}{N}})= \sum _{\sigma } \delta _\sigma (u,N) c^K_\sigma ({\tilde{x}}) s_{{\textrm{med}}}( \Phi ^{(K,\sigma )})(e^u;\tau ) \end{aligned}$$

where $\delta _{\sigma }(u,N)=\tau ^{-1/2} \frac{{\tilde{x}}^{1/2}-{\tilde{x}}^{-1/2}}{x^{1/2}-x^{-1/2}}$ for $\sigma \ne \sigma _0$ and $\delta _{\sigma _0}(x,\tau )=1$, where $x=e^u$, ${\tilde{x}}=e^{u/x}$, $\tau =\tfrac{u}{2\pi {\textrm{i}}N}+\tfrac{1}{N}$, and $c^K_\sigma ({\tilde{x}}) \in \mathbb {Q}[{\tilde{x}}^{\pm 1}]$ with $c^K_{\sigma _1}({\tilde{x}})=c^K_{\sigma _0}({\tilde{x}})=1$.

For the $\textbf{4}_1$ and the $\textbf{5}_2$ knots, we find numerically that $c^{\textbf{4}_1}_{\sigma _2}({\tilde{x}})=-\frac{{\tilde{x}}-{\tilde{x}}^{-1}}{2}$, $c^{\textbf{5}_2}_{\sigma _2}({\tilde{x}})=-\frac{{\tilde{x}}-{\tilde{x}}^{-1}}{2}$ and $c^{\textbf{5}_2}_{\sigma _3}({\tilde{x}})=-1-{\tilde{x}}$.

Since $\lim _{u\rightarrow 0} \delta _\sigma (N,u)=N^{\delta _s}$, the above conjecture specialises to Conjecture 1 when $u \rightarrow 0$. Note also that the above conjecture implies the Generalised Volume Conjecture when $u \not \in \pi {\textrm{i}}\mathbb {Q}$ is fixed and $N \rightarrow \infty$. Indeed, $\delta (N,u)$ is nonzero and $J^{K}_N(e^{(2\pi {\textrm{i}}+u)/N}) \sim \delta (N,u)\Phi ^{(\sigma _1)}(e^u;\tau )$. Note finally that the above conjecture explains the failure of exponential growth when *u* is a rational multiple of $2\pi {\textrm{i}}$, known for all knots from theorems 1.10 and 1.11 of [GL11], and theorem 5.3 of [Mur11] valid for the $\textbf{4}_1$ knot. Indeed, when $u=2\pi {\textrm{i}}r/s$ for integers *r* and *s* with *r*/*s* near zero, then $J^{K}_N(e^{(2\pi {\textrm{i}}+u)/N})$ is a periodic function of *N* (see [Hab02a]), and so is $\delta (N,u)$ since $e^{u/\tau }=e^{2\pi {\textrm{i}}N r/(r+s)}$. Moreover, $\delta (N,u)=0$ when *N* is a multiple of $r+s$ which explains why in that case the colored Jones polynomial does not grow exponentially.

- **An extension of the 3D-index.** Our completed matrix proposes a computable extension of the 3D-index in the sector of the trivial connection $\sigma _0$, whose mathematical or physical definition is yet-to-be given.

### Challenges

Our solution to the above problems brings a new challenge: namely, the new square matrix is actually a submatrix of a larger matrix ${\textbf{J}}(x,q)$, one with block triangular form which is a fundamental solution to the linear *q*-difference equation satisfied by the descendants of the colored Jones polynomials [GK23]. Already for the case of the $\textbf{5}_2$ knot, one obtains a $6 \times 6$ matrix instead of the original $3 \times 3$ matrix ${\textbf{J}}^\text {red}(x,q)$, or of the completed $4 \times 4$ matrix.

A second challenge is to interpret the integers appearing in the new Stokes constants associated to the trivial flat connection as BPS indices in the dual 3d super conformal field theory. Incorporating the trivial connection in the 3d/3d correspondence of [DGG14] is subtle, but we expect our explicit results to give hints on this problem.

We should point out that although a proof of resurgence of the asymptotic series $\Phi ^{(\sigma )}(h)$ is *still* missing, the current paper (as well as the prior ones [GGMn21, GGMn23]) provide a complete description of their resurgent structure (namely the location of the singularities and a calculation of the Stokes constants) with precise statements, complemented by extensive numerical computations (including a numerical computation of the Stokes constants). In addition, we provide proofs of the algebraic properties of the matrices of *q*-series and (*x*, *q*)-series.

### Illustration with the two simplest hyperbolic knots

We will illustrate our ideas by giving a detailed description of these matrices and of their algebraic, analytic and asymptotic properties for the case of the two simplest hyperbolic knots, the $\textbf{4}_1$ and the $\textbf{5}_2$ knots. Let us summarise our findings for the $\textbf{4}_1$ knot.

- We complete the $2 \times 2$ matrix ${\textbf{J}}^\text {red}(x,q)$ of (*x*, *q*)-series to the $3 \times 3$ matrix ${\textbf{J}}(x,q)$ by adding the trivial flat connection. Our completed matrix is a fundamental solution of a third order linear *q*-difference equation.
- A distinguished entry of ${\textbf{J}}(x,q)$ is the Gukov–Manolescu series.
- The matrix ${\textbf{J}}(x,q)$ determines explicitly (but conjecturally) the Stokes constants and hence the resurgence structure of the three perturbative formal power series.
- The matrix ${\textbf{J}}(x,q)$ conjecturally computes an extension of the 3D-index in a sector of the trivial flat connection.
- We complete the $2 \times 2$ matrix of descendant Andersen–Kashaev state-integrals to a $3 \times 3$ matrix by adding new state-integrals which are implicit in work of Kashaev and show their bilinear factorisation property.

As a second example, we present our results for the $\textbf{5}_2$ knot. In this case, we complete the $3 \times 3$ matrix ${\textbf{J}}^\text {red}(q)$ to a $4 \times 4$ one, and use it to describe explicitly the Stokes constants of the 4 asymptotic series in half of the complex plane, thus completing the resurgence question of those asymptotic series. However, the $\textbf{5}_2$ knot reveals a new puzzle: the $4 \times 4$ matrix is a block of a $6 \times 6$ matrix whose rows are a fundamental solution to a sixth order linear *q*-difference equation, namely the one satisfied by the descendant colored Jones polynomial of the $\textbf{5}_2$ knot [GK23, Eq. (14)]. Although the homogeneous linear *q*-difference equation for the colored Jones polynomial is fourth order, the one for the descendant colored Jones polynomial is sixth order, and both equations are knot invariants. In the case of the $\textbf{5}_2$ knot, the extra $2 \times 2$ block is a matrix of modular functions, in fact of the famous Rogers–Ramanujan modular *q*-hypergeometric series. We do not understand the labeling of the two excess rows and columns (e.g., in terms of $\textrm{SL}_2(\mathbb {C})$-flat connections). Since the formulas for the $6 \times 6$ matrix appear rather complicated, we will not give the *x*-deformation here, and postpone to a future publication a systematic definition of the matrix of (*x*, *q*)-series for all knots.

We should point out that the definition of the top row of the $3 \times 3$ matrices for the $\textbf{4}_1$ knot, and the $6 \times 6$ matrix for the $\textbf{5}_2$ knot, as well as an extension of the above results to the case of closed hyperbolic 3-manifolds have been taken from the thesis of the last author [Whe23].

## The $\textbf{4}_1$ knot

### A $2 \times 2$ matrix of *q*-series

In this section we recall in detail what is known about the resurgence of the two asymptotic series of the $\textbf{4}_1$ knot, labeled by the geometric and the complex-conjugate flat connections. As explained in the introduction, the answer is determined by a $2 \times 2$ matrix of *q*-series which was discovered in a long story and in several stages in a series of papers [GZ23, GK17, GGMn21, GGMn23]. A detailed description of the numerical discoveries and coincidences is given in [GZ23] and will not be repeated here. In that paper, the following pair of *q*-series $G^{(j)}(q)$ for $j=0,1$ was introduced and studied by the first author and Zagier [GZ23]

3 $$\begin{aligned} \begin{aligned} G^{(0)}(q)&=\sum _{n\ge 0}(-1)^{n}\frac{q^{n(n+1)/2}}{(q;q)_{n}^{2}} \\ G^{(1)}(q)&=\sum _{n\ge 0}\left( n+\frac{1}{2}-2E_{1}^{(n)}(q)\right) (-1)^{n} \frac{q^{n(n+1)/2}}{(q;q)_{n}^{2}} \end{aligned} \end{aligned}$$

where

4 $$\begin{aligned} E_{k}^{(n)}(q) = \sum _{s=1}^{\infty }s^{k-1}\frac{q^{s(n+1)}}{1-q^{s}} . \end{aligned}$$

These series were found to be connected to the $\textbf{4}_1$ knot in at least two ways, discussed in detail in [GZ23]. On the one hand, they express bilinearly the Andersen-Kashaev state-integral [GK17] and the total 3D-index of Dimofte-Gaiotto-Gukov [DGG13]. On the other hand, their radial asymptotics as $q=e^{2\pi {\textrm{i}}\tau } \rightarrow 1$ (where $\tau$ is in a ray in the upper half-plane) is a linear combination of the two asymptotic series $\Phi ^{(\sigma _1)}(\tau )$ and $\Phi ^{(\sigma _2)}(\tau )$ of the Kashaev invariant, where $\sigma _1$ is the geometric representation of the fundamental group of the knot complement and $\sigma _2$ is the complex conjugate. The resurgence of the factorially divergent asymptotic series $\Phi ^{(\sigma _1)}(\tau )$ and $\Phi ^{(\sigma _2)}(\tau )$, including a complete description of the Stokes automorphism and the Borel resummation was given by the first three authors in [GGMn21]. Surprisingly, the Stokes matrices were expressed bilinearly in terms of a $2 \times 2$ matrix of explicit descendant *q*-series whose definition we now give. Consider the linear *q*-difference equation

5 $$\begin{aligned} f_m(q) + (q^{m+1}-2) f_{m+1}(q) + f_{m+2}(q) = 0 \qquad (m \in \mathbb {Z}) . \end{aligned}$$

In [GGMn21] it was shown that it has a basis of solutions $G^{(j)}_m(q)$ for $j=1,2$ given by^3^

6 $$\begin{aligned} \begin{aligned} G_{m}^{(0)}(q)&=\sum _{n\ge 0}(-1)^{n}\frac{q^{n(n+1)/2}}{(q;q)_{n}^{2}}q^{mn}\\ G_{m}^{(1)}(q)&=\sum _{n\ge 0}\left( n+m+\frac{1}{2}-2E_{1}^{(n)}(q)\right) (-1)^{n} \frac{q^{n(n+1)/2}}{(q;q)_{n}^{2}}q^{mn} \end{aligned} \end{aligned}$$

where $E_{k}^{(n)}(q)$ are as in Equation (4). Note that $G_0^{(j)}(q)=G^{(j)}(q)$, and that $G^{(j)}_m(q) \in \mathbb {Z}((q))$ are Laurent series in *q* (with finitely many negative powers of *q*), meromorphic on $|q|<1$ with only possible pole at $q=0$. We will extend them to analytic functions on $|q| \ne 1$ by

7 $$\begin{aligned} G_m^{(j)}(q^{-1}) = (-1)^{i}G^{(j)}_{-m}(q),\qquad j=0,1. \end{aligned}$$

The $2 \times 2$ matrix is given by  where

8 $$\begin{aligned} {\textbf{J}}^\text {red}_{m}(q) = \begin{pmatrix} G_{m}^{(1)}(q) & G_{m+1}^{(1)}(q)\\ G_{m}^{(0)}(q) & G_{m+1}^{(0)}(q)\\ \end{pmatrix} \end{aligned}$$

coincides with the transpose of the matrix $W_m(q)$ of [GGMn23, Eq. (48)] after interchanging of the two rows. A complete description of the resurgent structure of the series $\Phi ^{(\sigma _j)}(\tau )$ for $j=0,1$, of their Borel resummation and their expression in terms of a $2 \times 2$ matrix of state-integrals (with one distinguished entry being the Andersen–Kashaev state-integral [AK14]) was given in [GGMn21, GGMn23].

### A $3 \times 3$ matrix of *q*-series

In this section we define the promised $3 \times 3$ matrix of *q*-series ${\textbf{J}}^\text {red}_{m}(q)$ and give several algebraic properties thereof. In his thesis [Whe23], the fourth author introduced the series $G^{(2)}(q)$

9 $$\begin{aligned} G^{(2)}(q) = \sum _{n\ge 0}\left( \frac{1}{2}\left( n+\frac{1}{2}-2E_{1}^{(n)}(q)\right) ^2 -E_{2}^{(n)}(q) -\frac{1}{24}E_{2}(q)\right) (-1)^{n}\frac{q^{n(n+1)/2}}{(q;q)_{n}^{2}}\nonumber \\ \end{aligned}$$

which is the coefficient of $\varepsilon ^2$ in the $\varepsilon$-deformed *q*-series

10 $$\begin{aligned} G(q,\varepsilon )= e^{-\varepsilon ^{2}\frac{E_{2}(q)}{24}} \sum _{n=0}^{\infty }(-1)^{n}\frac{q^{n(n+1)/2}e^{(n+1/2)\varepsilon }}{(qe^{\varepsilon };q)_{n}^{2}} = \sum _{k=0}^{\infty }G^{(k)}(q)\varepsilon ^{k} \end{aligned}$$

which appears in [GZ23]. Here, $E_2(q)=1-24E_2^{(0)}(q)$. Adding the descendant variable $m \in \mathbb {Z}$, leads to the *q*-series

11 $$\begin{aligned} G_{m}^{(2)}(q) = \sum _{n\ge 0}\left( \frac{1}{2}\left( n+m+\frac{1}{2}-2E_{1}^{(n)}(q)\right) ^2 -E_{2}^{(n)}(q) -\frac{1}{24}E_{2}(q)\right) (-1)^{n}\frac{q^{n(n+1)/2}}{(q;q)_{n}^{2}}q^{mn}\nonumber \\ \end{aligned}$$

As in the case of $G_{m}^{(j)}(q)$ for $j=0,1$, it is a meromorphic function on $|q|<1$ with only possible pole at $q=0$, and extends to an analytic function on $|q|>1$ satisfying (7) with $j=2$.

The sequence $G_{m}^{(2)}(q)$ is a solution of the inhomogenous equation obtained by replacing the right hand side of (5) by 1. This follows easily by using creative telescoping of the theory of *q*-holonomic functions implemented by Koutschan [Kou10].

We can assemble the three sequences of *q*-series into a matrix

12 $$\begin{aligned} {\textbf{J}}_{m}(q) = \begin{pmatrix} 1 & G_{m}^{(2)}(q) & G_{m+1}^{(2)}(q)\\ 0 & G_{m}^{(1)}(q) & G_{m+1}^{(1)}(q)\\ 0 & G_{m}^{(0)}(q) & G_{m+1}^{(0)}(q)\\ \end{pmatrix} \end{aligned}$$

whose bottom-right $2 \times 2$ matrix is ${\textbf{J}}^\text {red}_{m}(q)$. The next theorem summarises the properties of ${\textbf{J}}_{m}(q)$.

#### Theorem 3

The matrix ${\textbf{J}}_{m}(q)$ is a fundamental solution to the linear *q*-difference equation

13 $$\begin{aligned} {\textbf{J}}_{m+1}(q) = {\textbf{J}}_{m}(q) A(q^m,q), \qquad A(q^m,q)= \begin{pmatrix} 1 & 0 & 1\\ 0 & 0 & -1\\ 0 & 1 & 2-q^{m+1}\\ \end{pmatrix} , \end{aligned}$$

has $\det ({\textbf{J}}_m(q))=-1$ and satisfies the analytic extension

14 $$\begin{aligned} {\textbf{J}}_m(q^{-1}) = \begin{pmatrix} 1& 0& 0\\ 0& -1& 0\\ 0& 0& 1 \end{pmatrix} {\textbf{J}}_{-m-1}(q) \begin{pmatrix} 1& 0& 0\\ 0& 0& 1\\ 0& 1& 0 \end{pmatrix} . \end{aligned}$$

#### Proof

Equation (13) follows from the fact the last two rows of ${\textbf{J}}_m(q)$ are solutions of the *q*-difference equation (5) and the first is a solution of the corresponding inhomogenous equation. Moreover, the block form of ${\textbf{J}}_m(q)$ implies that $\det ({\textbf{J}}_m(q))=\det ({\textbf{J}}^\text {red}_m(q))=-1$ where the last equality follows from [GGMn21, Eq. (14)]. Equation (14) follows from the fact that all three sequences of *q*-series satisfy (7). $\square$

We now give the inverse matrix of ${\textbf{J}}_m(q)$ in terms of Appell-Lerch like sums. The latter appear curiously in the mock modular forms and the meromorphic Jacobi forms of Zwegers [Zwe01], and in [DMZ].

#### Theorem 4

We have

15 $$\begin{aligned} {\textbf{J}}_{m}(q)^{-1} = \begin{pmatrix} 1 & L_{m}^{(0)}(q) & -L_{m}^{(1)}(q) \\ 0 & -G_{m+1}^{(0)}(q) & G_{m+1}^{(1)}(q) \\ 0 & G_{m}^{(0)}(q) & -G_{m}^{(1)}(q) \end{pmatrix} \end{aligned}$$

for the *q*-series $L_m^{(j)}(q)$ ($j=0,1$) defined by

16 $$\begin{aligned} \begin{aligned} L^{(0)}_{m}(q)&= G_{m+1}^{(0)}(q)G_{m}^{(2)}(q)-G_{m}^{(0)}(q)G_{m+1}^{(2)}(q)\\ L_{m}^{(1)}(q)&= G_{m+1}^{(1)}(q)G_{m}^{(2)}(q)-G_{m}^{(1)}(q)G^{(2)}_{m+1}(q) . \end{aligned} \end{aligned}$$

The *q*-series $L_m^{(j)}(q)$ are expressed in terms of Appell-Lerch type sums:

17 $$\begin{aligned} L^{(0)}_{m}(q)= &  2E_{1}^{(0)}(q)-1-m+ \sum _{n=1}^{\infty }(-1)^{n}\frac{q^{n(n+1)/2}}{(q;q)_{n}^{2}}\frac{q^{mn+n}}{1-q^{n}}\nonumber \\ L_{m}^{(1)}(q)= &  -\frac{3}{8}-2E_{1}^{(0)}(q)^{2}+2E_{1}^{(0)}(q)-E_{2}^{(0)}(q) -\frac{1}{24}E_{2}(q)+2mE_{1}^{(0)}(q)-m\nonumber \\ &  -\frac{m^{2}}{2} +\sum _{n=1}^{\infty }(-1)^{n}\frac{q^{n(n+1)/2}}{(q)_{n}^{2}} \frac{q^{mn+n}}{1-q^{n}}\left( n+m+\frac{1}{2}-2E^{(n)}_{1}(q)+\frac{1}{1-q^{n}}\right) .\nonumber \\ \end{aligned}$$

#### Proof

Since ${\textbf{J}}^\text {red}_{m}(q)$ is a $2 \times 2$ matrix with determinant $-1$, it follows that the inverse matrix ${\textbf{J}}_{m}(q)^{-1}$ is given by (15) for the *q*-series $L_m^{(j)}(q)$ ($j=0,1$) given by (16).

Observe that $A(q^m,q)$ has first column $(1,0,0)^t$, first row (1, 0, 1), and the remaining part is a companion matrix. It follows that its inverse matrix has first column $(1,0,0)^t$ and first row (1, 1, 0). This, together with (13) implies that

18 $$\begin{aligned} {\textbf{J}}_{m+1}(q)^{-1} = A(q^m,q)^{-1} {\textbf{J}}_{m}(q)^{-1} = \begin{pmatrix} 1 & 1 & 0\\ 0 & 2-q^{m+1} & 1\\ 0 & -1 & 0\\ \end{pmatrix} {\textbf{J}}_{m}(q)^{-1} . \end{aligned}$$

It follows that $L_m^{(j)}(q)$ satisfy the first order inhomogeneous linear *q*-difference equation

19 $$\begin{aligned} L^{(j)}_{m-1}(q)-L^{(j)}_{m}(q) = G_{m}^{(j)}(q) \qquad (j=0,1) . \end{aligned}$$

Let ${\mathcal {L}}^{(0)}_m(q)$ denote the right hand side of the top Equation (17). Then we have

$$\begin{aligned} {\mathcal {L}}^{(0)}_{m-1}(q)-{\mathcal {L}}^{(0)}_{m}(q) = 1+\sum _{n=1}^{\infty }(-1)^{n}\frac{q^{n(n+1)/2}}{(q)_{n}^{2}}\frac{q^{mn}-q^{mn+n}}{1-q^{n}} = G_{m}^{(0)}(q). \end{aligned}$$

Therefore ${\mathcal {L}}^{(0)}_{m}(q)-L_{m}^{(0)}(q)$ is independent of *m*. Moreover, $\lim _{m \rightarrow \infty } {\mathcal {L}}^{(0)}_{m}(q)-L_{m}^{(0)}(q)=0$. The top part of Equation (17) follows.

Likewise, let ${\mathcal {L}}^{(1)}_m(q)$ denote the right hand side of the bottom part of Equation (17). Then we have

$$\begin{aligned} {\mathcal {L}}^{(1)}_{m-1}(q)-{\mathcal {L}}^{(1)}_{m}(q)&= \sum _{n=1}^{\infty }(-1)^{n}\frac{q^{n(n+1)/2}}{(q)_{n}^{2}} \frac{q^{mn}-q^{mn+n}}{1-q^{n}}\\&\quad \left( n+m+\frac{1}{2} -2E^{(n)}_{1}(q)+\frac{1}{1-q^{n}}\right) \\&\quad -\sum _{n=1}^{\infty }(-1)^{n}\frac{q^{n(n+1)/2}}{(q)_{n}^{2}} \frac{q^{mn}}{1-q^{n}}+m+\frac{1}{2}-2E^{(0)}_{1}(q)\\&= G^{(1)}_{m}(q) \,. \end{aligned}$$

Therefore ${\mathcal {L}}^{(1)}_{m}(q)-L^{(1)}_{m}(q)$ is independent of *m*. Moreover, $\lim _{m \rightarrow \infty } {\mathcal {L}}^{(1)}_{m}(q)-L_{m}^{(1)}(q)=0$. Equation (17) follows. $\square$

### The $\Phi ^{(\sigma _0)}(\tau )$ asymptotic series

The $\textbf{4}_1$ knot has three asymptotic series $\Phi ^{(\sigma _j)}(\tau )$ for $j=0,1,2$ corresponding to the trivial flat connection $\sigma _0$, the geometric flat connection $\sigma _1$ and its complex conjugate $\sigma _2$. The asymptotic series $\Phi ^{(\sigma _j)}(\tau )$ for $j=1,2$ are defined in terms of perturbation theory of a state-integral [DG13] and can be computed via formal Gaussian integration in a way that was explained in detail in [GGMn21, GZ24] and will not be repeated here. They have the form

20 $$\begin{aligned} \Phi ^{(\sigma _j)}(\tau ) = e^{\tfrac{V(\sigma _j)}{2\pi {\textrm{i}}\tau }} \varphi ^{(\sigma _j)}(\tau ),\quad j=1,2, \end{aligned}$$

where

21 $$\begin{aligned} V(\sigma _1) = -V(\sigma _2) = {\textrm{i}}\text {Vol}(\textbf{4}_1) = {\textrm{i}}2\text {Im} \textrm{Li}_2({\textrm{e}}^{{\textrm{i}}\pi /3}) = {\textrm{i}}2.029883\ldots , \end{aligned}$$

with $\text {Vol}(\textbf{4}_1)$ being the hyperbolic volume of $S^3\backslash \textbf{4}_1$, and $\varphi ^{(\sigma _1)}(\tfrac{h}{2\pi {\textrm{i}}})$ with $h=2\pi {\textrm{i}}\tau$ is a power series with algebraic coefficients with first few terms

22 $$\begin{aligned} \varphi ^{(\sigma _1)}(\tfrac{h}{2\pi {\textrm{i}}}) = 3^{-1/4}\left( 1+\frac{11 h}{72\sqrt{-3}} +\frac{697 h^2}{2(72\sqrt{-3})^2}+\ldots \right) \end{aligned}$$

(a total of 280 terms have been computed), while $\varphi ^{(\sigma _2)}(\tau ) = {\textrm{i}}\varphi ^{(\sigma _1)}(-\tau )$.

We now discuss the new series $\varphi ^{(\sigma _0)}(\tau ) \in \mathbb {Q}[[\tau ]]$ corresponding to the zero volume $V(\sigma _0)=0$ trivial flat connection. This series can be defined and computed (for any knot) using either the colored Jones polynomial or the Kashaev invariant. Let us recall how this works.

Let $J_n(q) \in \mathbb {Z}[q^{\pm 1}]$ denotes the Jones polynomial colored by the *n*-dimensional irreducible representation of ${{\mathfrak {s}}}{{\mathfrak {l}}}_2$, and normalised to 1 at the unknot. Setting $q=e^h$, one obtains a power series in *h*, whose coefficient of $h^k$ is a polynomial in *n* of degree at most *k*. In other words, we have

23 $$\begin{aligned} J_n(e^h) = \sum _{i=0}^\infty \sum _{j=0}^i a_{i,j} n^j h^i \in \mathbb {Q}[[n,h]] \end{aligned}$$

where $a_{i,j}$ depends on the knot and, as the knot varies, defines a Vassiliev invariant of type (i.e., degree) *i* [BN95]. Then, the perturbative series $\varphi ^{(\sigma _0)}(\tau )$ is given by

24 $$\begin{aligned} \varphi ^{(\sigma _0)}(\tfrac{h}{2\pi {\textrm{i}}}) = \sum _{i=0}^\infty a_{i,0} h^i . \end{aligned}$$

With this definition, to compute the coefficient of $\tau ^k$ in $\varphi ^{(\sigma _0)}(\tau )$, one needs to compute the first *k* colored Jones polynomials $J_n(e^h)$ for $k =1, \dots , n$ up to $O(h^{k+1})$, polynomially interpolate and extract the coefficient $a_{k,0}$. An efficient computation of the colored Jones polynomial is possible if one knows a recursion relation with respect to *n* (such a relation always exists [GL05]) together with some initial conditions. This gives a polynomial time algorithm to compute $J_n(e^h)+O(h^{k+1})$.

An alternative method is the so-called loop expansion of the colored Jones polynomial

25 $$\begin{aligned} J_n(e^h) = \sum _{\ell =0}^\infty \frac{P_\ell (x)}{\Delta (x)^{2\ell +1}} h^\ell \in \mathbb {Z}[x^{\pm 1}, \Delta (x)^{-1}][[h]] \end{aligned}$$

where $x=q^n=e^{n h}$ and $\Delta (x) \in \mathbb {Z}[x^{\pm 1}]$ is the Alexander polynomial of the knot. This expansion was introduced by Rozansky [Roz98] (see also Kricker [Kri, GK04]) and it is related to the Vassiliev power series expansion (23) by

26 $$\begin{aligned} \sum _{k=0}^\infty a_{\ell +k,k} h^k= \frac{P_\ell (e^h)}{\Delta (e^h)^{2\ell +1}} . \end{aligned}$$

Then the perturbative series $\varphi ^{(\sigma _0)}(\tau )$ is given in terms of the loop expansion by

27 $$\begin{aligned} \varphi ^{(\sigma _0)}(\tfrac{h}{2\pi {\textrm{i}}}) = \sum _{\ell =0}^\infty P_\ell (1) h^\ell \end{aligned}$$

as follows from the above equations together with the fact that $\Delta (1)=1$.

A third method uses a theorem of Habiro [Hab02b, Hab08] which lifts the Kashaev invariant of a knot to an element of the Habiro ring $\widehat{\mathbb {Z}[q]} = \varprojlim \;\mathbb {Z}[q]/((q;q)_n)$. There is a canonical ring homomorphism $\widehat{\mathbb {Z}[q]} \rightarrow \mathbb {Z}[[h]]$ defined by $q \mapsto e^h$, which sends $(q;q)_n$ to $(-1)^n h^n + O(h^{n+1})$ and the image of the lifted element of the Habiro ring under this homomorphism equals to the series $\varphi ^{(\sigma _0)}(h)$. For the case of the $\textbf{4}_1$ knot, the corresponding element of the Habiro ring is given by

28 $$\begin{aligned} \sum _{n=0}^\infty (q;q)_n (q^{-1};q^{-1})_n \end{aligned}$$

and its expansion when $q=e^h$ gives the power series with first few terms

29 $$\begin{aligned} \varphi ^{(\sigma _0)} (\tfrac{h}{2\pi {\textrm{i}}})= 1-h^2+\frac{47}{12}h^4+\ldots . \end{aligned}$$

We end this section with a comment. Going back to the case of a general knot, it was shown in [GK23] that the colored Jones polynomial is equivalent (in the sense of knot invariants) to a descendant sequence of colored Jones polynomials and of Kashaev invariants (indexed by the integers) which is *q*-holonomic. These descendant Kashaev invariants play a key role in extending matrices of periodic functions whose rows and columns are indexed by nonrtivial flat connections to a matrix that includes the trivial flat connection. This is explained in detail in [GZ24].

### Borel resummation and Stokes constants

In this section we discuss the asymptotic expansion as $q = {\textrm{e}}^{2\pi {\textrm{i}}\tau }\rightarrow 1$ of the vector *G*(*q*) of *q*-series and relate it to the vector $\Phi (\tau )$ of the asymptotic series, where

30 $$\begin{aligned} G(q) = \begin{pmatrix} G^{(2)}(q) \\ G^{(1)}(q) \\ G^{(0)}(q) \end{pmatrix}, \qquad \Phi (\tau ) = \begin{pmatrix} \Phi ^{(\sigma _0)}(\tau )\\ \Phi ^{(\sigma _1)}(\tau )\\ \Phi ^{(\sigma _2)}(\tau ) \end{pmatrix} \end{aligned}$$

with $G^{(0)}(q), G^{(1)}(q)$ given in (3), and the additional series $G^{(2)}(q)$ given in (9).

The three power series $\Phi ^{(\sigma _j)}(\tau )$, $j=0,1,2$ can be resummed by Borel resummation. On the other hand, according to the resurgence theory, the value of the Borel resummation of an asymptotic power series depends crucially on the argument of the expansion variable. If the Borel transform of the power series has a singular point located at $\iota$, the values of the Borel resummation of the power series whose expansion variable has an argument slightly greater and less than the angle $\theta = \arg \iota$ differ by an exponentially small quantity, called the Stokes discontinuity. Usually the difference is identical with the Borel resummation of another power series in the theory, a phenomenon called the Stokes automorphism.

**Fig. 1:**
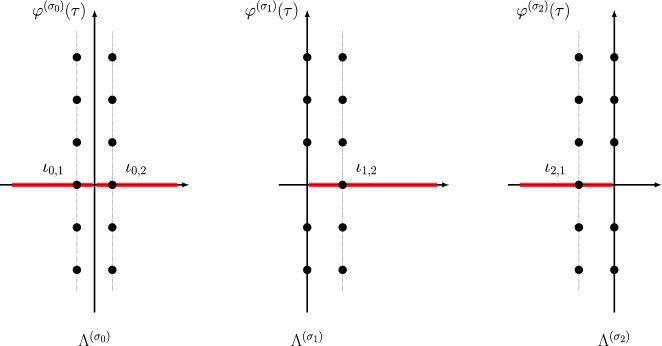
Singularities of the Borel transforms of $\varphi ^{(\sigma _j)}(\tau )$ for $j=0,1,2$ of the knot $\textbf{4}_1$. Red lines are (some) Stokes rays

In the case of the power series $\Phi ^{(\sigma _j)}(\tau )$, $j=0,1,2$, the singularities of the Borel transforms of $\Phi ^{(\sigma _j)}(\tau )$, $j=1,2$ were already studied in [GGMn21, GGMn23], and they are located at

31 $$\begin{aligned} \Lambda ^{(\sigma _j)} = \{ \iota _{j,i} +2\pi {\textrm{i}}k\;|\; i=1,2,i\ne j,\, k\in {\mathbb {Z}}\}\cup \{2\pi {\textrm{i}}k\;|\; k\in {\mathbb {Z}}_{\ne 0}\},\quad j=1,2, \end{aligned}$$

as shown in the middle and the right panels of Fig. 1, while the singularities of the Borel transform of $\Phi ^{(\sigma _0)}(\tau )$ are located at (see also [Gar08a, Conj. 4])

32 $$\begin{aligned} \Lambda ^{(\sigma _0)} = \{\iota _{0,i} +2\pi {\textrm{i}}k\;|\; i=1,2,\,k\in {\mathbb {Z}}\}, \end{aligned}$$

as shown in the left panel of Fig. 1, where

33 $$\begin{aligned} \iota _{j,i} = \frac{V(\sigma _j) - V(\sigma _i)}{2\pi {\textrm{i}}},\quad i,j=0,1,2. \end{aligned}$$

**Fig. 2:**
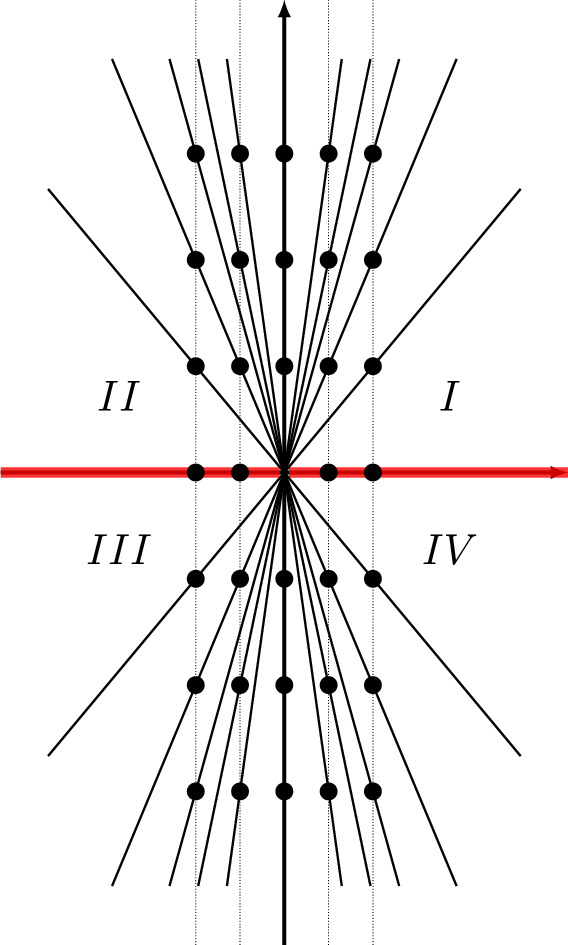
Stokes rays and cones in the $\tau$-plane for the 3-vector $\Phi (\tau )$ of asymptotic series of the knot $\textbf{4}_1$. Red lines are (some) Stokes rays

All the rays $\rho _\theta$ (Stokes rays) passing through the singularities in the union

34 $$\begin{aligned} \Lambda = \cup _{j=0,1,2} \Lambda ^{(\sigma _j)}, \end{aligned}$$

form a peacock pattern, cf. Fig. 2, and they divide the complex plane of Borel transform into infinitely many cones. The Borel resummation of the vector $\Phi (\tau )$ is only well-defined within one of these cones.

Recall that the Borel transform ${\widehat{\varphi }}(\zeta )$ of a Gevrey-1 power series $\varphi (\tau )$

35 $$\begin{aligned} \varphi (\tau ) = \sum _{n=0}^\infty a_n\tau ^n,\quad a_n = O(C^n n!), \end{aligned}$$

is defined by

36 $$\begin{aligned} {\widehat{\varphi }}(\zeta ) =\sum _{n=0}^\infty \frac{a_n}{n!}\zeta ^n. \end{aligned}$$

If it analytically continues to an $L^1$-analytic function along the ray $\rho _\theta :={\textrm{e}}^{{\textrm{i}}\theta }{\mathbb {R}}_+$ where $\theta = \arg \tau$, we define the Borel resummation by the Laplace integral

37 $$\begin{aligned} s_\theta (\varphi )(\tau ) = \int _0^\infty {\widehat{\varphi }}(\tau \zeta ) {\textrm{e}}^{-\zeta }{\textrm{d}}\zeta = \frac{1}{\tau }\int _{\rho _\theta } {\widehat{\varphi }}(\zeta ){\textrm{e}}^{-\zeta /\tau }{\textrm{d}}\zeta . \end{aligned}$$

The Borel resummation of the trans-series $\Phi (\tau ) = {\textrm{e}}^{\frac{V}{2\pi {\textrm{i}}\tau }}\varphi (\tau )$ is defined to be

38 $$\begin{aligned} s_\theta (\Phi )(\tau ) = {\textrm{e}}^{\frac{V}{2\pi {\textrm{i}}\tau }}s_\theta (\varphi )(\tau ). \end{aligned}$$

In the following we will also use the notation $s_R(\Phi )(\tau )$ when the argument of $\tau$ is in the cone *R* and it is a continuous function of $\tau$.

Coming back to the vector of *q*-series *G*(*q*), we find that the asymptotic expansion of *G*(*q*) when $q=e^{2\pi {\textrm{i}}\tau }$ and $\tau \rightarrow 0$ in a cone *R* can be expressed in terms of $\Phi (\tau )$. Moreover, this asymptotic expansion can be lifted to an exact identity between *q*-series $G^{(j)}(q)$ and linear combinations of Borel resummation of $\Phi ^{(\sigma _j)}(\tau )$ multiplied by power series in ${\tilde{q}}= {\textrm{e}}^{-2\pi {\textrm{i}}\tau ^{-1}}$ (thought of as exponentially small corrections) with integer coefficients. This is the content of the following conjecture.

#### Conjecture 5

For every cone $R \subset \mathbb {C}\setminus \Lambda$ and every $\tau \in R$, we have

39 $$\begin{aligned} \Delta '(\tau ) G(q) = M_R({\tilde{q}}) \Delta (\tau ) s_R(\Phi )(\tau ), \end{aligned}$$

where

40 $$\begin{aligned} \Delta '(\tau ) = \textrm{diag}(\tau ^{3/2},\tau ^{1/2},\tau ^{-1/2}),\quad \Delta (\tau ) = \textrm{diag}(\tau ^{3/2},1,1), \end{aligned}$$

and $M_R({\tilde{q}})$ is a $3\times 3$ matrix of ${\tilde{q}}$ (resp., ${\tilde{q}}^{-1}$)-series if $\textrm{Im}\tau >0$ (resp., $\textrm{Im}\tau <0$) with integer coefficients that depend on *R*.

As in [GGMn21, GGMn23], we pick out in particular four of these cones, located slightly above and below the positive or the negative real axis, labeled in clockwise direction by *I*, *II*, *III*, *IV* as indicated in Fig. 2. We work out the exact matrices $M_R({\tilde{q}})$ in these four cones.

#### Conjecture 6

Equation (39) holds in the cones $R=I,II,III,IV$ where the matrices $M_R({\tilde{q}})$ are given in terms of ${\textbf{J}}_{-1}({\tilde{q}})$ as follows

41a $$\begin{aligned} M_I({\tilde{q}})&= {\textbf{J}}_{-1}({\tilde{q}}) \begin{pmatrix} 1 & 0 & 0\\ 0 & 0 & -1\\ 0 & 1 & -1 \end{pmatrix}, \quad |{\tilde{q}}|<1, \end{aligned}$$

41b $$\begin{aligned} M_{II}({\tilde{q}})&= \begin{pmatrix} 1 & 0 & 0\\ 0 & -1& 0\\ 0 & 0 & 1 \end{pmatrix} {\textbf{J}}_{-1}({\tilde{q}}) \begin{pmatrix} 1& 0& 0\\ 0& 1& 0\\ 0& 1& -1 \end{pmatrix}, \quad |{\tilde{q}}|<1, \end{aligned}$$

41c $$\begin{aligned} M_{III}({\tilde{q}})&= \begin{pmatrix} 1 & 0 & 0\\ 0 & -1& 0\\ 0 & 0 & 1 \end{pmatrix} {\textbf{J}}_{-1}({\tilde{q}}) \begin{pmatrix} 1& 1& 0\\ 0& -1& 0\\ 0& 2& 1 \end{pmatrix}, \quad |{\tilde{q}}|>1, \end{aligned}$$

41d $$\begin{aligned} M_{IV}({\tilde{q}})&= {\textbf{J}}_{-1}({\tilde{q}}) \begin{pmatrix} 1& 0& 1\\ 0& 0& -1\\ 0& 1& 2 \end{pmatrix}, \quad |{\tilde{q}}| >1. \end{aligned}$$

We now discuss the Stokes automorphism. To any singularity in the Borel plane located at $\iota _{i,j}^{(k)}:= \iota _{i,j}+2\pi {\textrm{i}}k$, we can associate a local Stokes matrix

42 $$\begin{aligned} \mathfrak {S}_{\iota _{i,j}^{(k)}}({\tilde{q}}) = I + \mathcal {S}_{i,j}^{(k)}{\tilde{q}}^k E_{i,j},\quad \mathcal {S}_{i,j}^{(k)}\in {\mathbb {Z}}, \end{aligned}$$

where $E_{i,j}$ is the elementary matrix with (*i*, *j*)-entry 1 ($i,j=0,1,2$) and all other entries zero, and $\mathcal {S}_{i,j}^{(k)}$ is the Stokes constant. Let us assume the *locality* condition that no two Borel singularities share the same argument, or if there are, their Stokes matrices commute. This is indeed the case in our example. Then for any ray of angle $\theta$, the Borel resummations of $\Phi (\tau )$ with $\tau$ whose argument is raised slight above ($\theta _+$) or lowered sightly below ($\theta _-$) $\theta$ are related by the following formula of Stokes automorphism

43 $$\begin{aligned} \Delta (\tau ) s_{\theta _+}(\Phi )(\tau ) = \mathfrak {S}_{\theta }({\tilde{q}}) \Delta (\tau ) s_{\theta _-}(\Phi )(\tau ),\quad \mathfrak {S}_\theta ({\tilde{q}}) = \prod _{\arg \iota =\theta }\mathfrak {S}_\iota ({\tilde{q}}). \end{aligned}$$

Because of the locality condition, we don’t have to worry about the order of the product of local Stokes matrices.

More generally, consider two rays $\rho _{\theta ^+}$ and $\rho _{\theta ^-}$ whose arguments satsify $0<\theta ^+-\theta ^-\le \pi$, we define the global Stokes automorphism

44 $$\begin{aligned} \Delta (\tau )s_{\theta ^+}(\Phi )(\tau ) = \mathfrak {S}_{\theta ^-\rightarrow \theta ^+}({\tilde{q}}) \Delta (\tau )s_{\theta ^-}(\Phi )(\tau ), \end{aligned}$$

where both sides are analytically continued smoothly to the same value of $\tau$. The global Stokes matrix $\mathfrak {S}_{\theta ^-\rightarrow \theta ^+}({\tilde{q}})$ satisfies the factorisation property [GGMn21, GGMn23]

45 $$\begin{aligned} \mathfrak {S}_{\theta ^-\rightarrow \theta ^+}({\tilde{q}}) = \prod _{\theta ^-<\theta <\theta ^+}^{\leftarrow } \mathfrak {S}_{\theta }({\tilde{q}}), \end{aligned}$$

where the ordered product is taken over all the local Stokes matrices whose arguments are sandwiched between $\theta ^-,\theta ^+$ and they are ordered with rising arguments from right to left.

Given (39) with explicit values of $M_R({\tilde{q}})$ for $R=I,II,III,IV$, in general we can calculate the global Stokes matrix via

46 $$\begin{aligned} \mathfrak {S}_{R\rightarrow R'}({\tilde{q}}) = M_{R'}({\tilde{q}})^{-1}\cdot M_{R}({\tilde{q}}). \end{aligned}$$

Here in the subscript of the global Stokes matrix on the left hand side, *R* stands for any ray in the cone. For instance, we find that the global Stokes matrix from cone *I* anti-clockwise to cone *II* is

47 $$\begin{aligned} \mathfrak {S}_{I\rightarrow II}({\tilde{q}}) = \begin{pmatrix} 1& 0& 0\\ 0& 1& 0\\ 0& 1& -1 \end{pmatrix} {\textbf{J}}_{-1}({\tilde{q}})^{-1} \begin{pmatrix} 1& 0& 0\\ 0& -1& 0\\ 0& 0& 1 \end{pmatrix} {\textbf{J}}_{-1}({\tilde{q}}) \begin{pmatrix} 1& 0& 0\\ 0& 0& -1\\ 0& 1& -1 \end{pmatrix},\quad |{\tilde{q}}|<1.\nonumber \\ \end{aligned}$$

This Stokes matrix has the block upper triangular form

48 $$\begin{aligned} \begin{pmatrix} 1& *& *\\ 0& *& *\\ 0& *& * \end{pmatrix}. \end{aligned}$$

Let us note that this form implies that $\Phi ^{(\sigma _j)}(\tau )$ ($j=1,2$) form a closed subset under Stokes automorphisms (this was called in [GMn] a “minimal resurgent structure”). They are controled by the $2\times 2$ submatrix of $\mathfrak {S}_{I\rightarrow II}({\tilde{q}})$ in the bottom right and one can verity that it is indeed the Stokes matrix in [GGMn21]. In addition we can also extract Stokes constants $\mathcal {S}_{0,j}^{(k)}$ ($j=1,2$, $k=1,2,\ldots$) responsible for Stokes automorphisms into $\Phi ^{(\sigma _0)}(\tau )$ from Borel singularities in the upper half plane, and collect them in the generating series

49 $$\begin{aligned} \textsf{S}^+_{0,j}({\tilde{q}}) = \sum _{k=1}^\infty \mathcal {S}_{0,j}^{(k)}{\tilde{q}}^k,\quad j=1,2. \end{aligned}$$

We find

50 $$\begin{aligned} \textsf{S}_{0,1}^+({\tilde{q}}) = \textsf{S}_{0,2}^+({\tilde{q}})&= -G_0^{(2)}({\tilde{q}}) - G_1^{(2)}({\tilde{q}}) +\left( G_0^{(0)}({\tilde{q}})+G_1^{(0)}({\tilde{q}})\right) G_0^{(2)}({\tilde{q}}) /G_0^{(0)}({\tilde{q}})\nonumber \\&=-{\tilde{q}}-2{\tilde{q}}^2-3{\tilde{q}}^3-7{\tilde{q}}^4-14{\tilde{q}}^5-34{\tilde{q}}^6+\ldots . \end{aligned}$$

Similarly, we find that the global Stokes matrix from cone *III* anti-clockwise to cone *IV* is

51 $$\begin{aligned} \mathfrak {S}_{III\rightarrow IV}({\tilde{q}}) = \begin{pmatrix} 1& 0& 0\\ 0& -1& 1\\ 0& 1& 0 \end{pmatrix}\cdot {\textbf{J}}_{-1}({\tilde{q}}^{-1})^{-1}\cdot \begin{pmatrix} 1& 0& 0\\ 0& -1& 0\\ 0& 0& 1 \end{pmatrix} \cdot {\textbf{J}}_{-1}({\tilde{q}}^{-1})\cdot \begin{pmatrix} 1& 0& 0\\ 0& 1& 0\\ 0& 1& 1 \end{pmatrix},\quad |{\tilde{q}}|>1.\nonumber \\ \end{aligned}$$

It also has the form as (48), and the $2\times 2$ submatrix of $\mathfrak {S}_{III\rightarrow IV}({\tilde{q}})$ in the bottom right is the Stokes matrix given in [GGMn21]. We also extract Stokes constants $\mathcal {S}_{0,j}^{(k)}$ ($j=1,2$, $k=-1,-2,\ldots$) responsible for Stokes automorphisms into $\Phi ^{(\sigma _0)}(\tau )$ from Borel singularities in the lower half plane, and collect them in the generating series

52 $$\begin{aligned} \textsf{S}^-_{0,j}({\tilde{q}}) = \sum _{k=-1}^{-\infty } \mathcal {S}_{0,j}^{(k)}{\tilde{q}}^k,\quad j=1,2. \end{aligned}$$

We find

53 $$\begin{aligned} \textsf{S}_{0,2}^-({\tilde{q}}) = -\textsf{S}_{0,1}^-({\tilde{q}}) = \textsf{S}_{0,1}^+({\tilde{q}}^{-1}). \end{aligned}$$

We can also use (46) to compute the global Stokes matrix $\mathfrak {S}_{IV\rightarrow I}({\tilde{q}})$ and we find

54 $$\begin{aligned} \mathfrak {S}_{IV\rightarrow I} = \begin{pmatrix} 1& 0& 1\\ 0& 1& 3\\ 0& 0& 1 \end{pmatrix}. \end{aligned}$$

Note that this can be identified as $\mathfrak {S}_{0}$ associated to the ray $\rho _0$ and it can be factorised as

55 $$\begin{aligned} \mathfrak {S}_{0} = \mathfrak {S}_{\iota _{0,2}}\mathfrak {S}_{\iota _{1,2}},\quad \mathfrak {S}_{\iota _{0,2}} = \begin{pmatrix} 1& 0& 1\\ 0& 1& 0\\ 0& 0& 1 \end{pmatrix},\quad \mathfrak {S}_{\iota _{1,2}} = \begin{pmatrix} 1& 0& 0\\ 0& 1& 3\\ 0& 0& 1 \end{pmatrix}. \end{aligned}$$

Since the local Stokes matrices $\mathfrak {S}_{\iota _{0,2}}$ and $\mathfrak {S}_{\iota _{1,2}}$ commute, the locality condition is satisfied. We read off the Stoke discontinuity formulas

56 $$\begin{aligned} \begin{aligned}&\text {disc}_0 \Phi ^{(0)}(\tau ) = \tau ^{-3/2}s(\Phi ^{(s_2)})(\tau ),\\&\text {disc}_0 \Phi ^{(1)}(\tau ) = 3s(\Phi ^{(s_2)})(\tau ), \end{aligned} \end{aligned}$$

with

57 $$\begin{aligned} \text {disc}_\theta \Phi (\tau ) = s_{\theta _+}(\Phi )(\tau ) - s_{\theta _-}(\Phi )(\tau ), \end{aligned}$$

and the second identity has already appeared in [GH18, GGMn21].

Finally, in order to compute the global Stokes matrix $\mathfrak {S}_{II\rightarrow III}({\tilde{q}})$, we need to take into account that the odd powers of $\tau ^{1/2}$ on both sides of (39) give rise to additional $-1$ factors when one crosses the branch cut at the negative real axis, and (46) should be modified by

58 $$\begin{aligned} \mathfrak {S}_{II\rightarrow III}({\tilde{q}}) = \textrm{diag}(1,-1,-1) M_{III}({\tilde{q}})^{-1}\cdot M_{II}({\tilde{q}}), \end{aligned}$$

and we find

59 $$\begin{aligned} \mathfrak {S}_{II\rightarrow III} = \begin{pmatrix} 1& 1& 0\\ 0& 1& 0\\ 0& -3& 1 \end{pmatrix}. \end{aligned}$$

Similarly this can be identified as $\mathfrak {S}_{\pi }$ associated to the ray $\rho _{\pi }$ and it can be factorised as

60 $$\begin{aligned} \mathfrak {S}_{\pi } = \mathfrak {S}_{\iota _{0,1}}\mathfrak {S}_{\iota _{2,1}},\quad \mathfrak {S}_{\iota _{0,1}} = \begin{pmatrix} 1& 1& 0\\ 0& 1& 0\\ 0& 0& 1 \end{pmatrix},\quad \mathfrak {S}_{\iota _{2,1}} = \begin{pmatrix} 1& 0& 0\\ 0& 1& 0\\ 0& -3& 1 \end{pmatrix}. \end{aligned}$$

Note that the local Stokes matrices $\mathfrak {S}_{\iota _{0,1}}$ and $\mathfrak {S}_{\iota _{2,1}}$ also commute. We read off the Stokes discontinuity formulas

61 $$\begin{aligned} \begin{aligned} \text {disc}_\pi \Phi ^{(0)}(\tau )&= \tau ^{-3/2}s(\Phi ^{(s_1)})(\tau ),\\ \text {disc}_\pi \Phi ^{(2)}(\tau )&= -3s(\Phi ^{(s_1)})(\tau ), \end{aligned} \end{aligned}$$

where the second identity has already appeared in [GGMn21].

### The Andersen–Kashaev state-integral

In this section we briefly recall the properties of the state-integral of Andersen–Kashaev for the $\textbf{4}_1$ knot [AK14, Sect. 11.4], defined by

62 $$\begin{aligned} Z_{\textbf{4}_1}(\tau ) = \int _{\mathbb {R}+{\textrm{i}}0} \Phi _{\textsf{b}}(v)^2 \, {\textrm{e}}^{-\pi {\textrm{i}}v^2} {\textrm{d}}v, \qquad (\tau =\sqrt{{\textsf{b}}}) . \end{aligned}$$

Here, ${\Phi }_{{\textsf{b}}}(z)$ is Faddeev’s quantum dilogarithm [Fad95], in the conventions of e.g. [AK14, Appendix A]. With this choice of contour, the integrand is exponentially decaying at $\pm \infty$ hence the integral is absolutely convergent. State-integrals have several key features:

- They are analytic functions in $\mathbb {C}'$.
- Their restriction to $\mathbb {C}\setminus \mathbb {R}$ factorises bilinearly as finite sum of a product of a *q*-series and a ${{\tilde{q}}}$-series, where $q=\textbf{e}(\tau )$ and ${{\tilde{q}}}=\textbf{e}(-1/\tau )$; see [BDP14, Pas12, GK17].
- Their evaluation at positive rational numbers also factorises bilinearly as a finite sum of a product of a periodic function of $\tau$ and a periodic function of $-1/\tau$; see [GK15].
- State-integrals are equal to linear combinations of the median Borel summation of asymptotic series.
- State-integrals come with a descendant version which satisfies a linear *q*-difference equation.

Let us explain these properties for the state-integral (62). The integrand is a quasi-periodic meromorphic function with explicit poles and residues. Moving the contour of integration above, summing up the residue contributions, and using the fact that there are no contributions from infinity, one finds that [GK17, Cor.1.7]

63 $$\begin{aligned} Z(\tau ) = -{\textrm{i}}\left( \frac{q}{{\tilde{q}}}\right) ^{\frac{1}{24}} \left( \tau ^{1/2}G^{(1)}(q)G^{(0)}({\tilde{q}}) - \tau ^{-1/2} G^{(0)}(q)G^{(1)}({\tilde{q}})\right) , \qquad (\tau \in \mathbb {C}\setminus \mathbb {R}).\nonumber \\ \end{aligned}$$

When $\tau$ is a positive rational number, the quasi-periodicity of the integrand, together with a residue calculation leads to a formula for $Z(\tau )$ given in [GK15]. More generally, in [GGMn21] we considered the descendant integral

64 $$\begin{aligned} Z_{\lambda ,\mu }(\tau ) = \int _{{\mathcal {D}}} \Phi _{{\textsf{b}}}(v)^2 {\textrm{e}}^{-\pi {\textrm{i}}v^2 + 2\pi (\lambda {\textsf{b}}- \mu {\textsf{b}}^{-1})v}{\textrm{d}}v, \end{aligned}$$

where $\lambda , \mu \in \mathbb {Z}$ and the contour ${\mathcal {D}}$ is asymptotic at infinity to the horizontal line $\textrm{Im}(v)=v_0$ where $v_0 > |{{\,\textrm{Re}\,}}(\lambda {\textsf{b}}-\mu {\textsf{b}}^{-1})|$ but is deformed near the origin so that all the poles of the quantum dilogarithm located at

65 $$\begin{aligned} c_{\textsf{b}}+ {\textrm{i}}{\textsf{b}}m +{\textrm{i}}{\textsf{b}}^{-1}n,\quad m,n\in {\mathbb {Z}}_{\ge 0}, \end{aligned}$$

are above the contour. These integrals factorise as follows:

66 $$\begin{aligned} Z_{\lambda ,\mu }(\tau ) = (-1)^{\lambda -\mu +1}{\textrm{i}}q^{\frac{\lambda }{2}}{\tilde{q}}^{\frac{\mu }{2}} \left( \frac{q}{{\tilde{q}}}\right) ^{\frac{1}{24}} \left( \tau ^{1/2}G^{(1)}_\lambda (q)G^{(0)}_\mu ({\tilde{q}}) - \tau ^{-1/2} G^{(0)}_\lambda (q)G^{(1)}_\mu ({\tilde{q}})\right) .\nonumber \\ \end{aligned}$$

The above factorisation can be expressed neatly in matrix form. Indeed, let us define

67 $$\begin{aligned} W^\text {red}_{S,\lambda ,\mu }(\tau ) = {\textbf{J}}^\text {red}_\lambda ({\tilde{q}})^{-1} \textrm{diag}(\tau ^{3/2}, \tau ^{1/2}, \tau ^{-1/2}) \, {\textbf{J}}^\text {red}_\mu (q) . \end{aligned}$$

Using the *q*-difference equation (13), it is easy to see that $W^\text {red}_{S,\lambda +1,\mu }(\tau )=A^{-1}(-1/\tau ) W^\text {red}_{S,\lambda ,\mu }(\tau )$ and $W^\text {red}_{S,\lambda ,\mu +1}(\tau )=W^\text {red}_{S,\lambda ,\mu }(\tau ) A(\tau )$ hence the domain of $W^\text {red}_{S,\lambda ,\mu }$ is independent of the integers $\lambda$ and $\mu$. Equation (66) implies that $W^\text {red}_{S,\lambda ,\mu }(\tau )$ are given by the matrix $(Z_{\lambda +i,\mu +j}(\tau ))$ (for $i,j =0,1$), up to left-multiplication by a matrix of automorphy factors.

Finally we discuss the relation between the Borel summation of the two asymptotic series $\Phi ^{(\sigma _j)}(h)$ for $j=1,2$ and the descendant state-integrals. Since the Borel transform of those series may have singularities at the positive real axis, we denote by $s_{{\textrm{med}}}$ their *median resummation* given by the average of the two Laplace transforms to the left and to the right of the positive real axis. Then, we have

68 $$\begin{aligned} \begin{aligned} s_{{\textrm{med}}} ( \Phi ^{(\sigma _1)} ) (\tau )&= {\textrm{i}}({\tilde{q}}/q)^{1/24} \left( -\frac{1}{2}Z_{0,0}(\tau )-{\tilde{q}}^{1/2} Z_{0,-1}(\tau )\right) , \\ s_{{\textrm{med}}} ( \Phi ^{(\sigma _2)} ) (\tau )&= {\textrm{i}}({\tilde{q}}/q)^{1/24} Z_{0,0}(\tau ) . \end{aligned} \end{aligned}$$

### A new state-integral

In the previous section, we saw how the matrix $W^\text {red}(\tau )$ of products of *q*-series and ${\tilde{q}}$-series (6) coincides with a matrix of state-integrals. Having found the *q*-series (9) which complement the series (6), it is natural to search for a new state-integral which factorises in terms of all three *q*-series $G_{m}^{(j)}(q)$ for $j=0,1,2$ and their ${\tilde{q}}$-versions. Upon looking carefully, the series $G_{m}^{(j)}(q)$ for $j=0,1$ were produced from the Andersen–Kashaev state-integral because its integrand had a double pole, hence the contributions came from expanding (10) up to $O(\varepsilon ^2)$. If we expanded up to $O(\varepsilon ^3)$, we would capture the new series $G^{(2)}(q)$. Hence the problem is to find a state-integral of the $\textbf{4}_1$ whose integrand has poles of order 3. After doing so, one needs to understand how this story, which seems a bit ad hoc and coincidental to the $\textbf{4}_1$ knot, can generalise to all knots. It turns out that such a state-integral existed in the literature for many years, and in fact was devised by Kashaev [Kas97] as a method to convert the state-sums of the Kashaev invariants into state-integrals, using as a building block the Faddeev quantum dilogarithm function at rational numbers, multiplied by $1/\sinh x$. Incidentally, similar integrals have appeared in [KMn16] and more recently in the work of two of the authors on the topological string on local $\mathbb {P}^2$; see [GMn, Eq. 3.141]. The integrand of such state-integrals are meromorphic functions with the usual pole structure coming from the Faddeev quantum dilogarithm function, together with the extra poles coming from $1/\sinh x$. The residues of the former give rise to products of *q*-series times ${\tilde{q}}$-series, but the presence of of $1/\sinh x$ has two effects. On the one hand, it produces, in an asymmetric fashion, poles of the integrand of one order higher, contributing to sums of *q*-series or ${\tilde{q}}$-series. On the other hand, the produced *q* and ${\tilde{q}}$-series look like multidimensional Appell-Lerch sums. An original motivation for converting state-sum formulas for the Kashaev invariants into state-integral formulas was to use such an integral expression for a proof of the Volume Conjecture.

**Fig. 3:**
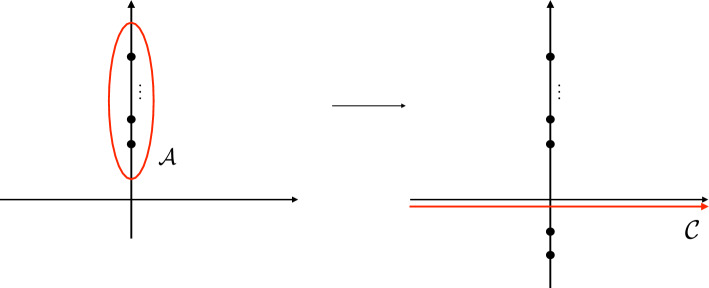
The contour ${{\mathcal {A}}}_N$ appears in the integral formula (69) for the Kashaev invariant of the $\textbf{4}_1$ knot, and it encircles the *N* poles (71). By doing the integral along the contour ${\mathcal {C}}$ and picking the poles in the lower half plane, one obtains a new state-integral with information about the trivial connection

There are two examples that convert state-sums into state-integrals, one given by Kashaev in [Kas97] for the $\textbf{4}_1$ knot and further studied by Andersen–Hansen [AH06], and one in Kashaev–Yokota [KY] for the $\textbf{5}_2$ knot. In the case of the $\textbf{4}_1$ knot, the integral considered in [Kas97, AH06] is

69 $$\begin{aligned} \langle \textbf{4}_1 \rangle _N =-{{\textrm{i}}\over 2 {{\textsf{b}}}^3} \int _{{{\mathcal {A}}}_N} \tanh \left( {\pi y \over {{\textsf{b}}}} \right) {{\Phi }_{{\textsf{b}}}\left( -y +{{\textrm{i}}\over 2 {{\textsf{b}}}} \right) \over {\Phi }_{{\textsf{b}}}\left( y -{{\textrm{i}}\over 2 {{\textsf{b}}}} \right) } {\textrm{d}}y. \end{aligned}$$

For generic ${\textsf{b}}^2\in {\mathbb {C}}'$ so that ${{\,\textrm{Re}\,}}{\textsf{b}}>0$, the integrand has the following poles and zeros, all in the imaginary axis:

70 $$\begin{aligned} \begin{aligned}&\text {simple poles}: \left\{ {\textrm{i}}{\textsf{b}}\left( \frac{1}{2}+m\right) \,\big |\, m=0,1,2,\ldots \right\} ,\\&\text {double poles}: \left\{ -{\textrm{i}}{\textsf{b}}\left( \frac{1}{2}+m\right) -{\textrm{i}}{\textsf{b}}^{-1}(1+n) \,\big |\, m,n=0,1,2,\ldots \right\} , \\&\text {triple poles}: \left\{ -{\textrm{i}}{\textsf{b}}\left( \frac{1}{2}+m\right) \,\big |\, m=0,1,2,\ldots \right\} ,\\&\text {double zeros}: \left\{ {\textrm{i}}{\textsf{b}}\left( \frac{1}{2}+m\right) +{\textrm{i}}{\textsf{b}}^{-1}(1+n)\,\big |\,m,n=0,1,2,\ldots \right\} . \end{aligned} \end{aligned}$$

In the special case where ${{\textsf{b}}}^2=N^{-1}$ where $N \in \mathbb {Z}_{>0}$, which is the case where (69) is well-defined, the poles and zeros in the upper half plane conspire so that there are only finite many simple poles located at

71 $$\begin{aligned} y_m={\textrm{i}}{{\textsf{b}}}\left( m+{1\over 2} \right) , \qquad m=0, \cdots , N-1, \end{aligned}$$

and we can define the contour ${{\mathcal {A}}}_N$ encircling these points as in Fig. 3(left). An application of the residue theorem gives that this integral calculates the Kashaev invariant of the $\textbf{4}_1$ knot,

72 $$\begin{aligned} \langle \textbf{4}_1 \rangle _N=\sum _{m=0}^{N-1} (-1)^m \xi ^{-m(m+1)/2} \prod _{\ell =1}^m (1- \xi ^\ell )^2, \qquad \xi = {\textrm{e}}^{2\pi {\textrm{i}}\over N}. \end{aligned}$$

Now, we can define a new analytic function by changing the contour of integration from ${\mathcal {A}}_N$ to the horizontal contour ${\mathcal {C}}$ slightly below the horizontal line ${{\,\textrm{Im}\,}}(y) = {{\,\textrm{Re}\,}}({\textsf{b}}^{-1})/2$,

73 $$\begin{aligned} { \mathcal {Z}}(\tau )= -\frac{{\textrm{i}}}{2{\textsf{b}}^3}\int _{{\mathcal {C}}} \tanh \left( {\pi y \over {{\textsf{b}}}} \right) {{\Phi }_{{\textsf{b}}}\left( -y +{{\textrm{i}}\over 2 {{\textsf{b}}}} \right) \over {\Phi }_{{\textsf{b}}}\left( y -{{\textrm{i}}\over 2 {{\textsf{b}}}} \right) } {\textrm{d}}y. \end{aligned}$$

This is now defined for $\tau = {{\textsf{b}}}^2 \in \mathbb {C}'$. Although both (69) and (73) share the same integrand, it has significant contributions from infinity in the upper half plane, so that we cannot deform the contour $\mathcal {A}_N$ smoothly to the contour $\mathcal {C}$, and (69) and (73) are really different. On the other hand, the integrand does have vanishing contributions from infinity in the lower half plane. Consequently we can smoothly deform the new controur $\mathcal {C}$ downwards, and collect the residues of the integrand on the lower half-plane, as shown in Fig. 3(right). This integral, in contrast to the Andersen–Kashaev state-integral, contains information about the trivial connection. In particular, we conjecture that, in the region of the complex $\tau$-plane slightly above the positive real axis, the all-orders asymptotic of $\mathcal {Z}(\tau )$ at $\tau =0$ is given by

74 $$\begin{aligned} { \mathcal {Z}}(\tau )\sim \Phi ^{(\sigma _0)}(\tau ). \end{aligned}$$

Moreover, this can be upgraded to an exact asymptotic formula by using Borel resummation in the same region, and one has

75 $$\begin{aligned} { \mathcal {Z}}(\tau )= s( \Phi ^{(\sigma _0)} )(\tau ) -{{\textrm{i}}\over 2}\tau ^{-3/2} s(\Phi ^{(\sigma _2)} )(\tau ). \end{aligned}$$

It turns out that the change of contour in Fig. 3 implements the inversion of the Habiro series recently studied in [Par]: the integral over the contour ${{\mathcal {A}}}_N$ leads to the Habiro series, while the integral over ${\mathcal {C}}$ gives the “inverted” Habiro series, see also Sect. 3.4. This inversion between *q*-series and elements of the Habiro ring was observed 10 years ago by the first author in his joint work with Zagier [GZ23], under the informal name “upside-down cake”.

### A $3 \times 3$ matrix of state-integrals

Having found a new state-integral whose asymptotics sees the asymptotic series $\Phi ^{(\sigma _0)}(\tau )$, we now consider its descendants, and their factorisations to complete the story. The new state-integrals $\mathcal {Z}_{\lambda ,\mu }(\tau )$ are defined as follows:

76 $$\begin{aligned} {\mathcal {Z}}_{\lambda , \mu }(\tau ) = -\frac{{\textrm{i}}}{2{\textsf{b}}^3}\int _{{\mathcal {C}}} \tanh \left( {\pi y \over {{\textsf{b}}}} \right) {{\Phi }_{{\textsf{b}}}\left( -y +{{\textrm{i}}\over 2 {{\textsf{b}}}} \right) \over {\Phi }_{{\textsf{b}}}\left( y -{{\textrm{i}}\over 2 {{\textsf{b}}}} \right) } {\textrm{e}}^{-2 \pi (\lambda {{\textsf{b}}}- \mu {{\textsf{b}}}^{-1})y} {\textrm{d}}y, \end{aligned}$$

where ${{\textsf{b}}}$ is related to $\tau$ by $\tau = {{\textsf{b}}}^2$ and $\lambda ,\mu \in \mathbb {Z}$. The integration contour ${{\mathcal {C}}}$ is chosen so that, at infinity, it is asymptotic to the line ${\textrm{Im}}(y)=y_2$, where $y_2$ satisfies

77 $$\begin{aligned} y_2 < \tfrac{1}{2}{{\,\textrm{Re}\,}}{\textsf{b}}^{-1} - |{{\,\textrm{Re}\,}}(\lambda {\textsf{b}}-\mu {\textsf{b}}^{-1})|. \end{aligned}$$

This guarantees convergence of the integral. We choose ${\mathcal {C}}$ so that all poles of the integrand in the lower half plane are below ${\mathcal {C}}$. Note that $\mathcal {Z}_{0,0}(\tau )=\mathcal {Z}(\tau )$ is the integral introduced in (73), so that the state-integrals with general $\lambda ,\mu$ are descendants of $\mathcal {Z}(\tau )$.

#### Theorem 7

The descendant state-integral (76) can be expressed in terms of the series (6), (11) as follows:

78 $$\begin{aligned} \begin{aligned} {\mathcal {Z}}_{\lambda , \mu }(\tau )&= q^{\lambda /2} (-1)^{\mu } \left( G_\lambda ^{(2)}(q)+\tau ^{-1} G_\lambda ^{(1)}(q) L_\mu ^{(0)}({\tilde{q}}) -\tau ^{-2} G_\lambda ^{(0)}(q) L_\mu ^{(1)}({\tilde{q}}) \right) \\&\quad +\frac{1}{2} q^{\lambda /2} (-1)^{\mu } \left( \tau ^{-1} G_\lambda ^{(1)}(q) G_\mu ^{(0)}({\tilde{q}}) - \tau ^{-2} G_\lambda ^{(0)}(q) G_\mu ^{(1)}({\tilde{q}}) \right) \end{aligned} \end{aligned}$$

#### Proof

This follows by applying the residue theorem to the state-integral (76), along the lines of the proof of Theorem 1.1 in [GK17]. One closes the contour to encircle the poles in the lower half-plane, located at

79 $$\begin{aligned} y_{m,n}= -{{\textrm{i}}{{\textsf{b}}}\over 2} -{\textrm{i}}m {{\textsf{b}}}-{\textrm{i}}n {{\textsf{b}}}^{-1}, \qquad m,n\ge 0. \end{aligned}$$

The poles of the integrand come the poles and the zeros of the quantum dilogarithm as well as from the $\tanh$ function. When $n=0$ they are triple (a double pole comes from the quantum dilogarithm and a simple pole from $\tanh$), while those with $n>0$ are double, coming only from the quantum dilogarithm. The triple poles lead to the series $G_\lambda ^{(2)}(q)$. In order to obtain the final result, one also has to use the properties of $E_2(q)$ under modular transformations, i.e.

80 $$\begin{aligned} E_2({\tilde{q}})= \tau ^2 \left( E_2 (q) + {12 \over 2 \pi {\textrm{i}}\tau } \right) . \end{aligned}$$

$$\square $$

#### Remark 8

The state-integral (76) can be evaluated for arbitrary rational values of $\tau$ by using the techniques of [GK15]. One finds for example, for ${{\textsf{b}}}^2=1$,

81 $$\begin{aligned} \mathcal {Z} (1)= -2 \sinh ^2 \left( {V \over 4 \pi } \right) , \end{aligned}$$

where *V* is the hyperbolic volume of $\textbf{4}_1$.

#### Remark 9

Equation (75) can be written as

82 $$\begin{aligned} \mathcal {Z}(\tau )= \, s_{{\textrm{med}}} ( \Phi ^{(\sigma _0)} ) (\tau ), \qquad \tau >0. \end{aligned}$$

We now discuss an important analytic extension of the matrix ${\textbf{J}}_\mu (q)$ defined for $|q| \ne 1$. We define

83 $$\begin{aligned} W_{S,\lambda ,\mu }(\tau ) = {\textbf{J}}_\lambda ({\tilde{q}})^{-1} \textrm{diag}(\tau ^{3/2}, \tau ^{1/2}, \tau ^{-1/2}) \, {\textbf{J}}_\mu (q) \qquad (\tau \in \mathbb {C}\setminus \mathbb {R}). \end{aligned}$$

As in Sect. 2.5, we find that the domain of $W_{S,\lambda ,\mu }$ is independent of the integers $\lambda$ and $\mu$.

#### Theorem 10

$W_{S,\lambda ,\mu }(\tau )$ extends to a holomorphic function on $\mathbb {C}'$ and equals to the matrix $(Z_{\lambda +i,\mu +j}(\tau ))$ (for $i,j =0,1,2$), up to left-multiplication by a matrix of automorphy factors.

#### Proof

For the bottom block of four entries, this result is already known from [GGMn21, GGMn23], and it follows from (66) as was discussed in Sect. 2.5. The top two non-trivial entries $(\sigma _0,\sigma _j)$ of $W_{S,\lambda ,\mu }(\tau )$ for $j=1,2$ are given by

84 $$\begin{aligned} \tau ^{3/2}\left( G_{\mu -1+j}^{(2)}(q) +\tau ^{-1} G_{\mu -1+j}^{(1)}(q)L_{\lambda }^{(0)}({\tilde{q}}) - \tau ^{-2} G_{\mu -1+j}^{(0)}(q)L_{\lambda }^{(1)}({\tilde{q}}) \right) . \end{aligned}$$

In view of Theorem 7 and (66) they can be written as a sum of state-integrals ${\mathcal {Z}}_{\lambda , \mu }(\tau )$ and ${\mathcal {Z}}_{\lambda , \mu +1}(\tau )$, multiplied by holomorphic factors. This proves the theorem. $\square$

## The *x*-Variable

In this section we discuss an extension of the results of Sect. 2 adding an *x*-variable. In the context of the *n*th colored Jones polynomial, $x=q^n$ corresponds to an eigenvalue of the meridian in the asymptotic expansion of the Chern–Simons path integral around an abelian representation of a knot complement. In the context of the state-integral of Andersen-Kashaev [AK14], the *x*-variable is the monodromy of a peripheral curve. The corresponding state-integral factorises bilinearly into holomorphic blocks, which are functions of (*x*, *q*) and $({\tilde{x}},{\tilde{q}})$ [BDP14]. In the context of quantum modular forms, *x* plays the role of a Jacobi variable.

The corresponding perturbative series are now *x*-deformed (see [GGMn23, Sect. 5.1]), but there are some tricky aspects of this deformation that we now discuss. The critical points of the action, after exponentiation, lie in a plane curve *S* in $(\mathbb {C}^*)^2$ (the so-called spectral curve) defined over the rational numbers, where $(\mathbb {C}^*)^2$ is equipped with coordinate functions *x* and *y*. The field $\mathbb {Q}(S)$ of rational functions of *S* (assuming *S* is irreducible, or working with one component of *S* at a time) can be identified with $\mathbb {Q}(x)[y]/(p(x,y))$ where $p(x,y)=0$ is the defining polynomial of *S*. The coefficients of the perturbative series are elements of $(\mathbb {Q}(S)^*)^{-1/2} \mathbb {Q}(S)$ and the perturbative series are labeled by the branches of the projection $S \rightarrow \mathbb {C}^*$ corresponding to $(x,y) \mapsto y$ (with discriminant $\delta$, a rational function on *S*). Each such branch $\sigma$ defines locally an algebraic function $y=y^\sigma =y^\sigma (x) \in {\overline{\mathbb {Q}}}(x)$ satisfying the equation $p(x,y^\sigma (x))=0$, which gives rise to an embedding of the field of $\mathbb {Q}(S)$ to the field ${\overline{\mathbb {Q}}}(x)$ of algebraic functions obtained by replacing *y* by $y^\sigma (x)$. For each such branch $\sigma$, the perturbative series has the form

85 $$\begin{aligned} \Phi ^{(\sigma )}(x,\tau ) = e^{\tfrac{V^{\sigma }(x)}{2\pi {\textrm{i}}\tau }} \varphi ^{(\sigma )}(x,\tau ) \end{aligned}$$

where $\varphi ^{(\sigma )}(x,\tau ) \in \frac{1}{\sqrt{i\delta _\sigma (x)}} {\overline{\mathbb {Q}}}(x)[[2\pi {\textrm{i}}\tau ]]$. The volume $V^{\sigma }(x)$ is also a function of *x* given explicitly as a sum of dilogarithms and products of logarithms.

In the above discussion it is important to keep in mind that the asymptotic series (85) are labeled by branches of the finite ramified covering $S \rightarrow \mathbb {C}^*$. Going around a loop in *x*-space that avoids the finitely many ramified points *will* change the labeling of the $y=y(x)$ branches, and correspondingly of the asymptotic series. In the present paper (as well as in [GGMn23]), we define the asymptotic series in a neighborhood of $x \sim 1$ of the geometric representation, and we do not discuss the *x*-monodromy question.

In the case of the $\textbf{4}_1$ knot, the asymptotic series associated to the geometric, and the conjugate flat connections are given by

86 $$\begin{aligned} \begin{aligned} \varphi ^{(\sigma _1)}(x;\tfrac{h}{2\pi {\textrm{i}}})&= \frac{1}{\sqrt{\delta (x)}} \left( 1-\frac{{\textrm{i}}(x^{-3}-x^{-2}-2x^{-1}+15-2x-x^2+x^3)}{24\delta (x)^3} h+\ldots \right) \\ \varphi ^{(\sigma _2)}(x;\tfrac{h}{2\pi {\textrm{i}}})&= \frac{{\textrm{i}}}{\sqrt{\delta (x)}} \left( 1+\frac{{\textrm{i}}(x^{-3}-x^{-2}-2x^{-1}+15-2x-x^2+x^3)}{24\delta (x)^3} h +\ldots \right) \end{aligned}\nonumber \\ \end{aligned}$$

with $$h=2\pi {\textrm{i}}\tau $$ and

87 $$\begin{aligned} \delta (x) = \sqrt{-x^{-2}+2x^{-1}+1+2x-x^2}. \end{aligned}$$

The corresponding perturbative series are defined by

88 $$\begin{aligned} \begin{aligned} \Phi ^{(\sigma _1)}(x;\tau )&= {\textrm{e}}^{\tfrac{A(x)}{2\pi {\textrm{i}}\tau }} \varphi ^{(\sigma _1)}(x;\tau ),\\ \Phi ^{(\sigma _2)}(x;\tau )&= {\textrm{e}}^{-\tfrac{A(x)}{2\pi {\textrm{i}}\tau }} \varphi ^{(\sigma _2)}(x;\tau ), \end{aligned} \end{aligned}$$

where

89 $$\begin{aligned} A(x) = \frac{1}{2} \log (t)^2 + 2 \log (t) \log (x) + \log (x)^2 + \textrm{Li}_2(-t x) + \textrm{Li}_2(-t)+ \frac{\pi ^2}{6} + \pi {\textrm{i}}\log (x),\nonumber \\ \end{aligned}$$

with $t(x) = \frac{-1-x^{-1}+x -{\textrm{i}}\delta (x)}{2}$ being a solution to the equation $(t+x^{-1})+(t+x^{-1})^{-1}=x+x^{-1}-1$. Note that when $x=1$, $\delta (1)=\sqrt{3}$, $t(1)=-\frac{1+{\textrm{i}}\sqrt{3}}{2}$ and $\Phi ^{(\sigma _j)}(1;\tau )=\Phi ^{(\sigma _j)}(\tau )$, the latter defined in Sect. 2.3.

### The $\Phi ^{(\sigma _0)}(x,\tau )$ series

We begin by discussing the perturbative series $\varphi ^{(\sigma _0)}(x,\tau )$ which is a formal power series in $2\pi {\textrm{i}}\tau$ whose coefficients are rational functions of *x* with rational coefficients. The series is defined by the right hand side of Equation (25) after setting $h=2\pi {\textrm{i}}\tau$. One way to compute the $\ell$-th coefficient of that series is by computing the colored Jones polynomial, expanding in *n* and *h* as in (23) and then resumming as in (26), taking into account the fact that the latter sum is a rational function. An alternative way is by using Habiro’s expansion of the colored Jones polynomials [Hab02b] (see also [Hab08])

90 $$\begin{aligned} J^K(x,q) = \sum _{k=0}^\infty c_{k}(x,q) H^K_k(q), \qquad c_{k}(x,q) = x^{-k}(q x;q)_k (q^{-1}x;q^{-1})_k \end{aligned}$$

where $H^K_k(q) \in \mathbb {Z}[q^\pm ]$ are the Habiro polynomials of the knot *K* and $J^K(q^n,q)$ is the *n*th colored Jones polynomial. The latter can be efficiently computed using a recursion (which always exists [GL05]) together with initial conditions. This is analogous to applying the WKB method to a corresponding linear *q*-difference equation [DGLZ09, Gar08b]. We comment that the colored Jones polynomials of a knot *K* have a descendant version defined by [GK23]

91 $$\begin{aligned} DJ^{K,(m)}(x,q) = \sum _{k=0}^\infty c_{k}(x,q) H^K_k(q) \, q^{km}, \qquad (m \in \mathbb {Z}). \end{aligned}$$

Correspondingly, the Kashaev invariant has a descendant version $DJ^{K,(m)}(1,q)$ (an element of the Habiro ring) and the asymptotic series $\Phi ^{(\sigma _0)}(x,\tau )$ have a descendant version $\Phi ^{(\sigma _0)}_m(x,\tau )$ defined for all integers *m* in [GK23], which we will not use in the present paper.

Going back to the case of the $\textbf{4}_1$ knot, we have

92 $$\begin{aligned} \varphi ^{(\sigma _0)}(x;\tfrac{h}{2\pi {\textrm{i}}})&= -\frac{1}{x^{-1}-3+x} -\frac{x^{-1}-1+x}{(x^{-1}-3+x)^4}h^2 \nonumber \\&\quad -\frac{x^{-4}+14x^{-3}+64x^{-2}-156x^{-1} +201-156x+64x^2+14x^3+x^4}{12(x^{-1}-3+x)^7}h^4 +\ldots \end{aligned}$$

and the corresponding perturbative series is given by $\Phi ^{(\sigma _0)}(x;\tau ) = \varphi ^{(\sigma _0)}(x;\tau )$.

### A $3\times 3$ matrix of (*x*, *q*)-series

We now extend the results of Sect. 2.2 by including the Jacobi variable *x* which, on the representation side, determines the monodromy of the meridian of an $\textrm{SL}_2(\mathbb {C})$ representation $\sigma$.

Our first task is to define the $3 \times 3$ matrix ${\textbf{J}}_{m}(x,q)$. For $|q| \ne 1$, we define

93 $$\begin{aligned} \begin{aligned} C_{m}(x,q)&=\sum _{k=0}^{\infty }(-1)^{k}\frac{q^{k(k+1)/2 +km}}{(x^{-1};q)_{k+1}(x;q)_{k+1}}\\ A_{m}(x,q)&=\sum _{k=0}^{\infty }(-1)^{k}\frac{q^{k(k+1)/2 +km}x^{k+m}}{(q;q)_{k}(x^{2}q;q)_{k}}\\ B_{m}(x,q)&=A_{m}(x^{-1},q). \end{aligned} \end{aligned}$$

Our series $C_m(x,q)$ contain as a special case the series $F_{\textbf{4}_1}(x,q)$ in [GM21, Par20, Par]

94 $$\begin{aligned} F_{\textbf{4}_1}(x,q) = (x^{1/2}-x^{-1/2}) C_0(x,q). \end{aligned}$$

We assemble these (*x*, *q*)-series into a matrix

95 $$\begin{aligned} {\textbf{J}}_{m}(x,q) = \begin{pmatrix} 1 & C_{m}(x,q) & C_{m+1}(x,q)\\ 0 & A_{m}(x,q) & A_{m+1}(x,q)\\ 0 & B_{m}(x,q) & B_{m+1}(x,q)\\ \end{pmatrix} \end{aligned}$$

whose bottom-right $2 \times 2$ matrix is ${\textbf{J}}^\text {red}_{m}(x,q)$. The properties of ${\textbf{J}}_{m}(x,q)$ are summarised in the next theorem.

#### Theorem 11

The matrix ${\textbf{J}}_{m}(x,q)$ is a fundamental solution to the linear *q*-difference equation

96 $$\begin{aligned} {\textbf{J}}_{m+1}(x,q) = {\textbf{J}}_{m}(x,q) A(x,q^m,q), \qquad A(x,q^m,q)= \begin{pmatrix} 1 & 0 & 1\\ 0 & 0 & -1\\ 0 & 1 & x^{-1}+x-q^{m+1}\\ \end{pmatrix}\nonumber \\ \end{aligned}$$

has $\det ({\textbf{J}}_m(x,q))=x^{-1}-x$ and satisfies the analytic extension

97 $$\begin{aligned} {\textbf{J}}_m(x,q^{-1}) = \begin{pmatrix} 1& 0& 0\\ 0& 0& 1\\ 0& 1& 0 \end{pmatrix} {\textbf{J}}_{-m-1}(x,q) \begin{pmatrix} 1& 0& 0\\ 0& 0& 1\\ 0& 1& 0 \end{pmatrix} . \end{aligned}$$

#### Proof

The proof is analogous to the proof of Theorem 3. Equation (96) follows quickly using the *q*-hypergeometric expressions and noting that $C_{m}(x,q)$ has a boundary term so satisfies an inhomogenous version. The block form again reduces the calculation of the determinant of ${\textbf{J}}_{m}(x,q)$ to a calculation of the determinant of ${\textbf{J}}^\text {red}_{m}(x,q)$ given in [GGMn23]. Equation (97) follows from the symmetry of the *q*-hypergeometric functions

98 $$\begin{aligned} \begin{aligned} C_{m}(x,q^{-1})&=C_{-m}(x,q)\\ A_{m}(x,q^{-1})&=B_{-m}(x,q)\\ B_{m}(x,q^{-1})&=A_{-m}(x,q). \end{aligned} \end{aligned}$$

$$\square $$

The Appell-Lerch like sums again appear in the inverse of ${\textbf{J}}_{m}(x,q)$. The proof is again completely analogous to the proof of Theorem 4.

#### Theorem 12

We have

99 $$\begin{aligned} {\textbf{J}}_{m}(x,q)^{-1} = \frac{1}{x^{-1}-x}\begin{pmatrix} x^{-1}-x & -LB_{m}(x,q) & LA_{m}(x,q)\\ 0 & B_{m+1}(x,q) & -A_{m+1}(x,q)\\ 0 & -B_{m}(x,q) & A_{m}(x,q) \end{pmatrix} \end{aligned}$$

for the *q*-series $LA_m(x,q),LB_m(x,q)$ defined by

100 $$\begin{aligned} \begin{aligned} LA_{m}(x,q)&= A_{m+1}(x,q)C_{m}(x,q)-A_{m}(x,q)C_{m+1}(x,q)\\ LB_{m}(x,q)&= B_{m+1}(x,q)C_{m}(x,q)-B_{m}(x,q)C_{m+1}(x,q) \end{aligned} \end{aligned}$$

The *q*-series $LA_m(x,q),LB_m(x,q)$ are expressed in terms of Appell-Lerch type sums:

101 $$\begin{aligned} \begin{aligned} LA_{m}(x,q)&= \sum _{k=0}^{\infty }(-1)^{k} \frac{q^{k(k+1)/2+km+k}x^{k+m+1}}{(q;q)_{k}(x^{2}q;q)_{k}(1-xq^{k})} \\ LB_{m}(x,q)&= LA_{m}(x^{-1},q) . \end{aligned} \end{aligned}$$

#### Proof

Given the block form of ${\textbf{J}}_{m}(x,q)$ and the determinant calculated previously in Theorem 11, Equation (100) follows from taking the matrix inverse. Observe that again $A(x;q^m,q)$ has first column $(1,0,0)^t$ and first row (1, 0, 1). It follows that its inverse matrix has first column $(1,0,0)^t$ and first row (1, 1, 0). This, together with (96), implies that

102 $$\begin{aligned} \begin{aligned} {\textbf{J}}_{m+1}(x,q)^{-1}&= A(x,q^m,q)^{-1} {\textbf{J}}_{m}(x,q)^{-1}\\&= \begin{pmatrix} 1 & 1 & 0\\ 0 & x+x^{-1}-q^{m+1} & 1\\ 0 & -1 & 0\\ \end{pmatrix} {\textbf{J}}_{m}(x,q)^{-1} \end{aligned} \end{aligned}$$

which implies that $LA_m(x,q),LB_{m}(x,q)$ satisfy the first order inhomogeneous linear *q*-difference equation

103 $$\begin{aligned} \begin{aligned} LA_{m-1}(x,q)-LA_{m}(x,q)&= A_{m}(x,q),\\ LB_{m-1}(x,q)-LB_{m}(x,q)&= B_{m}(x,q). \end{aligned} \end{aligned}$$

Let ${\mathcal {L}}A_m(x,q)$ denote the right-hand side of the first Equation (101). Then we have

$$\begin{aligned} {\mathcal {L}}A_{m-1}(x,q)-{\mathcal {L}}A_{m}(x,q) = \sum _{k=0}^{\infty }(-1)^{k} \frac{q^{k(k+1)/2+km}x^{k+m}(1-xq^{k})}{(q;q)_{k}(x^{2}q;q)_{k}(1-xq^{k})} = A_{m}(x,q). \end{aligned}$$

Therefore ${\mathcal {L}}A_{m}(x,q)-LA_{m}(x,q)$ is independent of *m*. Moreover, $\lim _{m \rightarrow \infty } {\mathcal {L}}A_{m}(x,q)-LA_{m}^{(0)}(x,q)=0$ for $|q|,|x|<1$ or $\lim _{m \rightarrow -\infty } {\mathcal {L}}A_{m}(x,q)-LA_{m}^{(0)}(x,q)=0$ for $|q|,|x|>1$. Equations (101) follows from analytic continuation. $\square$

Now if we take the inverse of ${\textbf{J}}_{m}(x,q)^{-1}$ we can get similar identities for $C_{m}(x,q)$.

#### Corollary 13

104 $$\begin{aligned} C_{m}(x,q)&=\frac{1}{x^{-1}-x}\left( A_{m}(x,q) LB_{m}(x,q)-B_{m}(x,q)LA_{m}(x,q)\right) \end{aligned}$$

105 $$\begin{aligned} C_{m+1}(x,q)&=\frac{1}{x^{-1}-x}\left( A_{m+1}(x,q)LB_{m}(x,q) -B_{m+1}(x,q)LA_{m}(x,q)\right) \,. \end{aligned}$$

### Borel resummation and Stokes constants

In this section we extend the discussion in Sect. 2.4 to include *x*-deformation. We analyse the asymptotic expansion as $q = {\textrm{e}}^{2\pi {\textrm{i}}\tau }$ and $\tau \rightarrow 0$ of the (*x*, *q*)-series presented in Sect. 3.2 and relate them to the $(x,\tau )$-asymptotic series given in Sect. 3.1. For this purpose, it is more convenient to introduce the decorated (*x*, *q*)-series

106 $$\begin{aligned} \begin{aligned} \mathcal {C}_m(x,q)&= C_m(x,q),\\ \mathcal {A}_m(x,q)&=\frac{(qx^2;q)_\infty }{\theta (-q^{1/2}x,q)}A_m(x,q),\\ \mathcal {B}_m(x,q)&=x\frac{(qx^{-2};q)_\infty }{\theta (-q^{1/2}x^{-1},q)}B_m(x,q), \end{aligned} \end{aligned}$$

where

107 $$\begin{aligned} \theta (x,q) = (-q^{1/2}x;q)_\infty (-q^{1/2}x^{-1};q)_\infty . \end{aligned}$$

They satisfy the recursion relation in *m*

108 $$\begin{aligned} \mathcal {F}_{m+1}(x,q) + (q^m-x-x^{-1})\mathcal {F}_m(x,q) +\mathcal {F}_{m-1}(x,q) = \delta _{\mathcal {C}}, \end{aligned}$$

where $\mathcal {F} = \mathcal {A},\mathcal {B},\mathcal {C}$ and $\delta _{\mathcal {C}}$ means the inhomogeneous term is only present for $\mathcal {F} = \mathcal {C}$. In addition, $\mathcal {A}_m(x,q), \mathcal {B}_m(x,q)$ as well as $\textsf{C}_m(x,q) = (1-x)\mathcal {C}_m(x,q)$ also satisfy the *q*-difference equations with respect to *x*

109 $$\begin{aligned} &  q^m x^2(1-q^{-1}x^2)\textsf{F}_m(qx,q) + q^mx^2(1-qx^2)\textsf{F}_m(q^{-1}x,q)\nonumber \\ &  \quad -(1-x)(1+x)(1+x^4-q^m(x+x^3)-(q^{-1}+q)x^2)\textsf{F}_m(x,q) \nonumber \\ &  \quad =\delta _{\textsf{C}}x(1+x)(1-qx^2)(1-q^{-1}x^2), \end{aligned}$$

where $\textsf{F} = \mathcal {A},\mathcal {B},\textsf{C}$ and $\delta _{\textsf{C}}$ means the inhomogeneous term is only present for $\textsf{F} = \textsf{C}$. Note that when $m=0$, (109) reduces to the inhomogeneous ${\widehat{A}}$-polynomial in [GGMn23]. The associated decorated matrix $\mathcal {J}(x,q)$ is given by

110 $$\begin{aligned} {\mathcal {J}}_{m}(x,q)&= \begin{pmatrix} 1 & {\mathcal {C}}_{m}(x,q) & {\mathcal {C}}_{m+1}(x,q)\\ 0 & {\mathcal {A}}_{m}(x,q) & {\mathcal {A}}_{m+1}(x,q)\\ 0 & {\mathcal {B}}_{m}(x,q) & {\mathcal {B}}_{m+1}(x,q) \end{pmatrix}\nonumber \\&=\begin{pmatrix} 1 & 0 & 0\\ 0 & \frac{(qx^{2};q)_{\infty }}{\theta (-q^{1/2}x;q)^{2}} & 0\\ 0 & 0 & x\frac{(qx^{-2};q)_{\infty }}{\theta (-q^{1/2}x^{-1};q)^{2}} \end{pmatrix} {\textbf{J}}_{m}(x,q) \end{aligned}$$

and it has

111 $$\begin{aligned} \det \mathcal {J}(x,q):= \det \mathcal {J}_m(x,q) = \theta (-q^{-1/2}x^2,q) \theta (-q^{1/2}x;q)^{-2}\theta (-q^{1/2}x^{-1},q)^{-2}.\nonumber \\ \end{aligned}$$

We will focus on the vector $\mathcal {B}(x,q)$ of (*x*, *q*)-series

112 $$\begin{aligned} \mathcal {B}(x,q) = \begin{pmatrix} \mathcal {C}_0(x,q)\\ \mathcal {A}_0(x,q)\\ \mathcal {B}_0(x,q) \end{pmatrix}, \end{aligned}$$

which is defined for $|q| \ne 1$ and satisfies by

113 $$\begin{aligned} \mathcal {B}(x,q^{-1}) = \begin{pmatrix} 1& 0& 0\\ 0& 0& x \det \mathcal {J}(x,q)^{-1}\\ 0& -x\det \mathcal {J}(x,q)^{-1}& 0 \end{pmatrix} \mathcal {B}(x,q). \end{aligned}$$

We will write

114 $$\begin{aligned} q=e^{2\pi {\textrm{i}}\tau }, \qquad x=e^u \end{aligned}$$

and we will show that the asymptotic expansion of $\mathcal {B}(x,q)$ in the limit $\tau \rightarrow 0$ is related to the vector $\Phi (x,\tau )$ of $(x,\tau )$ asymptotic series

115 $$\begin{aligned} \Phi (x,\tau ) = \begin{pmatrix} \Phi ^{(\sigma _0)}(x,\tau )\\ \Phi ^{(\sigma _1)}(x,\tau )\\ \Phi ^{(\sigma _2)}(x,\tau ) \end{pmatrix} \end{aligned}$$

with corrections given by $\mathcal {B}({\tilde{x}},{\tilde{q}})$ where

116 $$\begin{aligned} {\tilde{q}}=e^{-2\pi {\textrm{i}}/\tau }, \qquad {\tilde{x}}=e^{u/\tau } . \end{aligned}$$

**Fig. 4:**
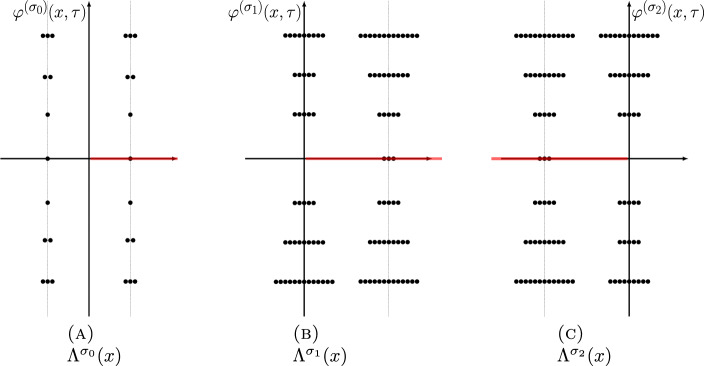
Singularities of the Borel transforms of $\varphi ^{(\sigma _j)}(x,\tau )$ for $j=0,1,2$ of the knot $\textbf{4}_1$. Here we take small and real *x*. Red lines are some Stokes rays

The asymptotic series $\Phi (x,\tau )$ can be resummed by Borel resummation. As we have explained in Sect. 2.4 the value of the Borel resummation depends on the singularities of the Borel transform of $\Phi (x,\tau )$. The positions of these singular points, denoted collectively as $\Lambda (x)$, are smooth functions of *x*, and in the limit $x=1$ they are equal to $\Lambda$ defined in (34). When *x* is near 1, which is the regime we will be interested in, each singular point $\iota _{i,j}^{(k)}$ in $\Lambda$ splits to a finite set of points located at $\iota _{i,j}^{(k,\ell )}:=\iota _{i,j}^{(k)}+\ell \log (x)$, where $\ell$ takes value in a finite subset of ${\mathbb {Z}}$ that depends on *i*, *j*, *k*. These singular points are aligned on a line and are apart from each other by a distance $\log (x)$. We illustrate this schematically in Fig. 4. The complex plane of $\tau$ is divided to infinitely many cones by rays passing through these singular points, and the Borel resummation of $\Phi (x,\tau )$, denoted by $s_R(\Phi )(x,\tau )$, is only well-defined within a cone *R*.

We conjecture that the asymptotic expansion in the limit $q\rightarrow 1$ of the vector of (*x*, *q*)-series $\mathcal {B}(x,q)$ can be expressed in terms of $s_R(\Phi )(x,\tau )$. Furthermore, in each cone, the asymptotic expansion can be upgraded to exact identities between $\mathcal {B}(x,q)$ and linear transformation of Borel resummation of $\Phi (x,\tau )$ up to exponentially small corrections characterised by ${\tilde{q}}$ and ${\tilde{x}}= \exp (\frac{\log x}{\tau })$.

#### Conjecture 14

For every $x \sim 1$, every cone $R \subset \mathbb {C}\setminus \Lambda (x)$ and every $\tau \in R$ we have

117 $$\begin{aligned} \Delta '(x,\tau ) \mathcal {B}(x,q) = M_R({\tilde{x}},{\tilde{q}}) \Delta (x,\tau )s_R(\Phi )(x,\tau ), \end{aligned}$$

where

118 $$\begin{aligned} \begin{aligned} \Delta '(x,\tau )&=\textrm{diag}(\tau ^{1/2}\tfrac{x^{1/2}-x^{-1/2}}{{\tilde{x}}^{1/2}-{\tilde{x}}^{-1/2}}, ({\tilde{x}}/x)^{1/2}{\textrm{e}}^{\frac{3\pi {\textrm{i}}}{4}-\frac{\pi {\textrm{i}}}{4}(\tau +\tau ^{-1})}, ({\tilde{x}}/x)^{1/2}{\textrm{e}}^{\frac{3\pi {\textrm{i}}}{4}-\frac{\pi {\textrm{i}}}{4}(\tau +\tau ^{-1})}),\\ \Delta (x,\tau )&= \textrm{diag}(\tau ^{1/2} \tfrac{x^{1/2}-x^{-1/2}}{{\tilde{x}}^{1/2}-{\tilde{x}}^{-1/2}},1,1), \end{aligned} \end{aligned}$$

and $M_R({\tilde{x}},{\tilde{q}})$ is a $3\times 3$ matrix of ${\tilde{q}}$ (resp., ${\tilde{q}}^{-1}$)-series if $\textrm{Im}\tau >0$ (resp., $\textrm{Im}\tau <0$) with coefficients in ${\mathbb {Z}}[{\tilde{x}}^{\pm 1}]$ that depend on *R*.

**Fig. 5:**
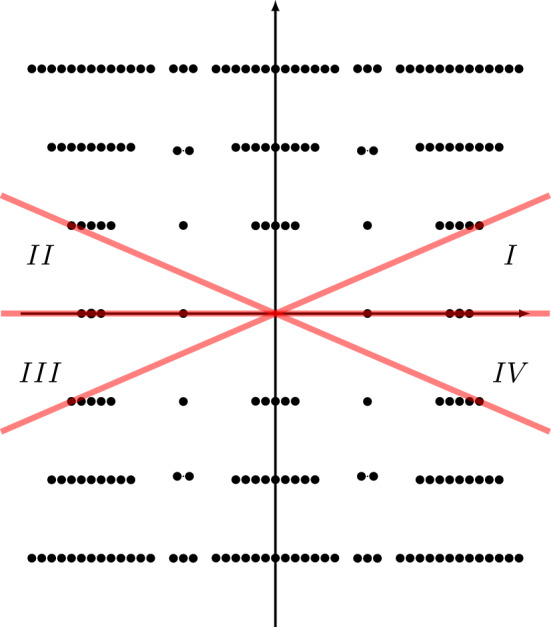
Stokes rays and cones in the $\tau$-plane for the 3-vector $\Phi (x,\tau )$ of asymptotic series of the knot $\textbf{4}_1$. Here we take small and real *x*

To illustrate examples of $M_R({\tilde{x}},{\tilde{q}})$, we pick four of these cones, located slightly above and below the positive or negative real axis, labeled in counterclockwise direction by *I*, *II*, *III*, *IV*, cf. Fig. 5.

#### Conjecture 15

Equation (117) holds in the cones $R=I,II,III,IV$ where the matrices $M_R({\tilde{x}},{\tilde{q}})$ are given in terms of $\mathcal {J}_{-1}({\tilde{x}},{\tilde{q}})$ as follows

119a $$\begin{aligned} M_I({\tilde{x}},{\tilde{q}})&= \mathcal {J}_{-1}({\tilde{x}},{\tilde{q}}) \begin{pmatrix} 1& 0& 0\\ 0& 0& -1\\ 0& 1& -1 \end{pmatrix}, \quad |{\tilde{q}}|<1,\end{aligned}$$

119b $$\begin{aligned} M_{II}({\tilde{x}},{\tilde{q}})&= \begin{pmatrix} 1& 0& 0\\ 0& 0& 1\\ 0& 1& 0 \end{pmatrix} \mathcal {J}_{-1}({\tilde{x}},{\tilde{q}}) \begin{pmatrix} 1& 0& 0\\ 0& 1& 0\\ 0& 1& -1 \end{pmatrix}, \quad |{\tilde{q}}|<1,\end{aligned}$$

119c $$\begin{aligned} M_{III}({\tilde{x}},{\tilde{q}})&= \begin{pmatrix} 1& 0& 0\\ 0& 0& -1\\ 0& -1& 0 \end{pmatrix} \mathcal {J}_{-1}({\tilde{x}},{\tilde{q}}) \begin{pmatrix} 1& {\tilde{x}}^{-1}& 0\\ 0& -1& 0\\ 0& {\tilde{x}}+{\tilde{x}}^{-1}& 1 \end{pmatrix}, \quad |{\tilde{q}}|>1,\end{aligned}$$

119d $$\begin{aligned} M_{IV}({\tilde{x}},{\tilde{q}})&= \mathcal {J}_{-1}({\tilde{x}},{\tilde{q}}) \begin{pmatrix} 1& 0& {\tilde{x}}^{-1}\\ 0& 0& -1\\ 0& 1& {\tilde{x}}+{\tilde{x}}^{-1} \end{pmatrix}, \quad |{\tilde{q}}|>1. \end{aligned}$$

#### Remark 16

It is sometimes stated in the literature that the Gukov–Manolescu series is obtained by “resumming” the perturbative series $\Phi ^{(\sigma _0)}(x, \tau )$ associated to the trivial connection, although it is not always clear what “resumming” means in that context. The above conjecture shows that, generically, $\mathcal {C}_0(x,q)$ involves the Borel resummation of all perturbative series $\Phi ^{(\sigma _j)}(x, \tau )$, $j=0,1,2$, as well as non-perturbative corrections in ${\tilde{q}}, {\tilde{x}}$.

We now discuss the Stokes automorphism of the Borel resummation $s_R(\Phi )(x,\tau )$. The discussion is similar to the one in Sect. 2.4. To any singular point of the Borel transform of $\Phi (x,\tau )$ locatd at $\iota _{i,j}^{(k,\ell )}$, we can associate a local Stokes matrix

120 $$\begin{aligned} \mathfrak {S}_{\iota _{i,j}^{(k,\ell )}} = I + \mathcal {S}_{i,j}^{(k,\ell )}{\tilde{q}}^k{\tilde{x}}^\ell E_{i,j}, \quad \mathcal {S}_{i,j}^{(k,\ell )}\in {\mathbb {Z}}, \end{aligned}$$

where $E_{i,j}$ is the elementary matrix with (*i*, *j*)-entry 1 ($i,j=0,1,2$) and all other entries zero, and $\mathcal {S}_{i,j}^{(k,\ell )}$ is the Stokes constant. Let us again assume the locality condition. Then for any ray of angle $\theta$, the Borel resummations of $\Phi (x,\tau )$ with $\tau$ whose argument is raised slightly above $\theta$ ($\theta _+$) or sightly below ($\theta _-$) are related by the following formula of Stokes automorphism

121 $$\begin{aligned} \Delta (x,\tau )s_{\theta _+}(\Phi )(x,\tau ) = \mathfrak {S}_{\theta }({\tilde{x}},{\tilde{q}}) \Delta (x,\tau )s_{\theta _-}(\Phi )(x,\tau ),\quad \mathfrak {S}_\theta ({\tilde{x}},{\tilde{q}}) = \prod _{\arg \iota =\theta }\mathfrak {S}_\iota ({\tilde{x}},{\tilde{q}}).\nonumber \\ \end{aligned}$$

Because of the locality condition, we don’t have to worry about the order of product of local Stokes matrices.

In addition, given two rays $\rho _{\theta ^+}$ and $\rho _{\theta ^-}$ whose arguments satisfy $0<\theta ^+-\theta ^-\le \pi$, we define the global Stokes matrix $\mathfrak {S}_{\theta ^-\rightarrow \theta ^+}({\tilde{x}},{\tilde{q}})$ by

122 $$\begin{aligned} \Delta (x,\tau )s_{\theta ^+}(\Phi )(x,\tau ) = \mathfrak {S}_{\theta ^-\rightarrow \theta ^+}({\tilde{x}},{\tilde{q}}) \Delta (x,\tau )s_{\theta ^-}(\Phi )(x,\tau ), \end{aligned}$$

where both sides are analytically continued smoothly to the same value of $\tau$. The global Stokes matrix $\mathfrak {S}_{\theta ^-\rightarrow \theta ^+}({\tilde{x}},{\tilde{q}})$ satisfies the factorisation property [GGMn21, GGMn23]

123 $$\begin{aligned} \mathfrak {S}_{\theta ^-\rightarrow \theta ^+}({\tilde{x}},{\tilde{q}}) = \prod _{\theta ^-<\theta <\theta ^+}^{\leftarrow } \mathfrak {S}_{\theta }({\tilde{x}},{\tilde{q}}), \end{aligned}$$

where the ordered product is taken over all the local Stokes matrices whose arguments are sandwiched between $\theta ^-,\theta ^+$ and they are ordered with rising arguments from right to left.

Given (117) with explicit values of $M_R({\tilde{x}},{\tilde{q}})$ for $R=I,II,III,IV$, in general we can calculate the global Stokes matrix via

124 $$\begin{aligned} \mathfrak {S}_{R\rightarrow R'}({\tilde{x}},{\tilde{q}}) = M_{R'}({\tilde{x}},{\tilde{q}})^{-1} \cdot M_{R}({\tilde{x}},{\tilde{q}}). \end{aligned}$$

For instance, we find the global Stokes matrix from cone *I* anti-clockwise to cone *II* is

125 $$\begin{aligned} \mathfrak {S}_{I\rightarrow II}({\tilde{x}},{\tilde{q}}) = \begin{pmatrix} 1& 0& 0\\ 0& 1& 0\\ 0& 1& -1 \end{pmatrix} \mathcal {J}_{-1}({\tilde{x}},{\tilde{q}})^{-1} \begin{pmatrix} 1& 0& 0\\ 0& 0& 1\\ 0& 1& 0 \end{pmatrix} \mathcal {J}_{-1}({\tilde{x}},{\tilde{q}}) \begin{pmatrix} 1& 0& 0\\ 0& 0& -1\\ 0& 1& -1 \end{pmatrix},\quad |{\tilde{q}}|<1.\nonumber \\ \end{aligned}$$

This Stokes matrix has the block upper triangular form

126 $$\begin{aligned} \begin{pmatrix} 1& *& *\\ 0& *& *\\ 0& *& * \end{pmatrix}. \end{aligned}$$

One can verify that the $2\times 2$ submatrix of $\mathfrak {S}_{I\rightarrow II}({\tilde{x}},{\tilde{q}})$ in the bottom right is the Stokes matrix in [GGMn21]. In addition we can also extract Stokes constants $\mathcal {S}_{0,j}^{(k,\ell )}$ ($j=1,2, k=1,2,\ldots$) responsible for Stokes automorphisms into $\Phi ^{(\sigma _0)}(x,\tau )$ from Borel singularities in the upper half plane, and collect them in the generating series

127 $$\begin{aligned} \textsf{S}^+_{0,j}({\tilde{x}},{\tilde{q}}) = \sum _{k=1}^\infty \sum _{\ell } \mathcal {S}_{0,j}^{(k,\ell )}{\tilde{x}}^\ell {\tilde{q}}^k,\quad j=1,2. \end{aligned}$$

We find

128 $$\begin{aligned} \textsf{S}_{0,1}^+({\tilde{x}},{\tilde{q}})&= \textsf{S}_{0,2}^+({\tilde{x}},{\tilde{q}}) = {\tilde{x}}^{-1}\left( -\mathcal {C}_{-1}({\tilde{x}},{\tilde{q}})+\mathcal {C}_0({\tilde{x}},{\tilde{q}}) \frac{\mathcal {A}_{-1}({\tilde{x}},{\tilde{q}})+\mathcal {B}_{-1}({\tilde{x}},{\tilde{q}})}{\mathcal {A}_0({\tilde{x}},{\tilde{q}})+\mathcal {B}_0({\tilde{x}},{\tilde{q}})} \right) \nonumber \\&= -{\tilde{q}}-({\tilde{x}}+{\tilde{x}}^{-1}){\tilde{q}}^2 -({\tilde{x}}^2+1+{\tilde{x}}^{-2}){\tilde{q}}^3+\ldots . \end{aligned}$$

Similarly, we find the global Stokes matrix from cone *III* anti-clockwise to cone *IV* is

129 $$\begin{aligned} \mathfrak {S}_{III\rightarrow IV}({\tilde{x}},{\tilde{q}})= &  \begin{pmatrix} 1& 0& 0\\ 0& -1& 1\\ 0& 1& 0 \end{pmatrix}\cdot \mathcal {J}_{-1}({\tilde{x}},{\tilde{q}}^{-1})^{-1}\cdot \begin{pmatrix} 1& 0& 0\\ 0& 0& 1\\ 0& 1& 0 \end{pmatrix} \cdot \mathcal {J}_{-1}({\tilde{x}},{\tilde{q}}^{-1})\nonumber \\ &  \cdot \begin{pmatrix} 1& 0& 0\\ 0& 1& 0\\ 0& 1& 1 \end{pmatrix},\quad |{\tilde{q}}|>1. \end{aligned}$$

It also has the form as (126). This, together with the same phenomenon in the upper half plane, implies that $\Phi ^{(s_j)}(x,\tau )$ ($j=1,2$) form a minimal resurgent structure. The $2\times 2$ submatrix of $\mathfrak {S}_{III\rightarrow IV}({\tilde{x}},{\tilde{q}})$ in the bottom right is identical to the Stokes matrix given in [GGMn21]. We also extract Stokes constants $\mathcal {S}_{0,j}^{(k,\ell )}$ ($j=1,2$, $k=-1,-2,\ldots$) responsible for Stokes automorphisms into $\Phi ^{(\sigma _0)}(x,\tau )$ from Borel singularities in the lower half plane, and collect them in the generating series

130 $$\begin{aligned} \textsf{S}^-_{0,j}({\tilde{x}},{\tilde{q}}) = \sum _{k=-1}^{-\infty }\sum _{\ell } \mathcal {S}_{0,j}^{(k,\ell )}{\tilde{x}}^\ell {\tilde{q}}^k,\quad j=1,2. \end{aligned}$$

And we find

131 $$\begin{aligned} \textsf{S}_{0,2}^-({\tilde{x}},{\tilde{q}}) = -\textsf{S}_{0,1}^-({\tilde{x}},{\tilde{q}}) = \textsf{S}_{0,1}^+({\tilde{x}},{\tilde{q}}^{-1}). \end{aligned}$$

We can also use (124) to compute the global Stokes matrix $\mathfrak {S}_{IV\rightarrow I}({\tilde{q}})$ and we find

132 $$\begin{aligned} \mathfrak {S}_{IV\rightarrow I} = \begin{pmatrix} 1& 0& 1\\ 0& 1& {\tilde{x}}+1+{\tilde{x}}^{-1}\\ 0& 0& 1 \end{pmatrix}. \end{aligned}$$

Note that this can be identified as $\mathfrak {S}_{0}$, associated to the ray $\rho _0$, and it can be factorised as

133 $$\begin{aligned} \mathfrak {S}_{0} = \mathfrak {S}_{\iota _{0,2}}\mathfrak {S}_{\iota _{1,2}},\quad \mathfrak {S}_{\iota _{0,2}} = \begin{pmatrix} 1& 0& 1\\ 0& 1& 0\\ 0& 0& 1 \end{pmatrix},\quad \mathfrak {S}_{\iota _{1,2}} = \begin{pmatrix} 1& 0& 0\\ 0& 1& {\tilde{x}}+1+{\tilde{x}}^{-1}\\ 0& 0& 1 \end{pmatrix}. \end{aligned}$$

Since the local Stokes matrices $\mathfrak {S}_{\iota _{0,2}}$ and $\mathfrak {S}_{\iota _{1,2}}$ commute, the locality condition is satisfied. We read off the Stoke discontinuity formulas

134 $$\begin{aligned} \begin{aligned} \text {disc}_0 \Phi ^{(0)}(x,\tau )&= \frac{{\tilde{x}}^{1/2}-{\tilde{x}}^{-1/2}}{x^{1/2}-x^{-1/2}} \tau ^{-1/2}s(\Phi ^{(s_2)})(x,\tau ), \\ \text {disc}_0 \Phi ^{(1)}(x,\tau )&= ({\tilde{x}}+1+{\tilde{x}}^{-1})s(\Phi ^{(s_2)})(x,\tau ) . \end{aligned} \end{aligned}$$

They reduce properly to (56) in the $x\rightarrow 1$ limit, and the second identity has already appeared in [GGMn21].

Finally, in order to compute the global Stokes matrix $\mathfrak {S}_{II\rightarrow III}({\tilde{q}})$, we need to take into account that the odd powers of $\tau ^{1/2}$ on both sides of (117) give rise to additional $-1$ factors when one crosses the branch cut at the negative real axis, and (124) should be modified by

135 $$\begin{aligned} \mathfrak {S}_{II\rightarrow III}({\tilde{q}}) = \textrm{diag}(-1,1,1) M_{III}({\tilde{q}})^{-1}\textrm{diag}(-1,1,1) M_{II}({\tilde{q}}), \end{aligned}$$

and we find

136 $$\begin{aligned} \mathfrak {S}_{II\rightarrow III} = \begin{pmatrix} 1& 1& 0\\ 0& 1& 0\\ 0& -{\tilde{x}}-1-{\tilde{x}}^{-1}& 1 \end{pmatrix}. \end{aligned}$$

Similarly this can be identified as $\mathfrak {S}_{\pi }$ associated to the ray $\rho _{\pi }$ and it can be factorised as

137 $$\begin{aligned} \mathfrak {S}_{\pi } = \mathfrak {S}_{\iota _{0,1}}\mathfrak {S}_{\iota _{2,1}},\quad \mathfrak {S}_{\iota _{0,1}} = \begin{pmatrix} 1& 1& 0\\ 0& 1& 0\\ 0& 0& 1 \end{pmatrix},\quad \mathfrak {S}_{\iota _{2,1}} = \begin{pmatrix} 1& 0& 0\\ 0& 1& 0\\ 0& -{\tilde{x}}-1-{\tilde{x}}^{-1}& 1 \end{pmatrix}. \end{aligned}$$

Note that the local Stokes matrices $\mathfrak {S}_{\iota _{0,1}}$ and $\mathfrak {S}_{\iota _{2,1}}$ also commute. We read off the Stokes discontinuity formulas

138 $$\begin{aligned} \text {disc}_\pi \Phi ^{(0)}(x,\tau )&= \frac{{\tilde{x}}^{1/2}-{\tilde{x}}^{-1/2}}{x^{1/2}-x^{-1/2}} \tau ^{-1/2}s(\Phi ^{(s_1)})(x,\tau ), \end{aligned}$$

139 $$\begin{aligned} \text {disc}_\pi \Phi ^{(2)}(x,\tau )&= -({\tilde{x}}+1+{\tilde{x}}^{-1})s(\Phi ^{(s_1)})(x,\tau ). \end{aligned}$$

They reduce properly to (61) in the $x\rightarrow 1$ limit, and the second identity has already appeared in [GGMn21].

### $(u,\tau )$ state-integrals

In parallel to the discussion in Sects. 2.6 and 2.7, we now introduce a new state-integral which depends on $\tau$, but also on a variable *u*. Let us consider the state-integral

140 $$\begin{aligned} \mathcal {Z}_{\mathcal {B}}(u,\tau ) = -\frac{{\textrm{i}}}{2{\textsf{b}}}\frac{\sinh (\pi {\textsf{b}}^{-1} u)}{\sinh (\pi {\textsf{b}}u)} \int _{{\mathcal {B}}} \tanh (\pi {\textsf{b}}^{-1}v) \frac{\Phi _{\textsf{b}}(-v+\frac{{\textrm{i}}}{2}{\textsf{b}}^{-1}+u)}{\Phi _{\textsf{b}}(v-\frac{{\textrm{i}}}{2}{\textsf{b}}^{-1}+u)} {\textrm{e}}^{2\pi {\textrm{i}}u(v-\frac{{\textrm{i}}}{2}{\textsf{b}}^{-1})} {\textrm{d}}v,\nonumber \\ \end{aligned}$$

where the contour of integral ${\mathcal {B}}$ is not specified yet. The integrand reduces to that of (69) in the limit $u\rightarrow 0$. For generic ${\textsf{b}}^2\in {\mathbb {C}}'$ so that ${{\,\textrm{Re}\,}}{\textsf{b}}>0$, the integrand has the following poles and zeros

141 $$\begin{aligned} \begin{aligned}&\text {Poles}:\; \left\{ \pm {\textrm{i}}{\textsf{b}}\left( \frac{1}{2}+m\right) ,\; \pm u -{\textrm{i}}{\textsf{b}}\left( \frac{1}{2}+m\right) -{\textrm{i}}{\textsf{b}}^{-1}n\;\big |\; m,n=0,1,2,\ldots \right\} \\&\text {Zeros}:\; \left\{ \pm u +{\textrm{i}}{\textsf{b}}\left( \frac{1}{2}+m \right) +{\textrm{i}}{\textsf{b}}^{-1}(1+n)\;\big |\; m,n=0,1,2,\ldots \right\} . \end{aligned} \end{aligned}$$

We can choose for the integral the contour $\mathcal {A}_N$ in the upper half plane that wraps the following poles, as in the left panel of Fig. 3,

142 $$\begin{aligned} v_m = {\textrm{i}}{\textsf{b}}\left( \frac{1}{2}+m\right) ,\quad m=0,1,2,\ldots ,N-1. \end{aligned}$$

By summing over the residues of these poles, the integral evaluates as follows

143 $$\begin{aligned} \mathcal {Z}_{\mathcal {A}_N}(u_{{\textsf{b}}},\tau ) = \sum _{n=0}^{N-1}(-1)^n q^{-n(n+1)/2}(qx;q)_n(qx^{-1};q)_n,\qquad x= {\textrm{e}}^{u},\, q={\textrm{e}}^{2\pi {\textrm{i}}\tau } ,\nonumber \\ \end{aligned}$$

where we defined $u_{{\textsf{b}}}=u/(2\pi {\textsf{b}})$, as in [GGMn23, Eq. (2)]. When $x = q^N$ this is none other than the colored Jones polynomial of the knot $\textbf{4}_1$

144 $$\begin{aligned} \mathcal {Z}_{\mathcal {A}_N}({\textrm{i}}N{\textsf{b}},{\textsf{b}}^2) = J_N^{\textbf{4}_1}(q) = \sum _{n=0}^{N-1}(-1)^n q^{-n(n+1)/2}(q^{1+N};q)_n(q^{1-N};q)_n. \end{aligned}$$

Alternatively, we can choose for the integral the contour $\mathcal {C}$ as in the right panel of Fig. 3, which is asymptotic to a horizontal line slightly below ${{\,\textrm{Im}\,}}(v) = {{\,\textrm{Re}\,}}({\textsf{b}}^{-1})$, but deformed near the origin in such a way that all the poles

145 $$\begin{aligned} v^{\pm }_{m,n} = \pm u -{\textrm{i}}{\textsf{b}}\left( \frac{1}{2}+m\right) - {\textrm{i}}{\textsf{b}}^{-1}n,\quad m,n=0,1,2,\ldots \end{aligned}$$

are below the contour $\mathcal {C}$. Let $\mathcal {Z}(u,\tau ):= \mathcal {Z}_{\mathcal {C}}(u,\tau )$ denote the corresponding state-integral. Similar to the discussion in Sect. 2.6, as the integrand has non-trivial contributions from infinity in the upper half plane, the two integrals $\mathcal {Z}_{\mathcal {A}_N}(u,\tau )$ and $\mathcal {Z}(u,\tau )$ are different. On the other hand, since the integrand does have vanishing contributions from infinity in the lower half plane, we can smoothly deform the contour $\mathcal {C}$ downwards so that $\mathcal {Z}(u,\tau )$ can be evaluated by summing over residues at the poles $v^{\pm }_{m,n}$, and we find

146 $$\begin{aligned} \mathcal {Z}(u,\tau )&= \mathcal {C}_0(x,q) +\frac{{\textrm{e}}^{\frac{3\pi {\textrm{i}}}{4}-\frac{\pi {\textrm{i}}}{4}(\tau +\tau ^{-1})}}{\tau ^{1/2}}\frac{{\tilde{x}}^{-1}-1}{1-x} \mathcal {A}_0(x,q)\left( L\mathcal {A}_{0}({\tilde{x}},{\tilde{q}}^{-1}) +\frac{1}{2}\mathcal {A}_{0}({\tilde{x}},{\tilde{q}}^{-1})\right) \nonumber \\&\quad +\frac{{\textrm{e}}^{\frac{3\pi {\textrm{i}}}{4}-\frac{\pi {\textrm{i}}}{4}(\tau +\tau ^{-1})}}{\tau ^{1/2}}\frac{{\tilde{x}}^{-1}-1}{1-x} \mathcal {B}_0(x,q)\left( L\mathcal {B}_{0}({\tilde{x}},{\tilde{q}}^{-1}) +\frac{1}{2}\mathcal {B}_{0}({\tilde{x}},{\tilde{q}}^{-1}) \right) , \end{aligned}$$

where $L\mathcal {A}_{\mu }(x,q),L\mathcal {B}_{\mu }(x,q)$ are defined as in (100) with Roman letters *A*, *B*, *C* replaced by caligraphic letters $\mathcal {A},\mathcal {B},\mathcal {C}$. As mentioned above, the change of integration contour implements the Habiro inversion of [Par]: the integration over $\mathcal {A}_N$ gives the Habiro series (144), while the integration over $\mathcal {C}$ involves $\mathcal {C}_0(x,q)$, which was interpreted in [Par] as an inverted Habiro series. This contribution comes from the poles $-v_m$ in the lower half-plane.

The integral $\mathcal {Z}(u,\tau )$ can also be identified with the Borel resummation of the perturbative series $\Phi ^{(\sigma _j)}(x;\tau )$ for $j=0,1,2$. By inverting the matrix $M_R({\tilde{x}},{\tilde{q}})$ in (117), we can also express the Borel resummation $s_R(\Phi )(x,\tau )$ in any cone *R* in terms of combinations of (*x*, *q*)- and $({\tilde{x}},{\tilde{q}})$-series, and they can be then compared with the right hand side of (146). For instance, in the cones *I* and *IV* respectively, we find

147a $$\begin{aligned} \mathcal {Z}(u,\tau )&= s_I(\Phi ^{(\sigma _0)})(x;\tau ) - \frac{{\tilde{x}}^{1/2}-{\tilde{x}}^{-1/2}}{2(x^{1/2}-x^{-1/2})}\tau ^{-1/2}s_I(\Phi ^{(\sigma _2)})(x;\tau ), \end{aligned}$$

147b $$\begin{aligned}&= s_{IV}(\Phi ^{(\sigma _0)})(x;\tau ) + \frac{{\tilde{x}}^{1/2}-{\tilde{x}}^{-1/2}}{2(x^{1/2}-x^{-1/2})} \tau ^{-1/2}s_{IV}(\Phi ^{(\sigma _2)})(x;\tau ). \end{aligned}$$

This also implies that for positive real $\tau$,

148 $$\begin{aligned} \mathcal {Z}(u,\tau ) =s_{\text {med}}(\Phi ^{(\sigma _0)})(x;\tau ) . \end{aligned}$$

Finally, we can introduce the descendants of the integral $\mathcal {Z}(u,\tau )$ as follows

149 $$\begin{aligned} \mathcal {Z}_{\lambda ,\mu }(u,\tau )= &  -\frac{{\textrm{i}}}{2{\textsf{b}}}\frac{\sinh (\pi {\textsf{b}}^{-1} u)}{\sinh (\pi {\textsf{b}}u)} \nonumber \\ &  \int _{\mathcal {C}} \tanh (\pi {\textsf{b}}^{-1}v) \frac{\Phi _{\textsf{b}}(-v+\frac{{\textrm{i}}}{2}{\textsf{b}}^{-1}+u)}{\Phi _{\textsf{b}}(v-\frac{{\textrm{i}}}{2}{\textsf{b}}^{-1}+u)} {\textrm{e}}^{2\pi {\textrm{i}}u(v-\frac{{\textrm{i}}}{2}{\textsf{b}}^{-1})-2\pi (\lambda {\textsf{b}}-\mu {\textsf{b}}^{-1})v} {\textrm{d}}v.\nonumber \\ \end{aligned}$$

The integrand has the same poles and zeros as in (141). To ensure convergence, the contour $\mathcal {C}$ needs slight modification: it is asymptotic to a horizontal line slightly below ${{\,\textrm{Im}\,}}(v) = \frac{1}{2}{{\,\textrm{Re}\,}}({\textsf{b}}^{-1}) -|{{\,\textrm{Re}\,}}(\lambda {\textsf{b}}-\mu {\textsf{b}}^{-1})|$, and it is deformed near the origin in such a way that all the poles (145) are below the contour $\mathcal {C}$. Similarly, by smoothly deforming the contour downwards we can evaluate this integral by summing up residues of all the poles in the lower half plane, and we find

150 $$\begin{aligned} \mathcal {Z}_{\lambda ,\mu }(u,\tau )&= (-1)^\mu q^{\lambda /2}\left( \mathcal {C}_\lambda (x,q)+\frac{{\textrm{e}}^{\frac{3\pi {\textrm{i}}}{4}-\frac{\pi {\textrm{i}}}{4}(\tau +\tau ^{-1})}}{\tau ^{1/2}}\frac{{\tilde{x}}^{-1}-1}{1-x} \mathcal {A}_\lambda (x,q)\right. \nonumber \\&\quad \left( L\mathcal {A}_{-\mu }({\tilde{x}},{\tilde{q}}^{-1}) +\frac{1}{2}\mathcal {A}_{-\mu }({\tilde{x}},{\tilde{q}}^{-1})\right) \nonumber \\&\quad +\frac{{\textrm{e}}^{\frac{3\pi {\textrm{i}}}{4}-\frac{\pi {\textrm{i}}}{4}(\tau +\tau ^{-1})}}{\tau ^{1/2}}\frac{{\tilde{x}}^{-1}-1}{1-x} \mathcal {B}_\lambda (x,q)\nonumber \\&\quad \left. \left( L\mathcal {B}_{-\mu }({\tilde{x}},{\tilde{q}}^{-1}) +\frac{1}{2}\mathcal {B}_{-\mu }({\tilde{x}},{\tilde{q}}^{-1})\right) \right) . \end{aligned}$$

### An analytic extension of the colored Jones polynomial

In this section we discuss a Borel resummation formula for the colored Jones polynomial of the $\textbf{4}_1$ knot. The latter is defined by

151 $$\begin{aligned} J^{\textbf{4}_1}_N(q) = \sum _{k=0}^{N-1}(-1)^k q^{-k(k+1)/2}(q^{1+N};q)_k(q^{1-N};q)_k . \end{aligned}$$

Let $u \sim 0$ be in a small neighborhood of the origin in the complex plane. It is related to $x = q^N$ and $\tau$ by

152 $$\begin{aligned} x = {\textrm{e}}^{u},\quad \tau = \frac{u}{2\pi {\textrm{i}}N}+\frac{1}{N}. \end{aligned}$$

Then *u* is near 0, then *x* is close to 1, which is the regime that we studied in Sect. 3.3, and $\tau$ is close to 1/*N*. Note that $N\tau = 1+\frac{u}{2\pi {\textrm{i}}}$ is the analogue of *n*/*k* in [Guk05], and here we are considering a deformation from the case of $n/k=1$.

Experimentally, we found that in cones *I* and *IV* respectively, we have

153a $$\begin{aligned} J^{\textbf{4}_1}_N(q)&= s_{I}(\Phi ^{(\sigma _0)})(x;\tau ) +\frac{{\tilde{x}}^{1/2}-{\tilde{x}}^{-1/2}}{x^{1/2}-x^{-1/2}} \tau ^{-1/2}s_{I}(\Phi ^{(\sigma _1)})(x;\tau ) \nonumber \\&\quad -(1+{\tilde{x}})\frac{{\tilde{x}}^{1/2}-{\tilde{x}}^{-1/2}}{x^{1/2}-x^{-1/2}}\tau ^{-1/2} s_{I}(\Phi ^{(\sigma _2)})(x;\tau ) \end{aligned}$$

153b $$\begin{aligned}&= s_{IV}(\Phi ^{(\sigma _0)})(x;\tau ) +\frac{{\tilde{x}}^{1/2}-{\tilde{x}}^{-1/2}}{x^{1/2}-x^{-1/2}} \tau ^{-1/2}s_{IV}(\Phi ^{(\sigma _1)})(x;\tau )\nonumber \\&\quad +(1+{\tilde{x}}^{-1})\frac{{\tilde{x}}^{1/2}-{\tilde{x}}^{-1/2}}{x^{1/2}-x^{-1/2}}\tau ^{-1/2} s_{IV}(\Phi ^{(\sigma _2)})(x;\tau ) \end{aligned}$$

where ${\tilde{x}}=e^{u/\tau }=e^{2\pi {\textrm{i}}N u/(u+2\pi {\textrm{i}})}$. This, together with Conjecture 6 implies

154 $$\begin{aligned} J^{\textbf{4}_1}_N(q)&= s_{\text {med}}(\Phi ^{(\sigma _0)})(x;\tau ) +\frac{{\tilde{x}}^{1/2}-{\tilde{x}}^{-1/2}}{x^{1/2}-x^{-1/2}} \tau ^{-1/2}s_{\text {med}}(\Phi ^{(\sigma _1)})(x;\tau )\nonumber \\&\quad -\frac{{\tilde{x}}-{\tilde{x}}^{-1}}{2}\frac{{\tilde{x}}^{1/2}-{\tilde{x}}^{-1/2}}{x^{1/2}-x^{-1/2}} \tau ^{-1/2}s_{\text {med}}(\Phi ^{(\sigma _2)})(x;\tau ) , \end{aligned}$$

which is Conjecture 2 for the $\textbf{4}_1$ knot.

We now make several consistency checks of the above conjecture. The first is that equation (154) is invariant under complex conjugation which moves $\tau$ from cone *I* to cone *IV*. The second is that the conjecture implies the Generalised Volume Conjecture. Indeed, in the limit

155 $$\begin{aligned} N\rightarrow \infty , \quad \tau \rightarrow 0, \quad \log (x) = 2\pi {\textrm{i}}N\tau \text { finite} \end{aligned}$$

the right hand side of (153a),(153b) are dominated by the first term. If we keep only the exponential, this is the generalised Volume Conjecture [Mur11, Guk05]. Recall from [Mur11], the generalised Volume Conjecture reads, for *u* in a small neighborhood of origin such that $u\not \in \pi {\textrm{i}}{\mathbb {Q}}$,

156 $$\begin{aligned} \lim _{N\rightarrow \infty }\frac{\log J_N^{K}(\exp ((u+2\pi {\textrm{i}})/N))}{N} = \frac{H(y,x)}{u+2\pi {\textrm{i}}}, \end{aligned}$$

where $x = \exp (u+2\pi {\textrm{i}})$ and $H(y,x) = \textrm{Li}_2(1/(xy)) - \textrm{Li}_2(y/x) + \log (x)\log (y)$, with *y* a solution to $y+y^{-1} = x+x^{-1}-1$. By the identification $u + 2\pi {\textrm{i}}= 2\pi {\textrm{i}}(N\tau ) \sim 2\pi {\textrm{i}}$, and since *A*(*x*) is identical with *H*(*y*, *x*) (up to $\pm 1$), one can check that (153a),(153b) imply (156).

## The $\textbf{5}_2$-knot

### A $3 \times 3$ matrix of *q*-series

The trace field of the $\textbf{5}_2$ knot is the cubic field of discriminant $-23$, with a distinguished complex embedding $\sigma _1$ (corresponding to the geometric representation of $\textbf{5}_2$), its complex conjugate $\sigma _2$ and a real embedding $\sigma _3$. The $\textbf{5}_2$ knot has three boundary parabolic representations whose associated asymptotic series $\varphi ^{(\sigma _j)}(h)$ for $j=1,2,3$ correspond to the three embeddings of the trace field. In [GGMn21] these asymptotic series were discussed, and a $3 \times 3$ matrix ${\textbf{J}}^\text {red}_m(q)$ of *q*-series was constructed to describe the resurgence properties of the asymptotic series. The matrix ${\textbf{J}}^\text {red}_m(q)$ is a fundamental solution to the linear *q*-difference equation [GGMn21, Eq. (23)]

157 $$\begin{aligned} f_m(q)-3f_{m+1}(q)+(3-q^{2+m})f_{m+2}(q) - f_{m+3}(q) = 0 \end{aligned}$$

and it is defined by^4^

159 $$\begin{aligned} {\textbf{J}}^\text {red}_{m}(q) = \begin{pmatrix} H_{m}^{(2)}(q) & H_{m+1}^{(2)}(q) & H_{m+2}^{(2)}(q) \\ H_{m}^{(1)}(q) & H_{m+1}^{(0)}(q) & H_{m+2}^{(1)}(q) \\ H_{m}^{(0)}(q) & H_{m+1}^{(0)}(q) & H_{m+2}^{(0)}(q) \end{pmatrix}, \qquad (|q| \ne 1) \end{aligned}$$

where for $$|q|<1$$

160 $$\begin{aligned} \begin{aligned} H^{(0)}_{m}(q)&=\sum _{n=0}^\infty \frac{q^{n(n+1)+nm}}{(q;q)_n^3},\\ H^{(1)}_{m}(q)&=\sum _{n=0}^\infty \frac{q^{n(n+1)+nm}}{(q;q)_n^3} \left( 1+2n+m-3E_1^{(n)}(q)\right) ,\\ H^{(2)}_{m}(q)&=\sum _{n=0}^\infty \frac{q^{n(n+1)+nm}}{(q;q)_n^3} \left( (1+2n+m-3E_1^{(n)}(q))^2-3E_2^{(n)}(q)-\frac{1}{6}E_2(q)\right) , \end{aligned} \end{aligned}$$

and

161 $$\begin{aligned} \begin{aligned} H^{(0)}_{-m}(q^{-1})&=\sum _{n=0}^\infty (-1)^n\frac{q^{\frac{1}{2}n(n+1)+nm}}{(q;q)_n^3},\\ H^{(1)}_{-m}(q^{-1})&=-\sum _{n=0}^\infty (-1)^n\frac{q^{\frac{1}{2}n(n+1)+nm}}{(q;q)_n^3} \left( \frac{1}{2}+n+m-3E_1^{(n)}(q)\right) ,\\ H^{(2)}_{-m}(q^{-1})&=\sum _{n=0}^\infty (-1)^n\frac{q^{\frac{1}{2}n(n+1)+nm}}{(q;q)_n^3} \left( \big (\frac{1}{2}+n+m-3E_1^{(n)}(q)\big )^2-3E_2^{(n)}(q) -\frac{1}{12}E_2(q)\right) . \end{aligned} \end{aligned}$$

### The Habiro polynomials and the descendant Kashaev invariants

The addition of the asymptotic series $\varphi ^{(\sigma _0)}(h)$ corresponding to the trivial flat connection requires a $4 \times 4$ extension of the matrix ${\textbf{J}}^\text {red}(q)$. This is consistent with the fact that the colored Jones polynomial of $\textbf{5}_2$ satisfies a third order inhomogenous linear *q*-difference equation, and hence a 4th order homogeneous linear *q*-difference equation. However, the descendant colored Jones polynomials of $\textbf{5}_2$ satisfy a 5th order inhomogeneous recursion [GK23, Eq. (14)], hence a 6th order homogeneous recursion. In view of this, we will give a $6 \times 6$ matrix ${\textbf{J}}(q)$ of *q*-series and we will use its $4 \times 4$ block to describe the resurgent structure of the asymptotic series $\varphi ^{(\sigma _0)}(h)$.

Let us recall the Habiro polynomials, the descendant colored Jones polynomials, the descendant Kashaev invariants and their recursions. The Habiro polynomials $H_n^{\textbf{5}_2}(q) \in \mathbb {Z}[q^{\pm 1}]$ are given by terminating *q*-hypergeometric sums

162 $$\begin{aligned} H_n^{\textbf{5}_2}(q) = (-1)^n q^{\frac{1}{2}n(n+3)} \sum _{k=0}^n q^{k(k+1)} \left( {\begin{array}{c}n\\ k\end{array}}\right) _q \end{aligned}$$

(see Habiro [Hab02a] and also Masbaum [Mas03]) where $$\left( {\begin{array}{c}a\\ b\end{array}}\right) _q = (q;q)_a/((q;q)_b (q;q)_{b-a})$$ is the *q*-binomial function. In [GS06], it was shown that $H_n=H_n^{\textbf{5}_2}(q)$ satisfies the linear *q*-difference equation

163 $$\begin{aligned} H_{n+2}^{\textbf{5}_2}(q) + q^{3 + n} (1 + q - q^{2 + n} + q^{4 + 2 n}) H_{n+1}^{\textbf{5}_2}(q) -q^{6 + 2 n} (-1 + q^{1 + n}) H_n^{\textbf{5}_2}(q) =0, \quad (n \ge 0)\nonumber \\ \end{aligned}$$

with initial conditions $H_n^{\textbf{5}_2}(q)=0$ for $n<0$ and $H_0^{\textbf{5}_2}(q)=1$. Actually, the above recursion is valid for all integers if we replace the right hand side of it by $\delta _{n+2,0}$. The recursion for the Habiro polynomials of $\textbf{5}_2$, together with Equation (91) and [Kou10], gives that $\textrm{DJ}^{(m)}=\textrm{DJ}^{\textbf{5}_2,(m)}(x,q)$, which is the descendant colored Jones polynomial defined by (91), satisfies the linear *q*-difference equation

164 $$\begin{aligned} &  (-1 + q^{1 + m}) (-1 + q^{2 + m}) x^2 \textrm{DJ}^{(m)} - q^{2 + m} (-1 + q^{2 + m}) x (1 + q + x + (1+ q) x^2) \textrm{DJ}^{(1 + m)} \nonumber \\ &  \quad + q^{3 + m} (q^{3 + m} +(- 1 + q^{2 + m} + q^{3 + m}) x +(- 2 - q + q^{2 + m} + 2 q^{3 + m} + q^{4 + m}) x^2 +(-1 + q^{2 + m} + q^{3 + m}) x^3 + q^{3 + m} x^4) \textrm{DJ}^{(2 + m)} \nonumber \\ &  \quad - q^{4 + m} (q^{3 + m} +(-1 + q^{3 + m} + q^{4 + m}) x +(-1 + q^{2 + m} + 2 q^{3 + m} + q^{4 + m} ) x^2 +(-1 + q^{3 + m} + q^{4 + m}) x^3 + q^{3 + m} x^4) \textrm{DJ}^{(3 + m)} \nonumber \\ &  \quad + q^{5 + m} x (q^{3 + m} + q^{4 + m} +(-1 + q^{4 + m}) x + (q^{3 + m} + q^{4 + m}) x^2) \textrm{DJ}^{(4 + m)} - q^{10 + 2 m} x^2 \textrm{DJ}^{(5 + m)}\nonumber \\ &  \quad = x (q^{2 + m} + q^{4 + m} + (1 - q^{1 + m} - 2 q^{3 + m} - q^{5 + m}) x + (q^{2 + m} + q^{4 + m}) x^2) H_0(q) + q^m x (1 - x q^{-1}) (1 - q x) H_1(q) . \end{aligned}$$

Using the values $H^{\textbf{5}_2}_0(q)=1$, $H^{\textbf{5}_2}_1(q)=-q^2-q^4$, it follows that the right hand side of the above recursion is $x^2$ for all *m*. Setting $x=1$, and renaming $\textrm{DJ}^{(m)}$ by $f_m(q)$, we arrive at the inhomogenous 5-th order *q*-difference equation satisfied by the descendant Kashaev invariant [GK23, Eq. (14)]

165 $$\begin{aligned} &  -q^{2m+10}f_{m+5}(q) + (3q^{2m+9} + 2q^{2m+8} - q^{m+5})f_{m+4}(q) + (-3q^{2m+8} - 6q^{2m+7} - q^{2m+6} + 3q^{m+4})f_{m+3}(q)\nonumber \\ &  \quad + (q^{2m+7} + 6q^{2m+6} + 3q^{2m+5} - q^{m+4} - 4q^{m+3})f_{m+2}(q) + (2q^{m+3} + 3q^{m+2})(1-q^{m+2})f_{m+1}(q)\nonumber \\ &  \quad + (1-q^{m+1})(1-q^{m+2})f_{m}(q)=1 \end{aligned}$$

valid for all integers *m*. Our aim is to define an explicit fundamental matrix solution to the corresponding sixth order homogenous linear *q*-difference equation (165). To do so, we define a 2-parameter family of deformations of the Habiro polynomials which satisfy a one-parameter deformation of the recursion of the Habiro polynomials. Motivated by the *q*-hypergeometric expression (162) for the Habiro polynomials, we define deformations of the Habiro polynomials, for $|q|\ne 1$, with appropriate normalisations

166 $$\begin{aligned} \begin{aligned} H_{n}(\varepsilon ,\delta ;q)&= \frac{(qe^{\varepsilon -\delta };q)_{\infty }(qe^{\delta };q)_{\infty }}{ (qe^{\varepsilon };q)_{\infty }(q;q)_{\infty }} \frac{(-1)^nq^{n(n+3)/2}e^{(n+1)\varepsilon }}{ e^{\frac{1}{12}\varepsilon ^2-\frac{1}{12}(\varepsilon \delta -\delta ^2)E_{2}(q)}} \sum _{k\in \mathbb {Z}}\frac{q^{k(k+1)}e^{(2k+1)\delta }(qe^{\varepsilon };q)_{n}}{ (qe^{\delta };q)_{k}(qe^{\varepsilon -\delta };q)_{n-k}}\\ H_{n}(\varepsilon ,\delta ;q^{-1})&= \frac{(qe^{\varepsilon +\delta };q)_{\infty }}{(qe^{\delta };q)_{\infty }^{2}} \frac{q^{-n(n+3)/2}e^{(n+3/2)\varepsilon }}{(-1)^{n}(e^{-\delta };q)_{\infty }(q;q)_{\infty }} \sum _{k\in \mathbb {Z}}(-1)^{k}q^{k(k+1)/2}e^{\delta k}\frac{(qe^{\delta };q)_{k-1}}{ (qe^{\varepsilon +\delta };q)_{k-n-1}} \end{aligned} \end{aligned}$$

where $n\in \mathbb {Z}$ and $|q|<1$. These deformations satisfy the recursion

167 $$\begin{aligned} H_{n+2}(\varepsilon ,\delta ;q) +e^{\varepsilon }q^{n+3}(1+q-e^{\varepsilon }q^{n+2}+e^{2\varepsilon }q^{2n+4}) H_{n+1}(\varepsilon ,\delta ;q) +e^{2\varepsilon }q^{2n+6}(1-e^{\varepsilon }q^{n+1})H_{n}(\varepsilon ,\delta ;q) =0\nonumber \\ \end{aligned}$$

obtained from (163) by replacing $q^n$ to $e^\varepsilon q^n$. Note that when $\varepsilon =0$, we cannot solve for $H_{-1}$ in terms of $H_{n}$ for $n\ge 0$ as discussed in [Par].^5^ It follows that the function

168 $$\begin{aligned} \begin{aligned} Q_{m}(\varepsilon ,\delta ;q)&= -e^{-\varepsilon }(1-e^{\varepsilon })^{2}\sum _{n=-\infty }^{-1}q^{mn}e^{m\varepsilon } H_{n}(\varepsilon ,\delta ;q)(qe^{\varepsilon };q)_{n}(q^{-1}e^{-\varepsilon };q^{-1})_{n}\\&= \sum _{n=0}^{\infty }\frac{q^{-mn-m}e^{m\varepsilon } H_{-1-n}(\varepsilon ,\delta ;q)}{(q^{-1}e^{\varepsilon };q^{-1})_{n}(qe^{-\varepsilon };q)_{n}} \end{aligned} \end{aligned}$$

is an inhomogenous solution of Equation (165). In particular, for $|q|<1$ we have

169 $$\begin{aligned} \begin{aligned} Q_{m}(\varepsilon ,\delta ;q)&= \frac{(qe^{\varepsilon -\delta };q)_{\infty }(qe^{\delta };q)_{\infty } (1-e^{\varepsilon -\delta })}{(qe^{\varepsilon };q)_{\infty }(q;q)_{\infty }e^{\frac{1}{12} \varepsilon ^2-\frac{1}{12}(\varepsilon \delta -\delta ^2)E_{2}(q)}(1-e^{\varepsilon })}\\&\quad \times \sum _{n=0}^{\infty }\sum _{k\in \mathbb {Z}}\frac{(-1)^nq^{(n+1)(n-2)/2-mn-m+k(k+1)}e^{(m-n) \varepsilon +(2k+1)\delta }(q^{-1}e^{\varepsilon -\delta };q^{-1})_{n+k}}{ (q^{-1}e^{\varepsilon };q^{-1})_{n}^{2}(qe^{\varepsilon };q)_{n}(qe^{\delta };q)_{k}}\\ Q_{m}(\varepsilon ,\delta ;q^{-1})&= \frac{(qe^{\varepsilon +\delta };q)_{\infty }}{(qe^{\delta };q)_{\infty }^{2}( e^{-\delta };q)_{\infty }(q;q)_{\infty }}\\&\quad \times \sum _{n=0}^{\infty }\sum _{k\in \mathbb {Z}}(-1)^{n+k} \frac{q^{-(n+1)(n-2)/2+mn+m+k(k+1)/2}e^{(m-n+1/2)\varepsilon +\delta k} (qe^{\delta };q)_{k-1}}{(qe^{\varepsilon +\delta };q)_{k+n} (qe^{\varepsilon };q)_{n}(q^{-1}e^{-\varepsilon };q^{-1})_{n}}. \end{aligned} \end{aligned}$$

We see that $Q_{m}(\varepsilon ,\delta ;q)$ is convergent for $|q|<1$ and all $m\in \mathbb {Z}$ and for $|q|>1$ and all $m\in \mathbb {Z}_{\ge 0}$. Moreover, $\varepsilon Q_{m}(\varepsilon ,\delta ;q)\in \mathbb {Z}((q))[[\varepsilon ,\delta ]]$ for $m\in \mathbb {Z}$ and $\delta ^{2}Q_{m}(\varepsilon ,\delta ;q^{-1})\in \mathbb {Z}((q))[[\varepsilon ,\delta ]]$ for $m\in \mathbb {Z}_{\ge 0}$. Substituting $Q$ for *f* in the LHS of Equation (165) gives a RHS of

170 $$\begin{aligned} \begin{aligned}&e^{(m-1)\varepsilon }(1-e^{\varepsilon })^{2}H_{0}(\varepsilon ,\delta ;q)\\&\quad -q^{m+4}e^{(m+1)\varepsilon }(1-q^{-1}e^{-\varepsilon })(1-e^{\varepsilon })^{3} (1-q^{-1}e^{\varepsilon })H_{-1}(\varepsilon ,\delta ;q). \end{aligned} \end{aligned}$$

In particular, for $|q|<1$ Equation (170) is

171 $$\begin{aligned} &  \frac{(qe^{\varepsilon -\delta };q)_{\infty }(qe^{\delta };q)_{\infty }}{ (qe^{\varepsilon };q)_{\infty }(q;q)_{\infty }e^{\frac{1}{12}\varepsilon ^2 -\frac{1}{12}(\varepsilon \delta -\delta ^2)E_{2}(q)}} \bigg (e^{m\varepsilon }(1-e^{\varepsilon })^{2} \sum _{k\in \mathbb {Z}}\frac{q^{k(k+1)}e^{(2k+1)\delta }}{(qe^{\delta };q)_{k} (qe^{\varepsilon -\delta };q)_{-k}}\nonumber \\ &  \quad +q^{m+3}e^{(m+1)\varepsilon }(1-q^{-1}e^{-\varepsilon }) (1-e^{\varepsilon })^{2}(1-q^{-1}e^{\varepsilon }) \sum _{k\in \mathbb {Z}}\frac{q^{k(k+1)}e^{(2k+1)\delta }}{(qe^{\delta };q)_{k} (qe^{\varepsilon -\delta };q)_{-1-k}}\bigg )\nonumber \\ &  \quad = \varepsilon ^{2}(1+O(\delta ))+O(\varepsilon ^{3}) \end{aligned}$$

and for $|q|>1$ Equation (170) is

172 $$\begin{aligned} \begin{aligned}&\frac{(q^{-1}e^{\varepsilon +\delta };q^{-1})_{\infty }}{ (q^{-1}e^{\delta };q^{-1})_{\infty }^{2} (e^{-\delta };q^{-1})_{\infty }(q^{-1};q^{-1})_{\infty }}\\&\quad \bigg (e^{(m+1/2)\varepsilon }(1-e^{\varepsilon })^{2} \sum _{k\in \mathbb {Z}}(-1)^{k}q^{-k(k+1)/2}e^{\delta k} \frac{(q^{-1}e^{\delta };q^{-1})_{k-1}}{ (q^{-1}e^{\varepsilon +\delta };q^{-1})_{k-1}}\;\\&\quad +q^{m+3}e^{(m+3/2)\varepsilon }(1-q^{-1}e^{-\varepsilon }) (1-e^{\varepsilon })^{3}(1-q^{-1}e^{\varepsilon })\\&\quad \sum _{k\in \mathbb {Z}}(-1)^{k}q^{-k(k+1)/2}e^{\delta k} \frac{(q^{-1}e^{\delta };q^{-1})_{k-1}}{ (q^{-1}e^{\varepsilon +\delta };q^{-1})_{k}}\bigg )\\&\quad = \varepsilon ^{2}+O(\varepsilon ^{3}). \end{aligned} \end{aligned}$$

### A $6 \times 6$ matrix of *q*-series

We now have all the ingredients to define the promised $6 \times 6$ matrix ${\textbf{J}}_{m}(q)$ of *q*-series for $|q| \ne 1$. Let us denote by $Q_{m}^{(a,b)}(q)$ the coefficient of $\varepsilon ^{a}\delta ^{b}$ in the expansion of $Q_{m}(q)$. We now define

173 $$\begin{aligned} \begin{aligned} {\textbf{J}}_{m}(q)&= \begin{pmatrix} 1 & Q^{(2,0)}_{m}(q) & Q^{(2,0)}_{m+1}(q) & Q^{(2,0)}_{m+2}(q) & Q^{(2,0)}_{m+3}(q) & Q^{(2,0)}_{m+4}(q)\\ 0 & Q^{(0,0)}_{m}(q) & Q^{(0,0)}_{m+1}(q) & Q^{(0,0)}_{m+2}(q) & Q^{(0,0)}_{m+3}(q) & Q^{(0,0)}_{m+4}(q)\\ 0 & Q^{(-1,2)}_{m}(q) & Q^{(-1,2)}_{m+1}(q) & Q^{(-1,2)}_{m+2}(q) & Q^{(-1,2)}_{m+3}(q) & Q^{(-1,2)}_{m+4}(q)\\ 0 & Q^{(0,2)}_{m}(q) & Q^{(0,2)}_{m+1}(q) & Q^{(0,2)}_{m+2}(q) & Q^{(0,2)}_{m+3}(q) & Q^{(0,2)}_{m+4}(q)\\ 0 & Q^{(1,0)}_{m}(q) & Q^{(1,0)}_{m+1}(q) & Q^{(1,0)}_{m+2}(q) & Q^{(1,0)}_{m+3}(q) & Q^{(1,0)}_{m+4}(q)\\ 0 & Q^{(1,2)}_{m}(q) & Q^{(1,2)}_{m+1}(q) & Q^{(1,2)}_{m+2}(q) & Q^{(1,2)}_{m+3}(q) & Q^{(1,2)}_{m+4}(q) \end{pmatrix} \qquad (|q|<1),\\ {\textbf{J}}_{m}(q)&= \begin{pmatrix} 1 & Q^{(2,0)}_{m}(q) & Q^{(2,0)}_{m+1}(q) & Q^{(2,0)}_{m+2}(q) & Q^{(2,0)}_{m+3}(q) & Q^{(2,0)}_{m+4}(q)\\ 0 & Q^{(1,-2)}_{m}(q) & Q^{(1,-2)}_{m+1}(q) & Q^{(1,-2)}_{m+2}(q) & Q^{(1,-2)}_{m+3}(q) & Q^{(1,-2)}_{m+4}(q)\\ 0 & Q^{(2,-2)}_{m}(q) & Q^{(2,-2)}_{m+1}(q) & Q^{(2,-2)}_{m+2}(q) & Q^{(2,-2)}_{m+3}(q) & Q^{(2,-2)}_{m+4}(q)\\ 0 & Q^{(1,0)}_{m}(q) & Q^{(1,0)}_{m+1}(q) & Q^{(1,0)}_{m+2}(q) & Q^{(1,0)}_{m+3}(q) & Q^{(1,0)}_{m+4}(q)\\ 0 & Q^{(0,-2)}_{m}(q) & Q^{(0,-2)}_{m+1}(q) & Q^{(0,-2)}_{m+2}(q) & Q^{(0,-2)}_{m+3}(q) & Q^{(0,-2)}_{m+4}(q)\\ 0 & Q^{(0,0)}_{m}(q) & Q^{(0,0)}_{m+1}(q) & Q^{(0,0)}_{m+2}(q) & Q^{(0,0)}_{m+3}(q) & Q^{(0,0)}_{m+4}(q) \end{pmatrix} \qquad (|q|>1). \end{aligned} \end{aligned}$$

The next theorem relates the above matrix to the linear *q*-difference equation (165).

#### Theorem 17

The matrix ${\textbf{J}}_{m}(q)$ is a fundamental solution to the linear *q*-difference equation

174 $$\begin{aligned} {\textbf{J}}_{m+1}(q)= &  {\textbf{J}}_{m}(q) A(q^m,q),\nonumber \\ A(q^m,q)= &  \begin{pmatrix} 1 & 0 & 0 & 0 & 0 & -q^{-2m-10}\\ 0 & 0 & 0 & 0 & 0 & (1-q^{m+1})(1-q^{m+2})q^{-2m-10}\\ 0 & 1 & 0 & 0 & 0 & (3 + 2q)(1-q^{m+1})q^{-m-8}\\ 0 & 0 & 1 & 0 & 0 & (q^{m+4} + 6q^{m+3} + 3q^{m+2} - q - 4)q^{-m-7}\\ 0 & 0 & 0 & 1 & 0 & (-3q^{m+4} - 6q^{m+3} - q^{m+2} + 3)q^{-m-6}\\ 0 & 0 & 0 & 0 & 1 & (3q^{m+4} + 2q^{m+3} - 1)q^{-m-5} \end{pmatrix} . \end{aligned}$$

and has

175 $$\begin{aligned} \begin{aligned} \det ({\textbf{J}}_{m}(q))&=q^{-20-7m}(q;q)_{\infty }^{9}(q^{-m-1};q)_{\infty }(q^{-m};q)_{\infty } \qquad (|q|<1),\\ \det ({\textbf{J}}_{m}(q))&= q^{-20-7m}(q^{-1};q^{-1})_{\infty }^{-9}(q^{-m-1};q^{-1})_{\infty }^{-1} (q^{-m-2};q^{-1})_{\infty }^{-1} \qquad (|q|>1). \end{aligned} \end{aligned}$$

#### Proof

Equation (174) follows from Equations (171), (172). The determinant is calculated using the determinant of $A(q^{m},q)$ and by considering the limiting behavior in *m*. $\square$

The construction of this matrix has used special *q*-hypergeometric formulae for the Habiro polynomials. However, this construction can be carried out more generally and will be developed in a later publication.

There is a similar, however more complicated, relation between ${\textbf{J}}_{-m}(q^{-1})$ with the first row replaced by Appell-Lerch type sums and ${\textbf{J}}_{m}(q)^{-1}$ as in Theorem 4. This indicates these matrices could come from the factorisation of a state-integral. We will not give this relation, since we do not need it for the purpose of resurgence. We will however, discuss an important block property of the matrix ${\textbf{J}}_{-2}(q)$, after a gauge transformation. Namely, we define:

176 $$\begin{aligned} {\textbf{J}}^{\textrm{norm}}(q) = {\textbf{J}}_{-2}(q) \begin{pmatrix} 1 & 0 & 0 & 0 & 0 & 0\\ 0 & 0 & 0 & 0 & 0 & q^{-1}-1\\ 0 & 0 & 0 & 0 & 1 & -3\\ 0 & -q & q & 3q^2 & 0 & 2q\\ 0 & 0 & q^2 & q^2-3q^3 & 0 & -q^2\\ 0 & 0 & 0 & q^4 & 0 & 0 \end{pmatrix} . \end{aligned}$$

The first few terms of the matrix ${\textbf{J}}^{\textrm{norm}}(q)+Q(q^3)$ are given by

177 $$\begin{aligned} \begin{pmatrix} 1 & -\frac{1}{12} + \frac{25}{12}q + 4q^2 & -\frac{5}{6} - \frac{19}{6}q - \frac{95}{12}q^2 & \frac{1}{12} - 2q - \frac{83}{12}q^2 & -\frac{5}{12} + \frac{11}{12}q - 3q^2 & \frac{5}{12} - \frac{1}{2}q + 2q^2\\ 0 & 1 - q & -2 + 2q - q^2 & -1 - q^2 & -1 + q & 1\\ 0 & -1 + 4q + q^2 & 1 - 7q + 2q^2 & -q + q^2 & 1 - 3q - q^2 & q^2\\ 0 & \frac{5}{12} - \frac{35}{12}q + \frac{13}{2}q^2 & \frac{2}{3} + \frac{4}{3}q - \frac{263}{12}q^2 & \frac{1}{12} - \frac{5}{2}q - \frac{137}{12}q^2 & -\frac{17}{12} + \frac{53}{12}q - \frac{13}{2}q^2 & -\frac{1}{12} + 4q + \frac{11}{2}q^2\\ 0 & 0 & 0 & 0 & 1 - 2q & -1 + q + 2q^2\\ 0 & 0 & 0 & 0 & \frac{11}{12} - \frac{11}{6}q + 10q^2 & \frac{1}{12} - \frac{61}{12}q - \frac{1}{6}q^2 \end{pmatrix} . \end{aligned}$$

We next discuss a block structure for the gauged-transform matrix (176).

#### Conjecture 18

When $|q|<1$, the matrix ${\textbf{J}}^{\textrm{norm}}(q)$ has a block form

178 $$\begin{aligned} \begin{pmatrix} 1\times 1 & 1\times 3 & 1\times 2\\ 0 & 3\times 3 & 3\times 2\\ 0 & 0 & 2\times 2 \end{pmatrix} . \end{aligned}$$

Our next task is to identify the $3\times 3$ and the $2\times 2$ blocks of the matrix ${\textbf{J}}^{\textrm{norm}}(q)$. The first observation is that the $3\times 3$ block is related to the $3\times 3$ matrix given in [GGMn21]. The second is that the $2\times 2$ block is related to modular forms. This is the content of the next conjecture.

#### Conjecture 19

The $3\times 3$ block for $|q|<1$ of ${\textbf{J}}^{\textrm{norm}}(q)$ of (176) has the form

179 $$\begin{aligned} (q;q)_{\infty } {\textbf{J}}_{-1}^\text {red}(q) \begin{pmatrix} 0& 0& 1\\ -1& 3& 0\\ 0& -1& 0 \end{pmatrix} \end{aligned}$$

(where ${\textbf{J}}_{m}^\text {red}(q)$ is the $3\times 3$ matrix of [GGMn21] reviewed in Sect. 4.1) and the $2\times 2$ block has the form

180 $$\begin{aligned} (q;q)_{\infty }^{2} \begin{pmatrix} H(q) & G(q)\\ * & * \end{pmatrix} \end{aligned}$$

where

181 $$\begin{aligned} H(q)=\sum _{k=0}^{\infty }\frac{q^{k^{2}+k}}{(q;q)_{k}} \quad \text {and}\quad G(q)=\sum _{k=0}^{\infty }\frac{q^{k^{2}}}{(q;q)_{k}} \end{aligned}$$

are the famous Rogers-Ramanujan functions.

The remaining two entries of the $2 \times 2$ block are higher weight vector-valued modular forms associated to the same $\textrm{SL}_{2}(\mathbb {Z})$-representation as the Rogers-Ramanujan functions, discussed for example in [Whe23]. Part of this conjecture is proved in Appendix A.

This block decomposition fits nicely with the “dream” in [Zaga]. Here we do see the interesting property that the $1\times 2$ and $3\times 2$ blocks contain some non-trivial gluing information. This implies that the diagrammatic “short exact sequence” will not always “split”. The block decomposition also implies that the resurgent structure of the asymptotic series associated to the *q*-series in the $4\times 4$ block in the top left does not depend on the other blocks. This block and in-particular the second column of ${\textbf{J}}^{\textrm{norm}}$ will be the focus of Sect. 4.4.

We now consider the analytic properties of the function

182 $$\begin{aligned} W(\tau )={\textbf{J}}^{\textrm{norm}}(e(\tau ))^{-1} \begin{pmatrix} \tau ^2 & 0 & 0 & 0 & 0 & 0\\ 0 & 1 & 0 & 0 & 0 & 0\\ 0 & 0 & \tau & 0 & 0 & 0\\ 0 & 0 & 0 & \tau ^2 & 0 & 0\\ 0 & 0 & 0 & 0 & \tau & 0\\ 0 & 0 & 0 & 0 & 0 & \tau ^3 \end{pmatrix} {\textbf{J}}^{\textrm{norm}}(\textbf{e}(-1/\tau )), \qquad (\tau \in \mathbb {C}\setminus \mathbb {R}) .\nonumber \\ \end{aligned}$$

If the work [GZ23] extended to the $6 \times 6$ matrix, it would imply that the function *W* extends to an analytic function on $\mathbb {C}'$. This would follow from an identification of *W* with a matrix of state-integrals, as was done in Sect. 2.7 for the $\textbf{4}_1$ knot. Although we do not know of such a matrix of state-integrals, we can numerically evaluate *W* when $\tau$ is near the positive real axis and test the extension hypothesis. Doing so for $\tau =1+\frac{{\textrm{i}}}{100}$ we have

183 $$\begin{aligned} \begin{aligned}&{\textbf{J}}^{\textrm{norm}}(\textbf{e}(-1/\tau ))\\&\quad = \begin{pmatrix} 1 & 1.9E^{9} + 3.8E^{8}{\textrm{i}}& -5.1E^{9} - 9.9E^{8}{\textrm{i}}& -4.5E^{9} - 8.8E^{8}{\textrm{i}}& -1.2E^{9} - 2.5E^{8}{\textrm{i}}& 2.9E^{9} + 5.7E^{8}{\textrm{i}}\\ 0 & 2.4E^{6} + 4.1E^{5}{\textrm{i}}& -6.1E^{6} - 1.0E^{6}{\textrm{i}}& -5.4E^{6} - 9.5E^{5}{\textrm{i}}& -1.5E^{6} - 2.7E^{5}{\textrm{i}}& 3.5E^{6} + 6.1E^{5}{\textrm{i}}\\ 0 & -1.3E^{-20} + 1.0E^{-20}{\textrm{i}}& 1.7E^{-20} - 2.6E^{-20}{\textrm{i}}& -6.2E^{-22} - 5.1E^{-21}{\textrm{i}}& 9.1E^{-21} - 2.5E^{-21}{\textrm{i}}& -4.0E^{-21} + 3.8E^{-21}{\textrm{i}}\\ 0 & 1.9E^{9} + 3.8E^{8}{\textrm{i}}& -5.1E^{9} - 9.9E^{8}{\textrm{i}}& -4.5E^{9} - 8.8E^{8}{\textrm{i}}& -1.2E^{9} - 2.5E^{8}{\textrm{i}}& 2.9E^{9} + 5.7E^{8}{\textrm{i}}\\ 0 & 0 & 0 & 0 & 3.1E^{-17} - 1.3E^{-17}{\textrm{i}}& -5.0E^{-17} + 2.1E^{-17}{\textrm{i}}\\ 0 & 0 & 0 & 0 & 2.6E^{-14} - 1.0E^{-14}{\textrm{i}}& -4.2E^{-14} + 1.7E^{-14}{\textrm{i}}\end{pmatrix} \end{aligned}\nonumber \\ \end{aligned}$$

where $$\textbf{e}(x)=e^{2 \pi {\textrm{i}}x}$$ whereas

184 $$\begin{aligned} W(\tau )= \begin{pmatrix} 0.99 - 0.019{\textrm{i}}& -0.10 - 0.028{\textrm{i}}& 0.24 - 0.25{\textrm{i}}& 0.060 - 0.43{\textrm{i}}& -0.064 + 0.059{\textrm{i}}& -0.18 - 0.094{\textrm{i}}\\ 0 & 0.59 - 1.0{\textrm{i}}& 1.0 + 1.3{\textrm{i}}& 0.19 - 0.13{\textrm{i}}& -0.60 - 0.20{\textrm{i}}& -0.48 - 0.22{\textrm{i}}\\ 0 & -0.17 - 0.17{\textrm{i}}& 1.2 - 0.30{\textrm{i}}& 0.024 - 0.31{\textrm{i}}& -0.14 - 0.0076{\textrm{i}}& -0.17 + 0.030{\textrm{i}}\\ 0 & 0.028 - 0.31{\textrm{i}}& 0.097 + 1.1{\textrm{i}}& 1.0 + 0.46{\textrm{i}}& -0.17 + 0.030{\textrm{i}}& -0.12 - 0.53{\textrm{i}}\\ 0 & 0 & 0 & 0 & 0.17 - 0.83{\textrm{i}}& -0.44 - 0.25{\textrm{i}}\\ 0 & 0 & 0 & 0 & -0.46 - 0.26{\textrm{i}}& 0.63 - 0.56{\textrm{i}}\end{pmatrix} . \end{aligned}$$

### Borel resummation and Stokes constants

The $\textbf{5}_2$ knot has four asymptotic series $\Phi ^{(\sigma _j)}(\tau )$ for $j=0,1,2,3$ corresponding to the trivial, the geometric, the conjugate, and the real flat connections respectively, denoted respectively by $\sigma _j$ for $j=0,1,2,3$. Similar to the $\textbf{4}_1$ knot, the asymptotic series $\Phi ^{(\sigma _j)}(\tau )$ for $j=1,2,3$ can be defined in terms of a perturbation theory of a state-integral [KLV16, AK14] using the standard formal Gaussian integration as explained in [DGLZ09, GGMn21], and they have been computed in [GGMn21] with more than 200 terms. Let $\xi _{j}$ ($j=1,2,3$) be the roots to the algebraic equation

185 $$\begin{aligned} (1-\xi )^3 = \xi ^2 \end{aligned}$$

with numerical values

186 $$\begin{aligned} \xi _1 = 0.78492\ldots +1.30714\ldots {\textrm{i}},\quad \xi _2 = 0.78492\ldots -1.30714\ldots {\textrm{i}},\quad \xi _3 = 0.43016\ldots .\nonumber \\ \end{aligned}$$

The asymptotic series $\Phi ^{(\sigma _j)}(\tau )$ for $j=1,2,3$ have the universal form^6^

188 $$\begin{aligned} \Phi ^{(\sigma _j)}(\tau ) = \frac{{\textrm{e}}^{\frac{3\pi {\textrm{i}}}{4}}}{\sqrt{\delta _j}} {\textrm{e}}^{\frac{V_j}{2\pi {\textrm{i}}\tau }} \varphi ^{(\sigma _j)}(\tau ),\quad j=1,2,3, \end{aligned}$$

where $$\delta _j = 5-3\xi _j+3\xi _j^2$$ and

189 $$\begin{aligned} \begin{aligned} V_1&= 3\textrm{Li}_2(\xi _1)+3/2\log (\xi _1)\log (1-\xi _1) -\pi {\textrm{i}}\log (\xi _1)-\frac{\pi ^2}{3}\\ V_2&= 3\textrm{Li}_2(\xi _2)+3/2\log (\xi _2)\log (1-\xi _2) +\pi {\textrm{i}}\log (\xi _2)-\frac{\pi ^2}{3},\\ V_3&= 3\textrm{Li}_2(\xi _3)+3/2\log (\xi _3)\log (1-\xi _3) -\frac{\pi ^2}{3}. \end{aligned} \end{aligned}$$

Their numerical values are given by

190 $$\begin{aligned} V_1= 3.0241\ldots + 2.8281\ldots {\textrm{i}},\quad V_2= 3.0241\ldots - 2.8281\ldots {\textrm{i}},\quad V_3= -1.1134\ldots .\nonumber \\ \end{aligned}$$

where the common absolute value of the imaginary parts of $V_1,V_2$ is the $\text {Vol}(S^3\backslash \textbf{5}_2)$. Finally the power series $\varphi ^{(\sigma _j)}(h/(2\pi {\textrm{i}}))$ with $h = 2\pi {\textrm{i}}\tau$ have coefficients in the number field $\mathbb {Q}(\xi _j)$ and their first few coefficients are given by

191 $$\begin{aligned} \varphi ^{(\sigma _j)}\left( \frac{h}{2\pi {\textrm{i}}}\right)= &  1+\frac{1452 \xi _j^2-1254 \xi _j +15949}{2^3\cdot 3\cdot 23^2} h \nonumber \\ &  + \frac{2124948 \xi _j ^2-2258148 \xi _j +11651375}{2^7\cdot 3^2\cdot 23^3} h^2 +\ldots \end{aligned}$$

The additional new series $\Phi ^{(\sigma _0)}(\tau ) \in {\mathbb {Q}}[[\tau ]]$ corresponds to the zero volume ($V(\sigma _0)=0$) trivial flat connection. As exlained in Sect. 2.3, it can be computed using the colored Jones polynomial or the Kashaev invariant. The first few terms are

192 $$\begin{aligned} \Phi ^{(\sigma _0)}(\tfrac{h}{2\pi {\textrm{i}}}) =\varphi ^{(\sigma _0)}(\tfrac{h}{2\pi {\textrm{i}}}) = 1 + 2h^2 + 6h^3+\frac{157}{6}h^4 + \ldots \end{aligned}$$

**Fig. 6:**
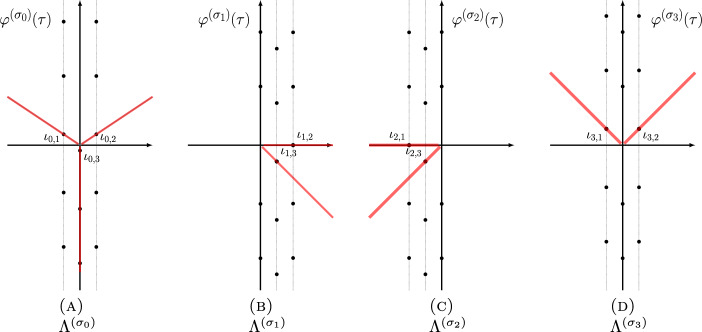
Singularities of Borel transforms of $\varphi ^{(\sigma _j)}(\tau )$ for $j=0,1,2,3$ of the knot $\textbf{5}_2$. Red lines are (some) Stokes rays

We are interested in the Stokes automorphism of the Borel resummation of the 4-vector $\Phi (\tau )$ of asymptotic series

193 $$\begin{aligned} \Phi (\tau ) = \begin{pmatrix} \Phi ^{(\sigma _0)}(\tau )\\ \Phi ^{(\sigma _1)}(\tau )\\ \Phi ^{(\sigma _2)}(\tau )\\ \Phi ^{(\sigma _3)}(\tau ) \end{pmatrix}. \end{aligned}$$

First of all, the Borel transform of each asymptotic series $\Phi ^{(\sigma _j)}(\tau )$ ($j=0,1,2,3$) has rich patterns of singularities. Similar to the case of $\textbf{4}_1$ knot discussed in Sect. 2.4, the Borel transforms of $\Phi ^{(\sigma _j)}(\tau )$, $j=1,2,3$ have singularities located at

194 $$\begin{aligned} \Lambda ^{(\sigma _j)} = \{\iota _{j,i} + 2\pi {\textrm{i}}k \,|\, i=1,2,3,i\ne j,\,k\in {\mathbb {Z}}\} \cup \{ 2\pi {\textrm{i}}k \,|\, k\in {\mathbb {Z}}_{\ne 0} \},\quad j=1,2,3\nonumber \\ \end{aligned}$$

as shown in the right three panels of Fig. 6, while the Borel transform of $\Phi ^{(\sigma _0)}(\tau )$ have singularities located at (some of these singular points are actually missing as we will comment in the end of the section.)

195 $$\begin{aligned} \Lambda ^{(\sigma _0)} = \{\iota _{0,i}+2\pi {\textrm{i}}k \,|\, i=1,2,3,\,k\in {\mathbb {Z}}\}, \end{aligned}$$

as shown in the left most panel of Fig. 6, where

196 $$\begin{aligned} \iota _{j,i} = \frac{V_j-V_i}{2\pi {\textrm{i}}},\quad i,j=0,1,2,3. \end{aligned}$$

To any singularity located at $\iota _{i,j}^{(k)}:= \iota _{i,j}+2\pi {\textrm{i}}k$ in the union

197 $$\begin{aligned} \Lambda = \cup _{j=0,1,2,3} \Lambda ^{(\sigma _j)}, \end{aligned}$$

we can associate a local Stokes matrix

198 $$\begin{aligned} \mathfrak {S}_{\iota _{i,j}^{(k)}}({\tilde{q}}) = I + \mathcal {S}_{i,j}^{(k)}{\tilde{q}}^k E_{i,j},\quad \mathcal {S}_{i,j}^{(k)}\in {\mathbb {Z}}, \end{aligned}$$

where $E_{i,j}$ is the $4\times 4$ elementary matrix with (*i*, *j*)-entry 1 ($i,j=0,1,2,3$) and all other entries zero, and $\mathfrak {S}_{i,j}^{(k)}$ is the Stokes constant. Then the Borel resummation along the rays $\rho _{\theta _\pm }$ raised slight above and below the angle $\theta$ are related by the Stokes automorphism

199 $$\begin{aligned} \Delta (\tau )s_{\theta _+}(\Phi )(\tau ) = \mathfrak {S}_{\theta }({\tilde{q}})\Delta (\tau ) s_{\theta _-}(\tau ), \end{aligned}$$

where

200 $$\begin{aligned} \mathfrak {S}_\theta ({\tilde{q}}) = \prod _{\arg \iota = \theta }\mathfrak {S}_{\iota }({\tilde{q}}),\quad \Delta (\tau ) = \textrm{diag}(\tau ^{3/2},1,1,1), \end{aligned}$$

and the locality condition is assumed.

**Fig. 7:**
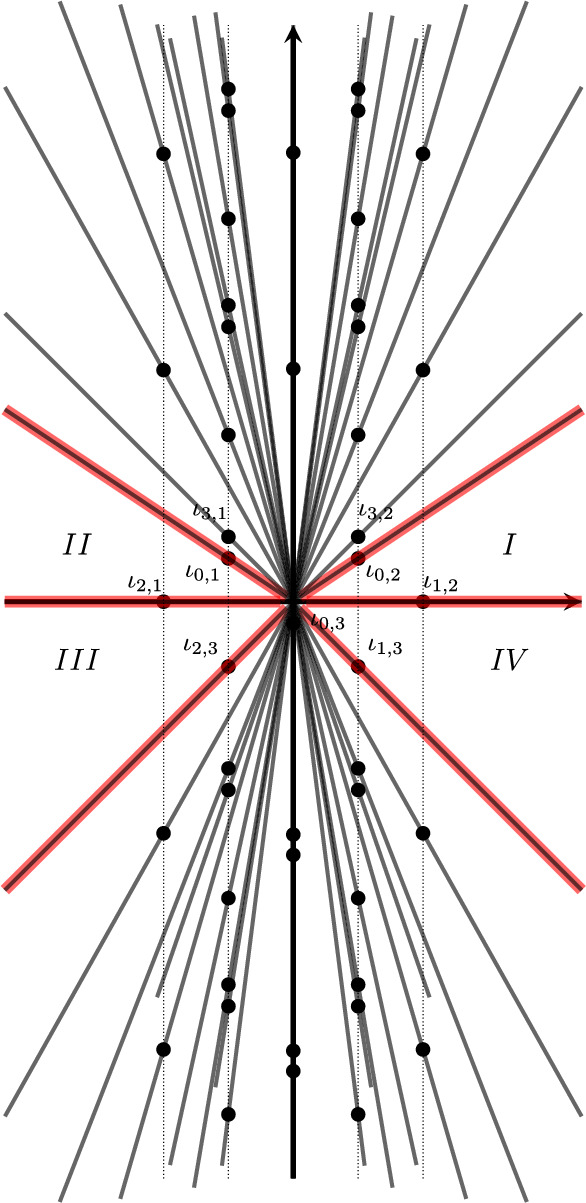
Stokes rays and cones in the $\tau$-plane for the 4-vector $\Phi (\tau )$ of asymptotic series of the knot $\textbf{5}_2$. Red lines are (some) Stokes rays

More generally, for two rays $\rho _{\theta ^+}$ and $\rho _{\theta ^-}$ whose arguments satisfy $0<\theta ^+-\theta ^-\le \pi$, we can define the global Stokes matrix $\mathfrak {S}_{\theta ^-\rightarrow \theta ^+}$ as in (44), and it also satisfies the factorisation property (45). Since the factorisation is unique [GGMn21, GGMn23], we only need to compute finitely many global Stokes matrices in order to extract all the local Stokes matrices associated to the infinitely many singularities in $\Lambda$ and thus the corresponding Stokes constants. In particular, we can choose four cones *I*, *II*, *III*, *IV* slightly above the positive and the negative real axes as shown in Fig. 7, and compute the four global Stokes matrices

201 $$\begin{aligned} \mathfrak {S}_{I\rightarrow II},\;\mathfrak {S}_{II\rightarrow III},\; \mathfrak {S}_{III\rightarrow IV},\;\mathfrak {S}_{IV\rightarrow I}, \end{aligned}$$

where a cone *R* in the subscript means any ray inside the cone.

On the other hand, each of the global Stokes matrices in (201) has the block upper triangular form

202 $$\begin{aligned} \begin{pmatrix} 1& *& *& *\\ 0& *& *& *\\ 0& *& *& *\\ 0& *& *& * \end{pmatrix}. \end{aligned}$$

The $3\times 3$ sub-matrices $\mathfrak {S}^{\text {red}}_{R\rightarrow R'}$ in the right bottom have been worked out in [GGMn21]. For later convenience, we write down two of the four reduced global Stokes matrices,

203a $$\begin{aligned} \mathfrak {S}^{\text {red}}_{I\rightarrow II}({\tilde{q}})&=\frac{1}{2} \begin{pmatrix} 0& 1& 0\\ 0& 1& 1\\ -1& 0& 0 \end{pmatrix}{\textbf{J}}^\text {red}_{-1}({\tilde{q}}^{-1})^T \begin{pmatrix} 0& 0& 1\\ 0& -2& 0\\ 1& 0& 0 \end{pmatrix}{\textbf{J}}^\text {red}_{-1}({\tilde{q}}) \begin{pmatrix} 0& 0& -1\\ 1& -3& 0\\ 0& 1& 0 \end{pmatrix}, \quad |{\tilde{q}}|<1, \end{aligned}$$

203b $$\begin{aligned} \mathfrak {S}^{\text {red}}_{III\rightarrow IV}({\tilde{q}})&=\frac{1}{2} \begin{pmatrix} 1& -3& 0\\ 0& 1& 0\\ 0& 0& -1 \end{pmatrix}{\textbf{J}}^\text {red}_{-1}({\tilde{q}}^{-1})^T \begin{pmatrix} 0& 0& 1\\ 0& -2& 0\\ 1& 0& 0 \end{pmatrix}{\textbf{J}}^\text {red}_{-1}({\tilde{q}}) \begin{pmatrix} 1& 0& 0\\ 1& 1& 0\\ 0& 0& -1 \end{pmatrix}, \quad |{\tilde{q}}| > 1. \end{aligned}$$

In addition, as seen from Fig. 6, there are no singularities along the positive and negative real axes in $\Lambda ^{(\sigma _0)}$ relevant for $\Phi ^{(\sigma _0)}(\tau )$; all the singular points in $\Lambda ^{(\sigma _0)}$ are either in the upper half plane beyond the cones *I*, *II* or in the lower half plane beneath the cones *III*, *IV*. Consequently we only need to compute the first row of two Stokes matrices $\mathfrak {S}_{I\rightarrow II}$ and $\mathfrak {S}_{III\rightarrow IV}$. For this purpose, we find the following.

#### Conjecture 20

For every cone $R \subset \mathbb {C}\setminus \Lambda$ and every $\tau \in R$, we have

204 $$\begin{aligned} Q_0^{(2,0)}(q) = s_R(\Phi ^{(\sigma _0)})(\tau ) + \tau ^{-3/2}\sum _{j=1}^3 M_{R,j}({\tilde{q}})s_R(\Phi ^{(\sigma _j)})(\tau ), \end{aligned}$$

where $M_{R,j}({\tilde{q}})$ ($j=1,2,3$) are ${\tilde{q}}$ (resp., ${\tilde{q}}^{-1}$)-series if $\textrm{Im}\tau >0$ (resp., $\textrm{Im}\tau <0$) with integer coefficients that depend on *R*.

A more elegant way to present $M_{R,j}({\tilde{q}})$ is by the row vector $M_R({\tilde{q}}):= (M_{R,1},M_{R,2},M_{R,3})({\tilde{q}})$, and it can be expressed in terms of a $3\times 3$ matrix $M^{(\sigma _0)}_R({\tilde{q}})$

205 $$\begin{aligned} M_R({\tilde{q}}) = \left( {\tilde{q}}Q^{(2,0)}_0({\tilde{q}}),{\tilde{q}}^2Q_1^{(2,0)}({\tilde{q}}), {\tilde{q}}^3Q_2^{(2,0)}({\tilde{q}})\right) M^{(\sigma _0)}_R({\tilde{q}}). \end{aligned}$$

#### Conjecture 21

Equation (204) holds in the cones $R=I,II,III,IV$ where the ${\tilde{q}}$,${\tilde{q}}^{-1}$-series $M_{R,j}({\tilde{q}})$ are given in terms of $M_R^{(0)}({\tilde{q}})$ through (236) which are as follows

206a $$\begin{aligned} M^{(\sigma _0)}_{I}({\tilde{q}})&= \begin{pmatrix} 1& -1& -3{\tilde{q}}\\ 0& -1& -1+3{\tilde{q}}\\ 0& 0& -{\tilde{q}}\end{pmatrix},\end{aligned}$$

206b $$\begin{aligned} M^{(\sigma _0)}_{II}({\tilde{q}})&= \begin{pmatrix} -1& 1& -3{\tilde{q}}\\ -1& 0& -1+3{\tilde{q}}\\ 0& 0& -{\tilde{q}}\end{pmatrix},\end{aligned}$$

206c $$\begin{aligned} M^{(\sigma _0)}_{III}({\tilde{q}})&= \begin{pmatrix} 3& 1& -3{\tilde{q}}\\ -1& 0& -1+3{\tilde{q}}\\ 0& 0& -{\tilde{q}}\end{pmatrix},\end{aligned}$$

206d $$\begin{aligned} M^{(\sigma _0)}_{IV}({\tilde{q}})&= \begin{pmatrix} 1& 3& -3{\tilde{q}}\\ 0& -1& -1+3{\tilde{q}}\\ 0& 0& -{\tilde{q}}\end{pmatrix}. \end{aligned}$$

Equations (204), together with the reduced Stokes matrices $\mathfrak {S}^{\text {red}}_{R\rightarrow R'}({\tilde{q}})$ for $\Phi ^{(\sigma _j)}(\tau )$ ($j=1,2,3$), allow us to calculate entries in the first row of $\mathfrak {S}_{I\rightarrow II}({\tilde{q}})$ and $\mathfrak {S}_{III\rightarrow IV}({\tilde{q}})$ by

207 $$\begin{aligned} \mathfrak {S}_{R\rightarrow R'}({\tilde{q}})_{0,j} = M_{R,j}({\tilde{q}}) - \sum _{k=1}^3M_{R',k}({\tilde{q}}) \mathfrak {S}^{\text {red}}_{R\rightarrow R'}({\tilde{q}})_{k,j},\quad j=1,2,3. \end{aligned}$$

In the following we list the first few terms of these ${\tilde{q}}$ and ${\tilde{q}}^{-1}$-series. In the upper half plane

208a $$\begin{aligned} \mathfrak {S}_{I\rightarrow II}({\tilde{q}})_{0,1}&= -1+13{\tilde{q}}-12{\tilde{q}}^2-82{\tilde{q}}^3-29{\tilde{q}}^4+85{\tilde{q}}^5+O({\tilde{q}}^6) ,\end{aligned}$$

208b $$\begin{aligned} \mathfrak {S}_{I\rightarrow II}({\tilde{q}})_{0,2}&= 1-16{\tilde{q}}+42{\tilde{q}}^2+135{\tilde{q}}^3-54{\tilde{q}}^4-346{\tilde{q}}^5+O({\tilde{q}}^6) ,\end{aligned}$$

208c $$\begin{aligned} \mathfrak {S}_{I\rightarrow II}({\tilde{q}})_{0,3}&= -{\tilde{q}}+10{\tilde{q}}^2+18{\tilde{q}}^3-31{\tilde{q}}^4-92{\tilde{q}}^5+O({\tilde{q}}^6). \end{aligned}$$

In the lower half plane

209a $$\begin{aligned} \mathfrak {S}_{III\rightarrow IV}({\tilde{q}})_{0,1}&= 4{\tilde{q}}^{-1}-4{\tilde{q}}^{-2}-51{\tilde{q}}^{-3}-62{\tilde{q}}^{-4}-27{\tilde{q}}^{-5}+O({\tilde{q}}^{-6}) ,\end{aligned}$$

209b $$\begin{aligned} \mathfrak {S}_{III\rightarrow IV}({\tilde{q}})_{0,2}&= 3{\tilde{q}}^{-1}+2{\tilde{q}}^{-2}-26{\tilde{q}}^{-3}-47{\tilde{q}}^{-4}-64{\tilde{q}}^{-5}+O({\tilde{q}}^{-6}) ,\end{aligned}$$

209c $$\begin{aligned} \mathfrak {S}_{III\rightarrow IV}({\tilde{q}})_{0,3}&= -1+{\tilde{q}}^{-2}+18{\tilde{q}}^{-3}+39{\tilde{q}}^{-4}+73{\tilde{q}}^{-5}+O({\tilde{q}}^{-6}). \end{aligned}$$

Finally, we can factorise the global Stokes matrices $\mathfrak {S}_{I\rightarrow II}({\tilde{q}}), \mathfrak {S}_{III\rightarrow IV}({\tilde{q}})$ to obtain local Stokes matrices associated to individual singular points in $\Lambda$ and extract the associated Stokes constants. The Stokes constants for $\Phi ^{(\sigma _j)}(\tau )$ ($j=1,2,3$) are already given in [GGMn21, GGMn23]. We collect the Stokes contants for $\Phi ^{(\sigma _0)}(\tau )$ in the generating series

210 $$\begin{aligned} \textsf{S}^+_{0,j}({\tilde{q}}) = \sum _{k\ge 0} \mathcal {S}^{(k)}_{0,j}{\tilde{q}}^k,\quad \textsf{S}^-_{0,j}({\tilde{q}}) = \sum _{k\le 0} \mathcal {S}^{(k)}_{0,j}{\tilde{q}}^k,\quad j=1,2,3. \end{aligned}$$

And we find that in the upper half plane

211a $$\begin{aligned} \textsf{S}^+_{0,1}({\tilde{q}})&= -1+{\tilde{q}}+3{\tilde{q}}^2+25{\tilde{q}}^3+278{\tilde{q}}^4+3067{\tilde{q}}^5+O({\tilde{q}}^6),\end{aligned}$$

211b $$\begin{aligned} \textsf{S}^+_{0,2}({\tilde{q}})&= 1-{\tilde{q}}-3{\tilde{q}}^2-25{\tilde{q}}^3-278{\tilde{q}}^4-3067{\tilde{q}}^5+O({\tilde{q}}^6),\end{aligned}$$

211c $$\begin{aligned} \textsf{S}^+_{0,3}({\tilde{q}})&= 0, \end{aligned}$$

while in the lower half plane

212a $$\begin{aligned} \textsf{S}^-_{0,1}({\tilde{q}})&= 3{\tilde{q}}^{-1}-34{\tilde{q}}^{-2}+391{\tilde{q}}^{-3}-4622{\tilde{q}}^{-4} +54388{\tilde{q}}^{-5}+O({\tilde{q}}^{-6}),\end{aligned}$$

212b $$\begin{aligned} \textsf{S}^-_{0,2}({\tilde{q}})&= 3{\tilde{q}}^{-1}-34{\tilde{q}}^{-2}+391{\tilde{q}}^{-3}-4622{\tilde{q}}^{-4} +54388{\tilde{q}}^{-5}+O({\tilde{q}}^{-6}),\end{aligned}$$

212c $$\begin{aligned} \textsf{S}^-_{0,3}({\tilde{q}})&= -1+9{\tilde{q}}^{-1}-56{\tilde{q}}^{-2}+705{\tilde{q}}^{-3}-8378{\tilde{q}}^{-4} +98379{\tilde{q}}^{-5}+O({\tilde{q}}^{-6}). \end{aligned}$$

We comment that the results of $\textsf{S}^+_{0,3}({\tilde{q}})$ and $\textsf{S}^-_{0,3}({\tilde{q}})$ indicate that there are actually no singular points of the type $\iota _{0,3}^{(k)}$ in the upper half plane, but they exist in the lower half plane. Also note that the constant terms in $\textsf{S}^+_{0,1}({\tilde{q}}),\textsf{S}^+_{0,2}({\tilde{q}})$ and $\textsf{S}^-_{0,3}({\tilde{q}})$ are Stokes constants associated to the singular points $\iota _{0,j}$ ($j=1,2,3$). The Stokes constants associated to $\iota _{i,j}$ $(i,j=1,2,3, i\ne j)$ have already been computed in [GGMn21, GGMn23]. We can assemble all these Stokes constants in a matrix

213 $$\begin{aligned} \begin{pmatrix} 0& -1& 1& -1\\ 0& 0& 4& 3\\ 0& -4& 0& -3\\ 0& -3& 3& 0 \end{pmatrix} \end{aligned}$$

which matches (after some changes of signs) the one appearing in [GZ24, Eq. (40)].

### (*x*, *q*)-series

In this section we extend the results of Sect. 4.1 by including the Jacobi variable *x*. Recall that the matrix ${\textbf{J}}^{\text {red}}_{m}(x,q)$^7^is a fundamental solution to the linear *q*-difference equation

216 $$\begin{aligned} f_m(x,q)-(1+x+x^{-1})f_{m+1}(x,q)+(1+x+x^{-1}-q^{2+m})f_{m+2}(x,q) - f_{m+3}(x,q) = 0\nonumber \\ \end{aligned}$$

and it is defined by

214 $$\begin{aligned} {\textbf{J}}^\text {red}_m(x,q) = \begin{pmatrix} A_m(x,q)& A_{m+1}(x,q)& A_{m+2}(x,q)\\ B_m(x,q)& B_{m+1}(x,q)& B_{m+2}(x,q)\\ C_m(x,q)& C_{m+1}(x,q)& C_{m+2}(x,q)\\ \end{pmatrix},\quad |q|\ne 1, \end{aligned}$$

where the holomorphic blocks are given by

217a $$\begin{aligned} A_m(x,q)&= H(x,x^{-1},q^m;q), \end{aligned}$$

217b $$\begin{aligned} B_m(x,q)&= \theta (-q^{1/2}x;q)^{-2}x^m H(x,x^2,q^mx^2;q), \end{aligned}$$

217c $$\begin{aligned} C_m(x,q)&= \theta (-q^{-1/2}x;q)^{-2}x^{-m} H(x^{-1},x^{-2},q^mx^{-2};q), \end{aligned}$$

where $H(x,y,z;q^\varepsilon ):=H^\varepsilon (x,y,z;q)$ for $|q|<1$ and $\varepsilon =\pm$ and

218a $$\begin{aligned} H^+(x,y,z;q)&=(qx;q)_\infty (qy;q)_\infty \sum _{n=0}^\infty \frac{q^{n(n+1)}z^n}{(q;q)_n(qx;q)_n(qy;q)_n},\end{aligned}$$

218b $$\begin{aligned} H^-(x,y,z;q)&= \frac{1}{(x;q)_\infty (y;q)_\infty }\sum _{n=0}^\infty (-1)^n\frac{q^{\frac{1}{2}n(n+1)}x^{-n}y^{-n}z^n}{(q;q)_n(qx^{-1};q)_n(qy^{-1};q)_n},\end{aligned}$$

218c $$\begin{aligned} \theta (x;q)&=(-q^{\frac{1}{2}}x;q)_{\infty }(-q^{\frac{1}{2}}x^{-1};q)_{\infty }. \end{aligned}$$

To these series we wish to add an additional series which satisfies the inhomogenous *q*-difference equations of the descendant coloured Jones polynomial (164). This can be easily constructed using the deformations of the Habiro polynomials (166). We find a solution

219 $$\begin{aligned} D_m(x,q) = -\sum _{n=-\infty }^{-1}q^{mn} H_{n}(q)x^{-n}(qx;q)_{n}(q^{-1}x;q^{-1})_{n}. \end{aligned}$$

(compare with Equation (91)) where $|q|<1$ and $m\in \mathbb {Z}$ or $|q|>1$ and $m\in \mathbb {Z}_{\ge 0}$, and $H_{n}(q)$ is the coefficient of $\varepsilon ^0\delta ^0$ in the expansion of $H_{n}(\epsilon ,\delta ;q)$. In particular, for $|q|<1$ we have

220 $$\begin{aligned} D_m(x,q) = -\sum _{n,k=0}^\infty (-1)^kq^{n(n+1)+k(k+1)/2-nk-(m+1)(n+1)} \frac{(q;q)_{n+k}}{(q;q)_k(q;q)_n(x^{-1};q)_{n+1}(x;q)_{n+1}}\nonumber \\ \end{aligned}$$

and we see the (*x*, *q*)-series $D_0(x,q)$ coincides with $f_{\textbf{5}_2}(x,q)$ in [Par20, Par].

This series can be included as the first row of a $6\times 6$ matrix of (*x*, *q*)-series. The latter might be related to the factorisation of the state integral proposed in Sect. 4.8.

However, we find that the matrices above and below the reals have different quantum modular co-cycles related by inversion. This implies that to do a full discussion on resurgence one needs to understand the monodromy of this *q*-holonomic system. Both these issue will be explored in later publications. For now, we give a description of the Stokes matrices restricted to $\tau$ in the upper half plane.

### *x*-version of Borel resummation and Stokes constants

In this section we discuss the *x*-deformation version of Sect. 4.4. The asymptotic series $\Phi ^{(\sigma _j)}(\tau )$ for $j=0,1,2,3$ are extended to series $\Phi ^{(\sigma _j)}(x;\tau )$ with coefficients in ${\mathbb {Z}}(x^{\pm 1})$. The series $\Phi ^{(\sigma _j)}(x;\tau )$ for $j=1,2,3$ are defined in terms of perturbation theory of a deformed state-integral [AK14] and they have been computed with about 200 terms for many values of *x* in [GGMn23]. Let $\xi _j$ ($j=1,2,3$) be three roots to the equation

221 $$\begin{aligned} (1-\xi )(1-x\xi )(1-x^{-1}\xi ) = \xi ^2, \end{aligned}$$

ordered such that they reduce to (186) in the limit $x\rightarrow 1$. The series $\Phi ^{(\sigma _j)}(\tau )$ ($j=1,2,3$) can be uniformly written as^8^

223 $$\begin{aligned} \Phi ^{(\sigma _j)}(x;\tau ) = \frac{{\textrm{e}}^{\frac{3\pi {\textrm{i}}}{4}}}{\sqrt{\delta _j(x)}} {\textrm{e}}^{\frac{V_j(x)}{2\pi {\textrm{i}}\tau }} \varphi ^{(\sigma _j)}(x;\tau ) \end{aligned}$$

where $$\delta _j(x) = \xi _j-s \xi _j^{-1}+2 \xi _j^{-2}$$ and

224 $$\begin{aligned} \begin{aligned} V_1(x)&=-\textrm{Li}_2(\xi _1^{-1})-\textrm{Li}_2(x\xi _1^{-1})-\textrm{Li}_2(x^{-1}\xi _1^{-1}) +\frac{1}{2}\log ^2 x -\frac{1}{2}\log ^2 \xi _1 +\pi {\textrm{i}}\log \xi _1+\frac{2\pi ^2}{3},\\ V_2(x)&=-\textrm{Li}_2(\xi _2^{-1})-\textrm{Li}_2(x\xi _2^{-1})-\textrm{Li}_2(x^{-1}\xi _2^{-1}) +\frac{1}{2}\log ^2 x -\frac{1}{2}\log ^2 \xi _2 -\pi {\textrm{i}}\log \xi _2+\frac{2\pi ^2}{3},\\ V_3(x)&=-\textrm{Li}_2(\xi _3^{-1})-\textrm{Li}_2(x\xi _3^{-1})-\textrm{Li}_2(x^{-1}\xi _3^{-1}) +\frac{1}{2}\log ^2 x -\frac{1}{2}\log ^2 \xi _3 +3\pi {\textrm{i}}\log \xi _3+\frac{2\pi ^2}{3}. \end{aligned}\nonumber \\ \end{aligned}$$

The power series $\varphi ^{(\sigma _j)}(x;\tau )$ are

225 $$\begin{aligned} \varphi ^{(\sigma _j)}(x;\tfrac{h}{2\pi {\textrm{i}}})&= 1 + \frac{h}{12\delta _j(x)} \big ((-397 - 94 s - 114 s^2 + 390 s^3 - 278 s^4 + 81 s^5 - 10 s^6)\nonumber \\&\quad +(-381 + 623 s - 124 s^2 - 328 s^3 + 268 s^4 - 81 s^5 + 10 s^6) \xi _j\nonumber \\&\quad +(-270 + 137 s + 182 s^2 - 207 s^3 + 71 s^4 - 10 s^5) \xi _j^2 \big )+\ldots \end{aligned}$$

with $$h=2\pi {\textrm{i}}\tau $$ and

226 $$\begin{aligned} s = s(x) = x^{-1}+1+x. \end{aligned}$$

The additional series $\Phi ^{(\sigma _0)}(x;\tau )$, as in Sect. 3.1, can be computed either from the colored Jones polynomial or by using Habiro’s expansion of the colored Jones polynomials. We find

227 $$\begin{aligned} \Phi ^{(\sigma _0)}(x;\tau ) = \varphi ^{(\sigma _0)}(x;\tau ), \end{aligned}$$

where the power series $\varphi ^{(\sigma _0)}(x;\tau )$ reads

228 $$\begin{aligned} \phi ^{(\sigma _0)}(x;\tfrac{h}{2\pi {\textrm{i}}}) = \frac{1}{2x+2x^{-1}-3} -\frac{(x^{1/2}-x^{-1/2})^2(5x+5x^{-1}-4)}{(2x+2x^{-1}-3)^3}h+\ldots , \end{aligned}$$

**Fig. 8:**
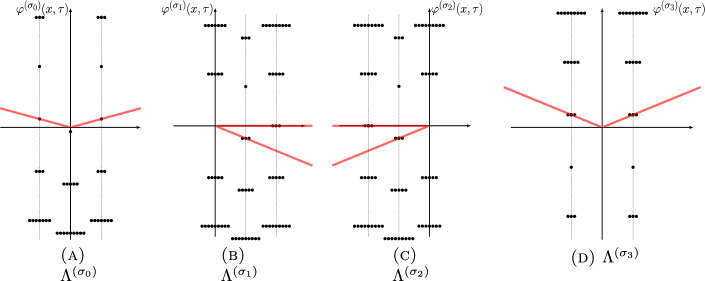
Singularities of Borel transforms of $\varphi ^{(\sigma _j)}(x,\tau )$ for $j=0,1,2,3$ of the knot $\textbf{5}_2$. Here we take small and real *x*. Red lines are (some) Stokes rays

We are interested in the Stokes automorphisms in the upper half plane of the Borel resummation of the 4-vector $\Phi (x;\tau )$ of asymptotic series

229 $$\begin{aligned} \Phi (x;\tau ) = \begin{pmatrix} \Phi ^{(\sigma _0)}(x;\tau )\\ \Phi ^{(\sigma _1)}(x;\tau )\\ \Phi ^{(\sigma _2)}(x;\tau )\\ \Phi ^{(\sigma _3)}(x;\tau ) \end{pmatrix}, \end{aligned}$$

when *x* is close to 1. The singular points of the Borel transform of $\Phi (x;\tau )$, collectively denoted as $\Lambda (x)$, are smooth functions of *x* and they are equal to $\Lambda$ in (197) in the limit $x\rightarrow 1$. When *x* is slightly away from 1, each singular point $\iota _{i,j}^{(k)}$ in $\Lambda$ splits to a finite set of points located at $\iota _{i,j}^{(k,\ell )}:=\iota _{i,j}^{(k)}+\ell \log (x)$, $\ell \in {\mathbb {Z}}$. We illustrate this schematically in Fig. 8. The complex plane of $\tau$ is divided by rays passing through these singular points into infinitely many cones. We will then pick the cones *I* and *II* located slightly above the positive and negative real axes, and compute the global Stokes matrix from cone *I* to cone *II* defined by

230 $$\begin{aligned} \Delta (x,\tau ) s_{II}(x,\tau ) = \mathfrak {S}_{I\rightarrow II}({\tilde{x}},{\tilde{q}}) \Delta (x,\tau ) s_{I}(x,\tau ), \end{aligned}$$

where

231 $$\begin{aligned} \Delta (x,\tau ) = \textrm{diag}\left( \tau ^{1/2}\frac{x^{1/2}-x^{-1/2}}{{\tilde{x}}^{1/2}-{\tilde{x}}^{-1/2}},1,1,1\right) . \end{aligned}$$

The global Stokes matrix $\mathfrak {S}_{I\rightarrow II}({\tilde{x}},{\tilde{q}})$ factorises uniquely into a product of local Stokes automorphisms associated to each of the singular points in the upper half plane, from which the individual Stokes constants can be read off.

The global Stokes matrix $\mathfrak {S}_{I\rightarrow II}({\tilde{x}},{\tilde{q}})$ in (230) also has the block upper triangular form

232 $$\begin{aligned} \begin{pmatrix} 1& *& *& *\\ 0& *& *& *\\ 0& *& *& *\\ 0& *& *& * \end{pmatrix}. \end{aligned}$$

The $3\times 3$ sub-matrices $\mathfrak {S}^{\text {red}}_{I\rightarrow II}$ in the right bottom have been worked out in [GGMn23], and they are given by

233a $$\begin{aligned} \mathfrak {S}^{\text {red}}_{I\rightarrow II}({\tilde{x}},{\tilde{q}})&=\frac{1}{2} \begin{pmatrix} 0& 1& 0\\ 0& 1& 1\\ -1& 0& 0 \end{pmatrix} {\textbf{J}}^\text {red}_{-1}({\tilde{x}};{\tilde{q}}^{-1})^T \begin{pmatrix} 1& 0& 0\\ 0& 0& 1\\ 0& 1& 0 \end{pmatrix} {\textbf{J}}^\text {red}_{-1}({\tilde{x}};{\tilde{q}}) \begin{pmatrix} 0& 0& -1\\ 1& -{\tilde{s}}& 0\\ 0& 1& 0 \end{pmatrix}, \quad |{\tilde{q}}|<1, \end{aligned}$$

where

234 $$\begin{aligned} {\tilde{s}}= s({\tilde{x}}), \end{aligned}$$

and ${\textbf{J}}^{\text {red}}(x,q)$ is given by (216). To calculate the first row of $\mathfrak {S}_{I\rightarrow II}({\tilde{x}},{\tilde{q}})$, we use the additional holomorphic block $D_m(x,q)$.

#### Conjecture 22

For every cone $R\subset \Lambda (x)$ and every $\tau \in R$, we have

235 $$\begin{aligned} D_0(x,q) = s_R(\Phi ^{(\sigma _0)})(x;\tau ) + \tau ^{-1/2}\frac{{\tilde{x}}^{1/2}-{\tilde{x}}^{-1/2}}{x^{1/2}-x^{-1/2}} \sum _{j=1}^3 M_{R,j}({\tilde{x}},{\tilde{q}})s_R(\Phi ^{(\sigma _j)})(x;\tau ),\nonumber \\ \end{aligned}$$

where $M_{R,j}({\tilde{x}},{\tilde{q}})$ ($j=1,2,3$) are ${\tilde{q}}$-series with coefficients in ${\mathbb {Z}}({\tilde{x}}^{\pm 1})$ depending on the cone *R*.

We present $M_{R,j}({\tilde{x}},{\tilde{q}})$ in terms of the row vector $M_R({\tilde{x}},{\tilde{q}}):= (M_{R,1},M_{R,2},M_{R,3})({\tilde{x}},{\tilde{q}})$, and it can be expressed in terms of a $3\times 3$ matrix $M^{(\sigma _0)}_R({\tilde{x}},{\tilde{q}})$

236 $$\begin{aligned} M_R({\tilde{x}},{\tilde{q}}) = \left( {\tilde{q}}D_0({\tilde{x}},{\tilde{q}}),{\tilde{q}}^2D_1({\tilde{x}},{\tilde{q}}),{\tilde{q}}^3D_2({\tilde{x}},{\tilde{q}})\right) M^{(\sigma _0)}_R({\tilde{x}},{\tilde{q}}). \end{aligned}$$

#### Conjecture 23

Equation (204) holds in the cones $R=I,II$ where the ${\tilde{q}}$- series $M_{R,j}({\tilde{q}})$ are given in terms of $M_R^{(0)}({\tilde{x}},{\tilde{q}})$ through (236) which are as follows

237a $$\begin{aligned} M^{(\sigma _0)}_{I}({\tilde{x}},{\tilde{q}})&= \begin{pmatrix} 1& -1& -{\tilde{s}}\,{\tilde{q}}\\ 0& -1& -1+{\tilde{s}}\,{\tilde{q}}\\ 0& 0& -{\tilde{q}}\end{pmatrix},\end{aligned}$$

237b $$\begin{aligned} M^{(\sigma _0)}_{II}({\tilde{x}},{\tilde{q}}) =&\begin{pmatrix} -1& 1& -{\tilde{s}}\,{\tilde{q}}\\ -1& 0& -1+{\tilde{s}}\,{\tilde{q}}\\ 0& 0& -{\tilde{q}}\end{pmatrix}. \end{aligned}$$

Equations (235), together with the reduced Stokes matrices $\mathfrak {S}^{\text {red}}_{I\rightarrow II}({\tilde{x}},{\tilde{q}})$ for $\Phi ^{(\sigma _j)}(x;\tau )$ ($j=1,2,3$), allow us to calculate entries in the first row of $\mathfrak {S}_{I\rightarrow II}({\tilde{x}},{\tilde{q}})$ by

238 $$\begin{aligned} \mathfrak {S}_{I\rightarrow II}({\tilde{x}},{\tilde{q}})_{0,j} = M_{I,j}({\tilde{x}},{\tilde{q}}) - \sum _{k=1}^3M_{II,k}({\tilde{x}},{\tilde{q}}) \mathfrak {S}^{\text {red}}_{I\rightarrow II}({\tilde{x}},{\tilde{q}})_{k,j},\quad j=1,2,3.\nonumber \\ \end{aligned}$$

In the following we list the first few terms of these ${\tilde{q}}$ -series.

239 $$\begin{aligned} \begin{aligned} \mathfrak {S}_{I\rightarrow II}({\tilde{x}},{\tilde{q}})_{0,1}&= -1+(1+{\tilde{s}}+{\tilde{s}}^2){\tilde{q}}-(-2{\tilde{s}}-{\tilde{s}}^2+{\tilde{s}}^3){\tilde{q}}^2-(1+{\tilde{s}}^4){\tilde{q}}^3 +O({\tilde{q}}^4),\\ \mathfrak {S}_{I\rightarrow II}({\tilde{x}},{\tilde{q}})_{0,2}&= 1-(1+2{\tilde{s}}+{\tilde{s}}^2){\tilde{q}}+(-{\tilde{s}}-{\tilde{s}}^2+2{\tilde{s}}^3){\tilde{q}}^2+(3{\tilde{s}}^2+{\tilde{s}}^3+{\tilde{s}}^4){\tilde{s}}^3 +O({\tilde{q}}^4),\\ \mathfrak {S}_{I\rightarrow II}({\tilde{x}},{\tilde{q}})_{0,3}&= -{\tilde{q}}+(1+{\tilde{s}}^2){\tilde{q}}^2+(3{\tilde{s}}+{\tilde{s}}^2){\tilde{q}}^3+O({\tilde{q}}^4). \end{aligned} \end{aligned}$$

Finally, we can factorise the global Stokes matrices $\mathfrak {S}_{I\rightarrow II}({\tilde{x}},{\tilde{q}})$ to obtain local Stokes matrices associated to individual singular points in $\Lambda$ and extract the associated Stokes constants. The Stokes constants for $\Phi ^{(\sigma _j)}(x;\tau )$ ($j=1,2,3$) are already given in [GGMn21, GGMn23]. We collect the Stokes contants for $\Phi ^{(\sigma _0)}(x;\tau )$ in the generating series

240 $$\begin{aligned} \textsf{S}^+_{0,j}({\tilde{x}},{\tilde{q}}) = \sum _{k\ge 0}\sum _{\ell } \mathcal {S}^{(k,\ell )}_{0,j}{\tilde{x}}^\ell {\tilde{q}}^k, \quad j=1,2,3. \end{aligned}$$

And we find that

241 $$\begin{aligned} \begin{aligned} \textsf{S}^+_{0,1}({\tilde{x}},{\tilde{q}})&= -1+{\tilde{q}}+{\tilde{s}}{\tilde{q}}^2+(-2+3{\tilde{s}}^2){\tilde{q}}^3+(2-{\tilde{s}}-2{\tilde{s}}^2+5{\tilde{s}}^3+2{\tilde{s}}^4){\tilde{q}}^4 +O({\tilde{q}}^5),\\ \textsf{S}^+_{0,2}({\tilde{x}},{\tilde{q}})&= 1-{\tilde{q}}-{\tilde{s}}{\tilde{q}}^2-(-2+3{\tilde{s}}^2){\tilde{q}}^3+(2-{\tilde{s}}-2{\tilde{s}}^2+5{\tilde{s}}^3+2{\tilde{s}}^4){\tilde{q}}^4 +O({\tilde{q}}^5),\\ \textsf{S}^+_{0,3}({\tilde{x}},{\tilde{q}})&= 0. \end{aligned} \end{aligned}$$

### An analytic extension of the Kashaev invariant and the colored Jones polynomial

In this section we discuss an analytic extension of the Kashaev invariant and of the colored Jones polynomial of the $\textbf{5}_2$ knot, illustrating Conjectures 1 and 2.

Recall that the colored Jones polynomial of the $\textbf{5}_2$ is given by

242 $$\begin{aligned} J^{\textbf{5}_2}_N(q) = \sum _{k=0}^{N-1}(-1)^k q^{-k(k+1)/2}(q^{1+N};q)_k(q^{1-N};q)_k H_k(q),\quad q = {\textrm{e}}^{2\pi {\textrm{i}}\tau }, \end{aligned}$$

where

243 $$\begin{aligned} H_k(q) = (-1)^kq^{k(k+3)/2}\sum _{k=0}^\ell q^{\ell (\ell +1)} \frac{(q;q)_k}{(q;q)_\ell (q;q)_{k-\ell }}. \end{aligned}$$

Let *u* be in a small neighborhood of the origin. It is related to $x = q^N$ and $\tau$ by

244 $$\begin{aligned} x = {\textrm{e}}^{u+2\pi {\textrm{i}}} = {\textrm{e}}^{u},\quad \tau = \frac{u+2\pi {\textrm{i}}}{2\pi {\textrm{i}}N}. \end{aligned}$$

Then *x* is close to 1 and $\tau$ is close to 1/*N*. Note that

245 $$\begin{aligned} N\tau = 1+\frac{u}{2\pi {\textrm{i}}} \end{aligned}$$

is the analogue of *n*/*k* in [Guk05], and here we are considering a deformation from the case of $n/k=1$. We also have

246 $$\begin{aligned} {\tilde{x}}= {\textrm{e}}^{\log (x)/\tau } = \exp \left( \frac{2\pi {\textrm{i}}N u}{u+2\pi {\textrm{i}}}\right) . \end{aligned}$$

When *x* is positive real, $\Phi ^{(\sigma _1)}(x;\tau )$ are not Borel summable along the positive real axis. Depending on whether $\tau$ is in the first or the fourth quadrant, we have

247a $$\begin{aligned} J^{\textbf{5}_2}_N(q)&= s_{I}(\Phi ^{(\sigma _0)})(x;\tau ) +\tau ^{-1/2}\frac{{\tilde{x}}^{1/2}-{\tilde{x}}^{-1/2}}{x^{1/2}-x^{-1/2}} \big ( s_{I}(\Phi ^{(\sigma _1)})(x;\tau ) -(1+{\tilde{x}})s_{I}(\Phi ^{(\sigma _2)})(x;\tau )\nonumber \\&\quad -(1+{\tilde{x}})s_{I}(\Phi ^{(\sigma _3)})(x;\tau ) \big ) \end{aligned}$$

247b $$\begin{aligned}&= s_{IV}(\Phi ^{(\sigma _0)})(x;\tau ) +\tau ^{-1/2} \frac{{\tilde{x}}^{1/2}-{\tilde{x}}^{-1/2}}{x^{1/2}-x^{-1/2}} \big ( s_{IV}(\Phi ^{(\sigma _1)})(x;\tau ) \nonumber \\&\quad +(1+{\tilde{x}}^{-1})s_{IV}(\Phi ^{(\sigma _2)})(x;\tau )-(1+{\tilde{x}})s_{IV}(\Phi ^{(\sigma _3)})(x;\tau ) \big ). \end{aligned}$$

The two equations (247a), (247b) are related by the Stokes discontinuity formula

248 $$\begin{aligned} \text {disc}_0 \Phi ^{(\sigma _1)}(x;\tau ) = s_I(\Phi ^{(\sigma _1)})(x;\tau ) - s_{IV}(\Phi ^{(\sigma _1)})(x;\tau )= (2+{\tilde{x}}+{\tilde{x}}^{-1}) s(\Phi ^{(\sigma _2)})(x;\tau ).\nonumber \\ \end{aligned}$$

Combined, they imply

249 $$\begin{aligned} J_N^{\textbf{5}_2}(q)&= s_{\text {med}}(\Phi ^{(\sigma _0)})(x;\tau )+\tau ^{-1/2} \frac{{\tilde{x}}^{1/2}-{\tilde{x}}^{-1/2}}{x^{1/2}-x^{-1/2}} \big ( s_{\text {med}}(\Phi ^{(\sigma _1)})(x;\tau ) -(1+{\tilde{x}})s_{\text {med}}(\Phi ^{(\sigma _3)})(x;\tau )\nonumber \\&\quad -\frac{{\tilde{x}}-{\tilde{x}}^{-1}}{2}s_{\text {med}}(\Phi ^{(\sigma _2)})(x;\tau ) \big ) \end{aligned}$$

which is the assertion of Conjecture 2.

### A new state-integral for the ${\textbf{5}_2}$ knot?

In the case of the figure eight knot, the new state-integral was obtained by first writing down an integral formula for its colored Jones polynomial, in Habiro form, and then changing the integration contour to pick the contribution from the poles in the lower half plane. This led in particular to the “inverted” Habiro series $\mathcal {C}_0(x,q)$ in (146). Although we do not have a similar complete theory for the ${\textbf{5}_2}$ knot, we can however write down an integral formula for its colored Jones polynomial which lead, after a change of contour, to the corresponding inverted Habiro series. In fact, it is possible to write such an integral for all twist knots $K_p$ (the ${\textbf{5}_2}$ knot corresponds to $p=2$).

Let us then consider the colored Jones polynomial of the twist knot $K_p$ in Habiro’s form [Mas03]:

250 $$\begin{aligned} J^{K_p}_N (q;x)= \sum _{n=0}^{N-1} C^{K_p}_n (q) (qx;q)_n (q x^{-1};q)_n, \end{aligned}$$

where

251 $$\begin{aligned} C^{K_p}_n(q)= -q^n \sum _{k=0}^n (-1)^k q^{(p+1/2)k(k+1) +k } (q^{2k+1}-1) {(q;q)_n \over (q;q)_{n+k+1} (q;q)_{n-k}}. \end{aligned}$$

It is easy to see that (250) can be written as a double contour integral

252 $$\begin{aligned} \int _{\mathcal {A}_z} \int _{\mathcal {A}_w} \mathcal {I}_{K_p}(z,w) {\textrm{d}} z {\textrm{d}} w, \end{aligned}$$

where

253 $$\begin{aligned} \begin{aligned} \mathcal {I}_{K_p} (z, w)&=-{\Phi }_{{\textsf{b}}}^{-1} \left( z-{{\textrm{i}}\over 2 {{\textsf{b}}}} + u \right) {\Phi }_{{\textsf{b}}}^{-1} \left( z-{{\textrm{i}}\over 2 {{\textsf{b}}}} - u\right) \\&\quad {\Phi }_{{\textsf{b}}}^{-1} \left( z-{{\textrm{i}}\over 2 {{\textsf{b}}}} \right) {\Phi }_{{\textsf{b}}}\left( z-w+ {{\textrm{i}}{{\textsf{b}}}\over 2} -{{\textrm{i}}\over 2 {{\textsf{b}}}} \right) \\&\quad \times {\Phi }_{{\textsf{b}}}\left( z+w+ {{\textrm{i}}{{\textsf{b}}}\over 2} -{{\textrm{i}}\over 2{{\textsf{b}}}} \right) {\textrm{e}}^{-2 \pi {\textrm{i}}(p+1/2)(w+ {{\textrm{i}}\over 2 {{\textsf{b}}}})^2} \left( {\textrm{e}}^{ 2 \pi {{\textsf{b}}}(z+w)} - {\textrm{e}}^{ 2 \pi {{\textsf{b}}}(z-w)} \right) \\&\quad \tanh \left( {\pi z \over {{\textsf{b}}}} \right) \tanh \left( {\pi w \over {{\textsf{b}}}} \right) , \end{aligned} \end{aligned}$$

and the contours $\mathcal {A}_{z,w}$ encircle the poles of the form (71) in the upper complex planes of the *z* and the *w* variables, respectively. We can now deform the contour to pick the poles in the lower half planes of *z*, *w*. The contribution from the simple poles of the $\tanh$ functions in those half planes can be easily computed, and one finds in this way the inverted Habiro series,

254 $$\begin{aligned} \begin{aligned} \mathcal {C}_{K_p} (q, x)&={1 \over (x^{1\over 2}- x^{-{1\over 2}})^2} \sum _{n \ge 0} { q^{n (n+1)/2} \over (q x;q)_n (q x^{-1};q)_n} \\&\quad \times \sum _{k \ge n} q^{n (n+1)/2 + (p+1/2) k(k+1)- (n+k)(n+k+1)/2-n} (q^k - q^{-k-1}) {(q;q)_{n+k} \over (q;q)_n (q;q)_{k-n}}. \end{aligned} \end{aligned}$$

This gives a general formula for all twist knots which agrees with the results of [Par] for $p=2$ (the ${\textbf{5}_2}$ knot) and $p=3$ (the ${\textbf{7}_2}$ knot).

It might be possible to find appropriate integration contours so that the integral of $\mathcal {I}_{K_p} (z, w)$ converges and provides the sought-for new state-integral which sees the series $\Phi ^{(\sigma _0)} (x, \tau )$, as it happened in the case of the ${\textbf{4}_1}$ knot. In the case of the ${\textbf{5}_2}$ knot, these contours do exist and lead to a well-defined integral. We expect that an evaluation of such an integral by summing over the appropriate set of residues will give the inverted Habiro series (254), together with additional contributions, as in (146). However, the fact that the integrals are two-dimensional makes them more difficult to analyze. We expect to come back to this problem in the near future.

## Appendix A. *q*-Series Identities

In this appendix we will sketch the proofs of some *q*-series identities that appear in Conjecture 19. Since this not one of the main themes of the paper, our presentation will be rather brief. Our proofs of the *q*-hypergeometric identities will use the algorithmic approach of the Wilf-Zeilberger theory (see [WZ92, PWZ96]) and the computer implementation by Koutschan [Kou10]).

We outline part of the proof of Conjecture 19 for $|q|<1$, namely

255 $$\begin{aligned} \begin{aligned} qQ^{(0,0)}_{0}(q)&= (q;q)_{\infty }\sum _{n=0}^{\infty }\frac{q^{n(n+1)}}{(q;q)_{n}^{3}}\\ qQ_{1}^{(0,0)}(q)&= (q;q)_{\infty }\sum _{n=0}^{\infty }(2-q^{n})\frac{q^{n(n+1)}}{(q;q)_{n}^{3}}\\ qQ_{2}^{(0,0)}(q)&= (q;q)_{\infty }\sum _{n=0}^{\infty } \left( (3+q^{-1})-(2+2q^{-1})q^{n}+q^{2n-1}\right) \frac{q^{n(n+1)}}{(q;q)_{n}^{3}} \end{aligned} \end{aligned}$$

### Proof

The definition of $Q_{m}^{(0,0)}(q)$ gives that

256 $$\begin{aligned} qQ_{m}^{(0,0)}(q)= f_{-m-1,0}(q) \end{aligned}$$

where

257 $$\begin{aligned} \begin{aligned} f_{m,p}(q)&= \sum _{n,k=0}^{\infty }(-1)^{k}q^{n(n+1)+k(k+1)/2-nk+mn+pk} \frac{(q;q)_{n+k}}{(q;q)_{n}^{3}(q;q)_{k}}\\&=\sum _{k=0}^{\infty }f_{m,p,k}(q) \end{aligned} \end{aligned}$$

with

258 $$\begin{aligned} f_{m,p,k}(q) = \sum _{n=0}^{\infty }(-1)^{k}q^{n(n+1)+k(k+1)/2-nk+mn+pk} \frac{(q;q)_{n+k}}{(q;q)_{n}^{3}(q;q)_{k}} . \end{aligned}$$

Likewise, we define

259 $$\begin{aligned} h_{m,p}(q)= &  (q;q)_{\infty }\sum _{n=0}^{\infty }\frac{q^{n(n+1)+pn+mn+mp}}{(q;q)_{n+p}^{2}(q;q)_{n}}\nonumber \\= &  \frac{1}{(q;q)_{\infty }}\sum _{n,k,\ell =0}^{\infty }(-1)^{k+\ell }\frac{q^{n(n+1)+k(k+1)/2 +\ell (\ell +1)/2+pn+pm+pk+p\ell +nk+mn}}{(q;q)_{n}(q;q)_{k}(q;q)_{\ell }}\nonumber \\= &  \sum _{k=0}^{\infty }h_{m,p,k}(q) \end{aligned}$$

where

260 $$\begin{aligned} h_{m,p,k}(q) = \frac{1}{(q;q)_{\infty }}\sum _{n+j+\ell +m=k}^{\infty }(-1)^{j+\ell } \frac{q^{n(n+1)+j(j+1)/2 +\ell (\ell +1)/2+pn+pm+pj+p\ell +nj+mn}}{(q;q)_{n}(q;q)_{j}(q;q)_{\ell }} .\nonumber \\ \end{aligned}$$

Therefore, we have

261 $$\begin{aligned} f_{-1,0}(q) = qQ^{(0,0)}_{0}(q) \quad \text {and} \quad h_{0,0}(q) = (q;q)_{\infty }\sum _{n=0}^{\infty }\frac{q^{n(n+1)}}{(q;q)_{n}^{3}} . \end{aligned}$$

This implies that the first equality in (255) follows from the $p=0$ specialization of

262 $$\begin{aligned} f_{-1,p}(q)=h_{0,p}(q), \qquad (p \in \mathbb {Z}) . \end{aligned}$$

This in turn follows (using Equations (257) and (259)) from the following

263 $$\begin{aligned} f_{-1,p,k}(q)=h_{0,p,k}(q), \qquad (p \in \mathbb {Z}, k \in \mathbb {N}) . \end{aligned}$$

Equation (258) expresses the two-variable *q*-holonomic function $f_{-1,p,k}(q)$ as a one dimensional sum of a three variable proper *q*-hypergeometric function. It follows from [Kou10] that the annihilator ideal of $F_{k,p}(q):=f_{-1,p,k}(q)$ is generated by the recursion relations

264 $$\begin{aligned} &  -q^k F_{p, k}(q) + F_{1 + p, k}(q)=0, \end{aligned}$$

265 $$\begin{aligned} &  q^{2 + k + 2 p} (-1 + q^{1 + k})^2 F_{p, k}(q)\nonumber \\ &  \quad + q^{2 + k + p} (-3 + q^{1 + k} + q^{2 + k}) F_{p, 1 + k}(q) + (-1 + q^{2 + k}) F_{p, 2 + k}(q)=0 \end{aligned}$$

This coincides with the annihilator ideal of $h_{0,p,k}(q)$. Thus, the equality (263) for $p,k \in \mathbb {Z}$ with $k \ge 0$ follows from the two special cases $(p,k)=(0,0)$ and $(p,k)=(0,1)$, that is from the identities

266 $$\begin{aligned} \begin{aligned} \sum _{n=0}^{\infty }\frac{q^{n^{2}}}{(q;q)_{n}^{2}}&= \frac{1}{(q;q)_{\infty }}\\ \frac{1}{1-q}\sum _{n=0}^{\infty }\frac{q^{n^{2}-n+1}(q^{n+1}-1)}{(q;q)_{n}^{2}}&= \frac{q^{2}-2q}{(q;q)_{\infty }(1-q)} \end{aligned} \end{aligned}$$

The first one of the above identities is due to Euler and can be derived using generating functions of partitions. The second one follows from the *q*-holonomic system

267 $$\begin{aligned} g_{m}(q)=\sum _{n=0}^{\infty }\frac{q^{n^{2}+mn}}{(q;q)_{n}^{2}} \qquad \text {with}\qquad g_{m}(q)-2g_{m+1}(q)+(1-q^{m+1})g_{m+2}(q)=0.\nonumber \\ \end{aligned}$$

This concludes the proof of the first identity in (255). The remaining two identities follow (using the above steps) from the following ones

268 $$\begin{aligned} \begin{aligned} f_{-2,p,k}(q)&= 2h_{0,p,k}(q)-h_{1,p,k}(q),\\ f_{-3,p,k}(q)&= (3+q^{-1})h_{0,p,k}(q)-(2+2q^{-1})h_{1,p,k}(q)+q^{-1}h_{2,p,k}(q). \end{aligned} \end{aligned}$$

This concludes the sketch of the proof of (255). $\square$

In the course of the proof, we came up with the following conjecture which expresses $f_{m,p}(q)$ as $\mathbb {Z}[q^{\pm 1}]$-linear combinations of $h_{m,p}(q)$.

### Conjecture 24

For $m \ge 0$ we have:

269 $$\begin{aligned} \begin{aligned} f_{m,p}(q)&= \sum _{k,i=0}^{\infty }(-1)^iq^{i(i+1)/2+k} \frac{(q;q)_{m+k+i}}{(q;q)_{m}(q;q)_{i}(q;q)_{k}}h_{k,p}(q),\\ f_{-1-m,p}(q)&= \sum _{k=0}^{m}\sum _{i=0}^{m-k}(-1)^iq^{i(i+1)/2+k} \frac{(q^{-1};q^{-1})_{m}}{(q^{-1};q^{-1})_{m-i-k}(q;q)_{i}(q;q)_{k}}h_{k,p}(q) . \end{aligned} \end{aligned}$$

## Footnotes

1 A similar phenomenon was observed by Hatsuda–Okuyama [HO15].

2 for a suitable diagonal matrix $\Delta (\tau )$ of weights.

3 $G_m^{(1)}(q)$ defined here is one half of $G^1_m(q)$ in [GGMn21].

4 The matrices ${\textbf{J}}^\text {red}_m(q)$ are related to the Wronskians $W_m(q)$ in [GGMn21, GGMn23] by

$$\begin{aligned} {\textbf{J}}^\text {red}_m(q) = \begin{pmatrix} 0& 0& 1\\ 0& 1& 0\\ 1& 0& 0 \end{pmatrix} W_m(q)^T. \qquad \qquad (158) \end{aligned}$$

5 Our $H_{-1}(q)$ agrees with the one defined in [Par] when $|q|<1$, however differs when $|q|>1$.

6 The series $\Phi ^{(\sigma _j)}(\tau )$ ($j=1,2,3$) are related to the series in [GGMn21, GGMn23], which we will denote by $\Phi ^{(\sigma _j)}_{\text {GGM}}(\tau )$, by a common prefactor

$$\begin{aligned} \Phi ^{(\sigma _j)}(\tau ) = {\textrm{i}}{\textrm{e}}^{-\frac{\pi {\textrm{i}}}{12}(\tau +\tau ^{-1})-2\pi {\textrm{i}}\tau } \Phi ^{(\sigma _j)}_{\text {GGM}}(\tau ),\quad j=1,2,3.\qquad \qquad (187) \end{aligned}$$

The Stokes constants associated to the Borel resummation of $\Phi ^{(\sigma _j)}_{\text {GGM}}(\tau )$ are not changed. The additional prefactor is introduced so that the Stokes automorphism between $\Phi ^{(\sigma _0)}(\tau )$ and $\Phi ^{(\sigma _j)}(\tau )$ ($j=1,2,3$) can be presented in an elegant form, and is also dictated by positions of singularities of Borel transform of $\Phi ^{(\sigma _0)}(\tau )$.

7 The matrices ${\textbf{J}}^\text {red}_m(x,q)$ are related to the Wronskians $W_m(x,q)$ in [GGMn21, GGMn23] by

215 $$\begin{aligned} {\textbf{J}}^\text {red}_m(x,q) = W_m(x,q)^T. \end{aligned}$$

8 The series $\Phi ^{(\sigma _j)}(x;\tau )$ ($j=1,2,3$) are related to the series in [GGMn21, GGMn23], which we will denote by $\Phi ^{(\sigma _j)}_{\text {GGM}}(x;\tau )$, by a common prefactor

$$\begin{aligned} \Phi ^{(\sigma _j)}(x;\tau ) = {\textrm{i}}{\textrm{e}}^{-\frac{\pi {\textrm{i}}}{12}(\tau +\tau ^{-1})-2\pi {\textrm{i}}\tau } \Phi ^{(\sigma _j)}_{\text {GGM}}(x;\tau ),\quad j=1,2,3.\qquad \qquad (222) \end{aligned}$$

The Stokes constants associated to the Borel resummation of $\Phi ^{(\sigma _j)}_{\text {GGM}}(\tau )$ are not changed.
